# Lead Optimization of a Butyrylcholinesterase Inhibitor for the Treatment of Alzheimer’s Disease

**DOI:** 10.1021/acs.jmedchem.5c00577

**Published:** 2025-06-02

**Authors:** Urban Košak, Nika Strašek Benedik, Damijan Knez, Simon Žakelj, Jurij Trontelj, Anja Pišlar, Selena Horvat, Aljoša Bolje, Neža Žnidaršič, Neža Grgurevič, Tanja Švara, Jakob Kljun, Anna Skrzypczak-Wiercioch, Bingbing Lv, Yucheng Xiong, Qinjie Wang, Rui Bian, Jikuan Shao, José Dias, Florian Nachon, Xavier Brazzolotto, Jure Stojan, Haopeng Sun, Kinga Sałat, Stanislav Gobec

**Affiliations:** † Faculty of Pharmacy, 37663 University of Ljubljana, Aškerčeva cesta 7, 1000 Ljubljana, Slovenia; ‡ Institute of Preclinical Sciences, Veterinary Faculty, 63721 University of Ljubljana, Gerbičeva 60, 1000 Ljubljana, Slovenia; § Institute of Pathology, Wild Animals, Fish and Bees, Veterinary Faculty, University of Ljubljana, Gerbičeva 60, 1000 Ljubljana, Slovenia; ∥ Faculty of Chemistry and Chemical Technology, University of Ljubljana, Večna pot 113, 1000 Ljubljana, Slovenia; ⊥ 49563 University Centre of Veterinary Medicine JU-UA, University of Agriculture in Krakow, 24/28 Mickiewicza St., 30-059 Krakow, Poland; # School of Pharmacy, 56651 China Pharmaceutical University, Nanjing 211198, People’s Republic of China; ∇ School of Pharmacy, 66478 Nanjing University of Chinese Medicine, Nanjing 210023, People’s Republic of China; ○ Département de Toxicologie et Risques Chimiques, 195617 Institut de Recherche Biomédicale des Armées, 91220 Brétigny sur Orge, France; ◆ Institute of Biochemistry, Faculty of Medicine, University of Ljubljana, Vrazov trg 2, 1000 Ljubljana, Slovenia; ¶ Faculty of Pharmacy, Jagiellonian University Medical College, 9 Medyczna St., 30-688 Krakow, Poland

## Abstract

Butyrylcholinesterase (BChE) is a promising drug target for alleviating the symptoms of canine cognitive dysfunction (CCD) and Alzheimer’s disease (AD). We have recently developed lead compound **2**, a racemic, nanomolar BChE inhibitor with procognitive effects in mice with scopolamine-induced AD-like symptoms and dogs suffering from CCD. To overcome its modest brain exposure, we developed compound **(** *R* **)-(−)-3**, a more potent BChE inhibitor with a 7-fold higher in vivo brain exposure. It has procognitive effects in mice with scopolamine-induced AD-like symptoms and, superior to compound **2**, also in mice with Aβ_1–42_-induced AD-like symptoms. Compound **(** *R* **)-(−)-3** produces no cholinergic adverse effects or motor deficits and has no acute toxic effects in mice. This makes sulfonamide **(** *R* **)-(−)-3** an optimized lead compound for alleviating the symptoms of AD.

## Graphical abstract

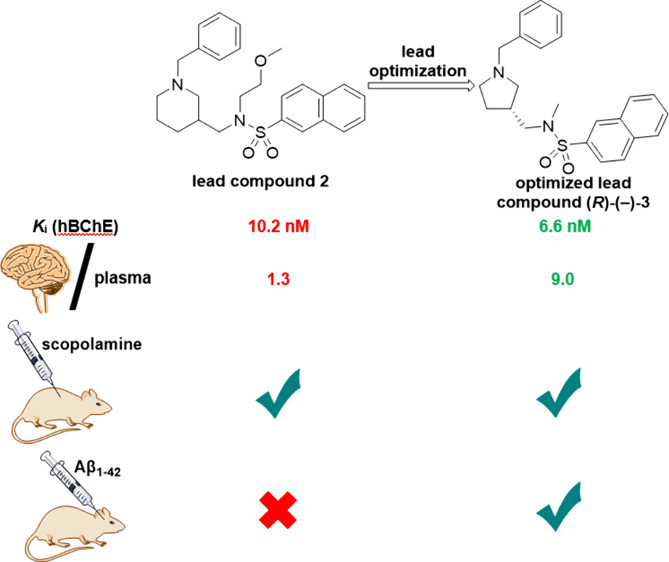

## Introduction

Alzheimer’s disease (AD) is a neurodegenerative brain disease.^1^ As the population continues to age, the number of AD patients is expected to rise and is anticipated to reach 150 million by 2050.^2^ AD is defined by β-amyloid (Aβ)-containing extracellular plaques, tau-containing intracellular neurofibrillary tangles, oxidative stress and cholinergic dysfunction.^3^ These pathologies reduce the levels of the neurotransmitter acetylcholine (ACh)­in the brain, leading to cognitive disabilities, learning deficits and severe memory impairment in patients.^4–6^

In the neuronal junction, cholinergic neurotransmission is stopped by the hydrolysis of ACh to acetate and choline. This reaction is catalyzed by cholinesterases (ChEs): acetylcholinesterase (AChE) and butyrylcholinesterase (BChE).^7^ In the healthy adult brain, the enzymatic activity of AChE is dominant (i.e., this enzyme hydrolyzes approximately 80% of ACh), while BChE plays a supporting role and hydrolyzes approximately 20% of the neurotransmitter.^8^ The current arsenal of drugs for the treatment of AD contains 6 drugs: donanemab, lecanemab, memantine, donepezil, rivastigmine and galantamine. Donanemab and lecanemab are monoclonal antibodies that target Aβ in the brain,^9,10^ memantine is an antagonist of *N*-methyl-d-aspartate (NMDA) receptors,^11^ donepezil is a selective AChE inhibitor,^12^ rivastigmine inhibits both ChEs^13^ and galantamine is a selective AChE inhibitor and allosteric modulator of nicotinic ACh receptors (nAChRs).^14^

It was initially thought that the enzymatic activity of BChE was unimportant for a normally functioning central nervous system (CNS).^8^ It is now known that this is not true, because the enzymatic activity of BChE is associated with glial cells, neuronal and vascular structures, with BChE-positive neurons located in the neocortex, amygdala and thalamus.^15–17^ Also, BChE efficiently hydrolyzes ACh in the healthy adult brain and acts as the only ChE to maintain ChE function in AChE nullizygote mice.^18–22^

In late stages of AD, the supporting role of BChE in ACh hydrolysis changes to a leading role.^23–26^ Because of this phenomena and the procognitive effects of selective BChE inhibitors in vivo, without peripheral (parasympathomimetic) adverse effects,^27–36^ which are typical for AChE inhibitors and limit their use,^37–39^ selective BChE inhibitors could be used to alleviate the symptoms of cholinergic dysfunction and improve cognitive functions in patients suffering from AD.

In 2014, we reported the use of virtual screening to discover hit compound **1** (Figure 1A), a reversible, potent and selective inhibitor of human BChE (hBChE).^40^ We used compound **1** and developed sulfonamide **2** (Figure 1A),^30^ which among the large number of selective hBChE inhibitors reported,^27–32,34–36,41–46^ is a very promising selective hBChE inhibitor. Sulfonamide **2** improves memory and learning abilities of mice with scopolamine-induced AD-like symptoms^30^ and improves cognitive functions and quality of life of dogs suffering from canine cognitive dysfunction (CCD),^33^ which has many similarities with AD in humans.^47,48^ Most importantly, hBChE inhibitor **2** does not produce the cholinergic adverse effects in mice^30^ or dogs,^33^ that are typical of approved drugs that increase ACh levels in the brain.^37–39^

**Figure 1:**
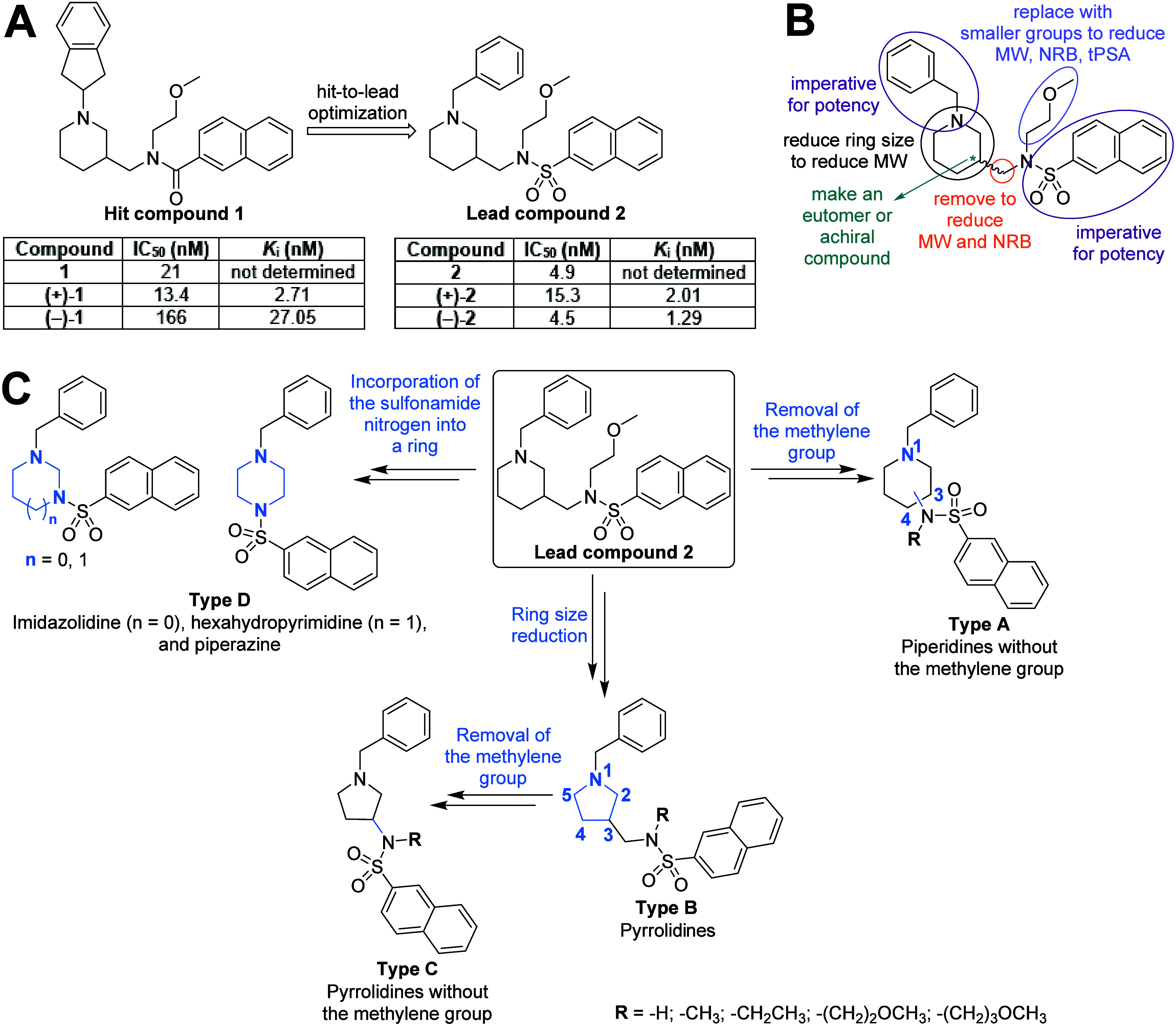
(A) Structures of hit compound **1** and lead compound **2** and their inhibitory potencies against human BChE. (B) Structural modification opportunities for reducing the MW, NRB and tPSA of lead compound **2**. (C) Design of type A–D inhibitors. Structural changes in each step are shown in blue.

However, lead compound **2** (Figure 1) is a racemate and has modest brain exposure - the brain-to-plasma ratio in rats is 0.44.^30^ Our goal in this project was to optimize lead compound **2** to an achiral or optically pure compound with equal or improved hBChE inhibitory potency, good selectivity over human AChE (hAChE), improved brain exposure and superior procognitive effects in vivo. We report the optimization of lead compound **2** into compound **(** *R* **)-(−)-3**, which is a more potent hBChE inhibitor with enhanced brain exposure compared to **2**. Moreover, **(** *R* **)-(−)-3** is superior to parent sulfonamide **2** as it improves cognitive functions in mice with scopolamine-induced cognitive deficits and also in mice with Aβ_1–42_-induced cognitive deficits. Just like compound **2**,^30^ compound **(** *R* **)-(−)-3** does not cause cholinergic adverse effects (salivation, diarrhea, and reduced locomotor activity)^37–39^ in mice and does not show acute toxic effects in these animals.

## Results and Discussion

### Design of the Inhibitors

Reducing the molecular weight (MW), number of rotatable bonds (NRB) and topological polar surface area (tPSA) is commonly used to improve the brain exposure of a (lead) compound.^49^ Several structural modification opportunities to reduce these parameters of compound **2** (Figure 1B) have been identified, and type A–D inhibitors (Figure 1C) have been designed with special attention devoted to the generation of inhibitors with equal or reduced MW, NRB and tPSA compared to lead compound **2**. Our previous structure–activity relationships (SARs) studies have shown that the basic *N*-benzyl and the naphthalene-2-sulfonamide groups are essential for potent hBChE inhibition^30^ (Figure 1B). Therefore, these two groups were preserved in all type A–D inhibitors. To investigate whether and how the methylene group between the piperidine ring and sulfonamide nitrogen effects human (h)­ChE inhibition, type A inhibitors were designed. This methylene group was therefore removed from lead compound **2**. Piperidines with the 1,3- as well as the 1,4-disubstitution pattern were designed to explore the effect of the ring substitution pattern. Scaffold hopping^50^ was used, more specifically ring size reduction of the six-membered piperidine of lead compound **2** into the five-membered pyrrolidine to design type B inhibitors and thus investigate the effect of the size of the ring. The type C inhibitors were designed with the same goal as the type A inhibitors: to explore the effect of the methylene group, in this case between the pyrrolidine ring and the sulfonamide nitrogen. In type A–C inhibitors, the alkyl group [−(CH_2_)_2_OCH_3_] on the sulfonamide nitrogen of lead compound **2** was retained, removed or replaced with shorter (methyl and ethyl) or longer [−(CH_2_)_3_OCH_3_] alkyl groups to investigate the effects of their presence and length. Again, scaffold hopping^50^ was used to design type D inhibitors with the aim to investigate the effects of incorporating the sulfonamide nitrogen into a ring. The piperidine of lead compound **2** was therefore replaced by the imidazolidine, hexahydropyrimidine, and piperazine (Figure 1C).

### Synthesis

Synthesis of type A inhibitors **4**–**13** is presented in Schemes 1 and 2. Sulfonamides without a substituent on their sulfonamide nitrogen (compounds **4** and **5**) were prepared from amines **14** and **15**, respectively, by reacting them with naphthalene-2-sulfonyl chloride.^30^ Sulfonamides with a substituent on their sulfonamide nitrogen (compounds **6**–**13**) were synthesized from benzyloxycarbonyl (Cbz) protected ketones **16** and **17**. In the first step, compounds **16** and **17** were reacted with the appropriate amine [CH_3_NH_2_, CH_3_CH_2_NH_2_, H_2_N­(CH_2_)_2_OCH_3_ or H_2_N­(CH_2_)_3_OCH_3_][Bibr ref51] to produce secondary amines **18**–**25**. These amines (**18**–**25**) were then reacted with naphthalene-2-sulfonyl chloride^30^ to produce sulfonamides **26**–**33**, from which the Cbz protecting group was removed using classic catalytic hydrogenation with gaseous hydrogen and a catalytic amount of 10% Pd/C to yield amines **34**–**41**. In the final step, amines **34**–**41** were benzylated with benzyl bromide to produce the final *N*-benzyl piperidines **6**–**13** (Schemes 1 and 2).

**Scheme 1:**
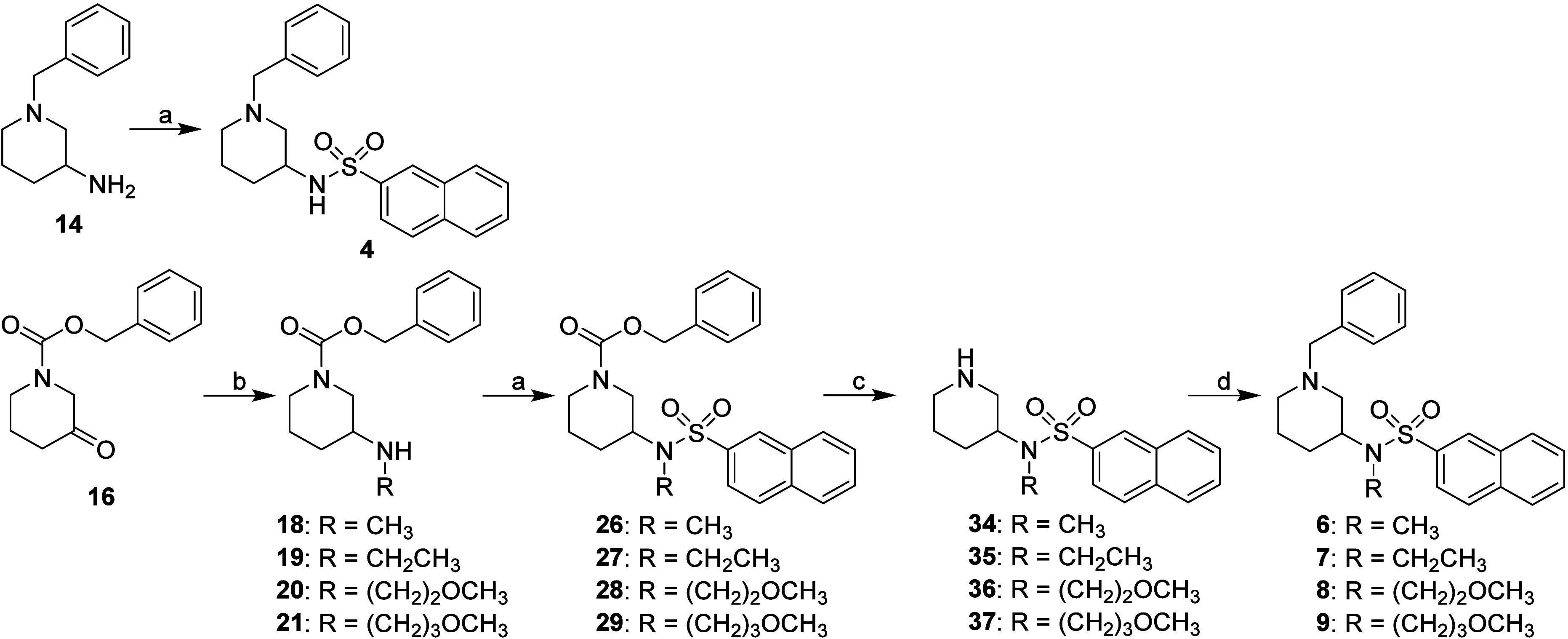
Synthesis of 1,3-Disubstituted Type A Inhibitors^a^

a Reagents and conditions: (a) naphthalene-2-sulfonyl chloride, (CH_3_CH_2_)_3_N, CH_2_Cl_2_, 0 °C to room temperature, 24 h, 24 to 96%; (b) corresponding amine, NaBH­(OAc)_3_, AcOH, 1,2-dichloroethane, room temperature, under argon, 24 h, 80 to 92% crude; (c) H_2_, Pd/C (10 wt %), CH_3_OH or mixture of CH_3_OH and THF, room temperature, 24 h, 82 to 92% crude; (d) PhCH_2_Br, Cs_2_CO_3_, acetone, room temperature, 24 h, 84 to 91%.

**Scheme 2:**
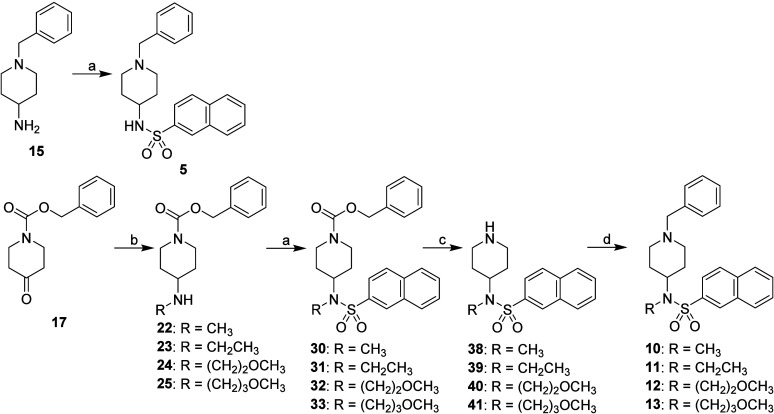
Synthesis of 1,4-Disubstituted Type A Inhibitors^a^

a Reagents and conditions: (a) naphthalene-2-sulfonyl chloride, (CH_3_CH_2_)_3_N, CH_2_Cl_2_, 0 °C to room temperature, 24 h, 73 to 96%; (b) corresponding amine, NaBH­(OAc)_3_, AcOH, 1,2-dichloroethane, room temperature, under argon, 24 h, 92 to 98% crude; (c) H_2_, Pd/C (10 wt %), CH_3_OH or mixture of CH_3_OH and THF, room temperature, 24 h, 86 to 95% crude; (d) PhCH_2_Br, Cs_2_CO_3_, acetone, room temperature, 24 h, 37 to 91%.

Synthesis of type B inhibitors is presented in Scheme 3. The sulfonamide without a substituent on its sulfonamide nitrogen (compound **42**) was prepared from primary amine **43** by reacting it with naphthalene-2-sulfonyl chloride.^30^ Sulfonamides with a substituent on its sulfonamide nitrogen (compounds **3** and **44**–**46**) were synthesized from carboxylic acid **47**. In the first step, compound **47** was treated with benzoyl chloride to provide carboxylic acid **48**. Compound **48** was then converted into diamides **49**–**52** with the appropriate primary amines [i.e., CH_3_NH_2_, CH_3_CH_2_NH_2_, H_2_N­(CH_2_)_2_OCH_3_ or H_2_N­(CH_2_)_3_OCH_3_] and coupling reagent *O*-(benzotriazol-1-yl)-*N,N,N′,N′*-tetramethyluronium tetrafluoroborate (TBTU).^52^ LiAlH_4_ in anhydrous THF at reflux^52,53^ was used to convert diamides **49**–**52** to diamines **53**–**56**, which were then treated with naphthalene-2-sulfonyl chloride^30^ to yield the final compounds **3** and **44**–**46** (Scheme 3).

**Scheme 3:**
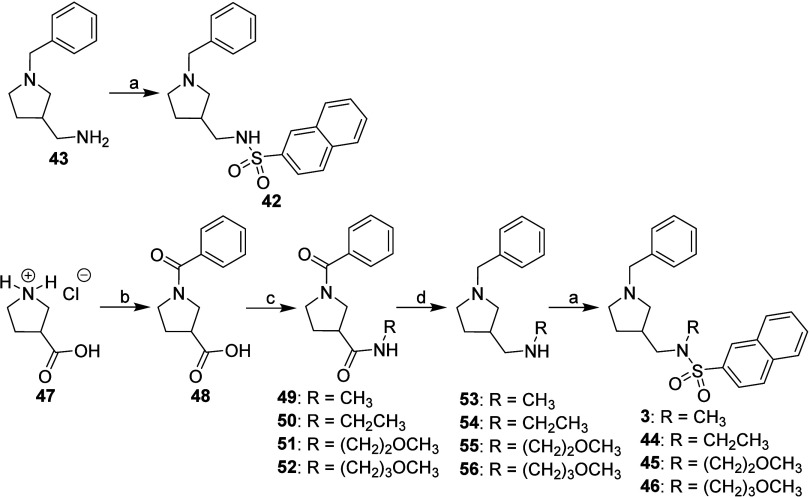
Synthesis of Type B Inhibitors [(Pyrrolidin-3-yl)­methylene Derivatives]^a^

a Reagents and conditions: (a) naphthalene-2-sulfonyl chloride, (CH_3_CH_2_)_3_N, CH_2_Cl_2_, 0 °C to room temperature, 24 h, 79 to 93%; (b) PhCOCl, 1 M NaOH aqueous solution, 1,4-dioxane, 0 °C to room temperature, 24 h, 83%; (c) corresponding amine, (CH_3_CH_2_)_3_N, TBTU, 0 °C to room temperature, 24 h, 87 to 98%; (d) LiAlH_4_, anhydrous THF, room temperature to reflux, 3 h, 96 to 98% crude.

The reagents and conditions used to prepare type A inhibitors **4**–**13**, were also used to synthesize type C inhibitors **57**–**61** from amine **62** and ketone **63** (Scheme 4).

**Scheme 4:**
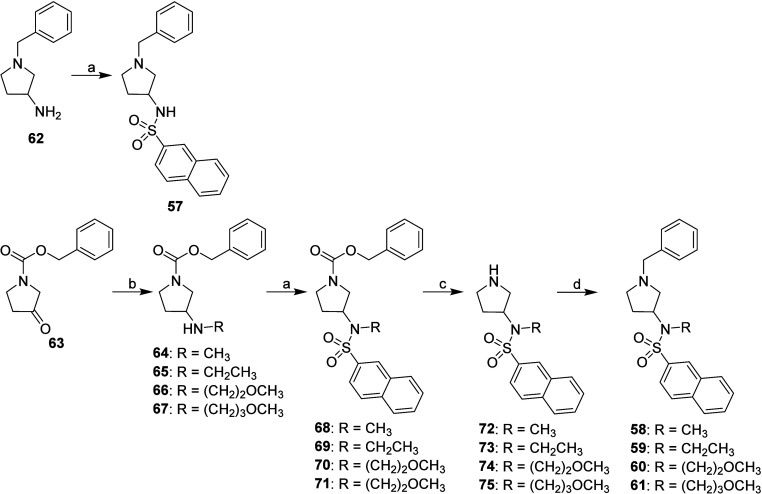
Synthesis of Type C Inhibitors (Pyrrolidine Derivatives)^a^

a Reagents and conditions: (a) naphthalene-2-sulfonyl chloride, (CH_3_CH_2_)_3_N, CH_2_Cl_2_, 0 °C to room temperature, 24 h, 32 to 97%; (b) corresponding amine, NaBH­(OAc)_3_, AcOH, 1,2-dichloroethane, room temperature, under argon, 24 h, 92 to 98% crude; (c) H_2_, Pd/C (10 wt %), CH_3_OH or mixture of CH_3_OH and THF, room temperature, 24 h, 79 to 91% crude; (d) PhCH_2_Br, Cs_2_CO_3_, acetone, room temperature, 24 h, 30 to 51%.

The synthesis of type D inhibitors (compounds **76**–**78**) is presented in Scheme 5. The imidazolidine derivative **76** was synthesized by first preparing 1-benzylimidazolidine (compound **79**) from *N* ^1^-benzylethane-1,2-diamine (compound **80**) with paraformaldehyde [(HCHO)_*n*_].^54^ Compound **79** was then treated with naphthalene-2-sulfonyl chloride^30^ to yield compound **76**. This same reaction was used to prepare hexahydropyrimidine derivative **77** from commercially available 1-benzylhexahydropyrimidine (compound **81**). Piperazine derivative **78** was synthesized by first preparing sulfonamide **82** from *tert*-butyl piperazine-1-carboxylate (compound **83**) and naphthalene-2-sulfonyl chloride.^30^ The *tert*-butyloxycarbonyl protecting group (BOC) was removed from compound **82** using 2 M HCl solution in (CH_3_CH_2_)_2_O^55^ to produce hydrochloride **84**, which was then benzylated with benzyl bromide to produce the final *N*-benzylpiperazine **78** (Scheme 5).

**Scheme 5:**
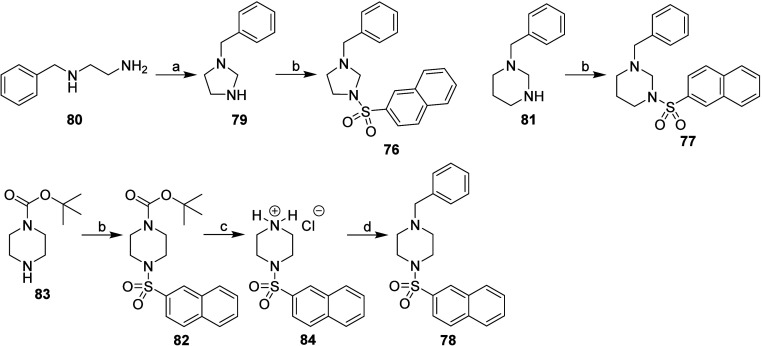
Synthesis of Type D Inhibitors (Imidazolidine, Hexahydropyrimidine, and Piperazine Derivatives)^a^

a Reagents and conditions: (a) (HCHO)_*n*_, 4 Å molecular sieves, dry CH_3_OH, room temperature, under argon, 24 h, 97% crude; (b) naphthalene-2-sulfonyl chloride, (CH_3_CH_2_)_3_N, CH_2_Cl_2_, 0 °C to room temperature, 24 h, 59 to 95%; (c) 2 M HCl solution in (CH_3_CH_2_)_2_O, CH_3_OH, 0 °C to room temperature, 24 h, 97% crude; (d) PhCH_2_Br, Cs_2_CO_3_, acetone, room temperature, 24 h, 85%.

### In Vitro Cholinesterase Inhibitory Activities

In vitro inhibitory potencies against hBChE and hAChE of 23 synthesized type A–D inhibitors was determined using the method of Ellman.^56^ The structures, IC_50_ values and selectivity ratios [SR; i.e., IC_50_ (hAChE)/IC_50_(hBChE)] of type A–D inhibitors and lead compound **2** for comparison are presented in Table 1. Briefly, the IC_50_ values against hBChE range from 10 to 2510 nM, and 3100 to >10^5^ nM against hAChE. Inhibition of AChE is undesired because it causes peripheral (parasympathomimetic) adverse effects.^37–39^

**Table 1:**
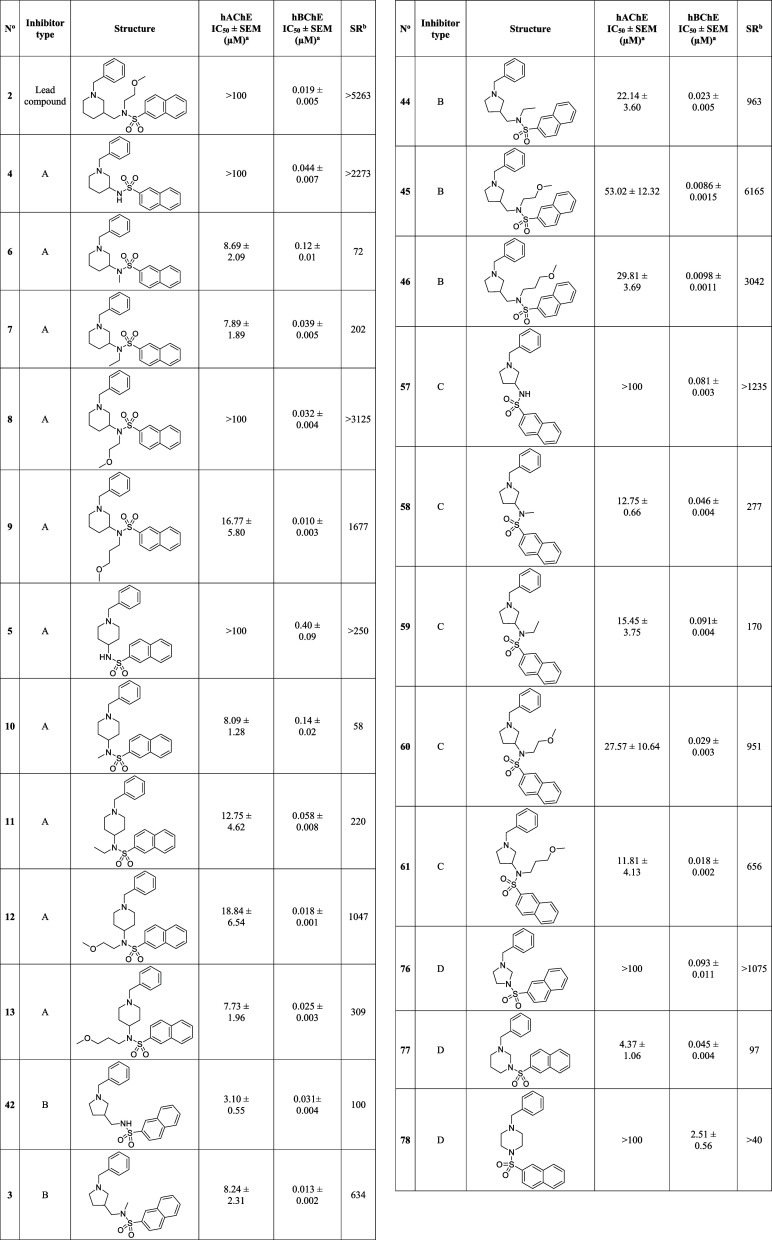
Structures, In Vitro ChE Inhibitory Potencies, and Selectivities toward hBChE of Lead Compound **2** and Type A–D Inhibitors

a The values are expressed as mean ± SEM of at least two independent experiments.

b SR, selectivity ratio: IC_50_(hAChE)/IC_50_(hBChE).

### Structure–Activity Relationships

Compound **9** (IC_50_ = 0.010 μM, Table 1) is the most potent hBChE inhibitor among class I inhibitors. Shortening the alkyl chain on the sulfonamide nitrogen reduces the hBChE inhibitory potency in both 1,3- and 1,4-disubstituted piperidines. 1,3-disubstituted piperidines are more potent hBChE and similarly potent hAChE inhibitors compared to their 1,4-disubstituted analogues. Seven of the 10 class I inhibitors also inhibit hAChE and do so with similar potencies. Their IC_50_ values range from 7.73 to 16.77 μM (Table 1). Compounds **4** (IC_50_ > 100 μM, Table 1) and **5** (IC_50_ > 100 μM, Table 1) show that removing the alkyl chain from the sulfonamide nitrogen abolishes hAChE inhibition.

Compound **45** (IC_50_ = 0.0086 μM, Table 1) is the most potent hBChE inhibitor among class II inhibitors. Shortening or removing the alkyl chain from the sulfonamide nitrogen does not have a profound effect on hBChE inhibition, but it does increase hAChE inhibitory potency. For example, secondary sulfonamide **42** [IC_50_ (hAChE) = 3.10 μM, Table 1] is a 17-fold more potent hAChE inhibitor than tertiary sulfonamide **45** [IC_50_ (hAChE) = 53.02 μM, Table 1].

Class III inhibitors show that (i) reducing the ring size of the six-membered piperidine into the five-membered pyrrolidine does not have a profound effect on hBChE or hAChE inhibition and (ii) removing the methylene group between the pyrrolidine ring and sulfonamide nitrogen decreases hBChE and increases hAChE inhibitory potency. Compound **57** [IC_50_ (hAChE) > 100 μM, Table 1] is the exception, as it is more than 30-fold less potent hAChE inhibitor than its methylene-containing counterpart **42** [IC_50_ (hAChE) = 3.10 μM, Table 1].

Hexahydropyrimidine **77** is the most potent hBChE (IC_50_ = 0.045 μM, Table 1) and hAChE (IC_50_ = 4.37 μM, Table 1) inhibitor among the three class IV inhibitors. Reducing the size of the ring from six-membered hexahydropyrimidine to five-membered imidazolidine (i.e., compound **76**) or replacing it with a piperazine (i.e., compound **78**) reduces the hBChE inhibitory potency (**76**, IC_50_ = 0.093 μM; **78**, IC_50_ = 2.51 μM, Table 1) and abolishes hAChE inhibition (IC_50_ > 100 μM for **76** and **78**).

Pyrrolidine **3** was the most promising compound of the series and was chosen for the next development stage due to its low nanomolar hBChE inhibitory potency (IC_50_ = 13 nM), good selectivity over hAChE (SR = 634) and more favorable properties for increased brain exposure [lower MW, NRB, tPSA^49^ and logarithm of the octanol/water partition coefficient (*C*Log*P*)^57^] compared to lead compound **2** and compounds **45** and **46** (Table 2).

**Table 2:**
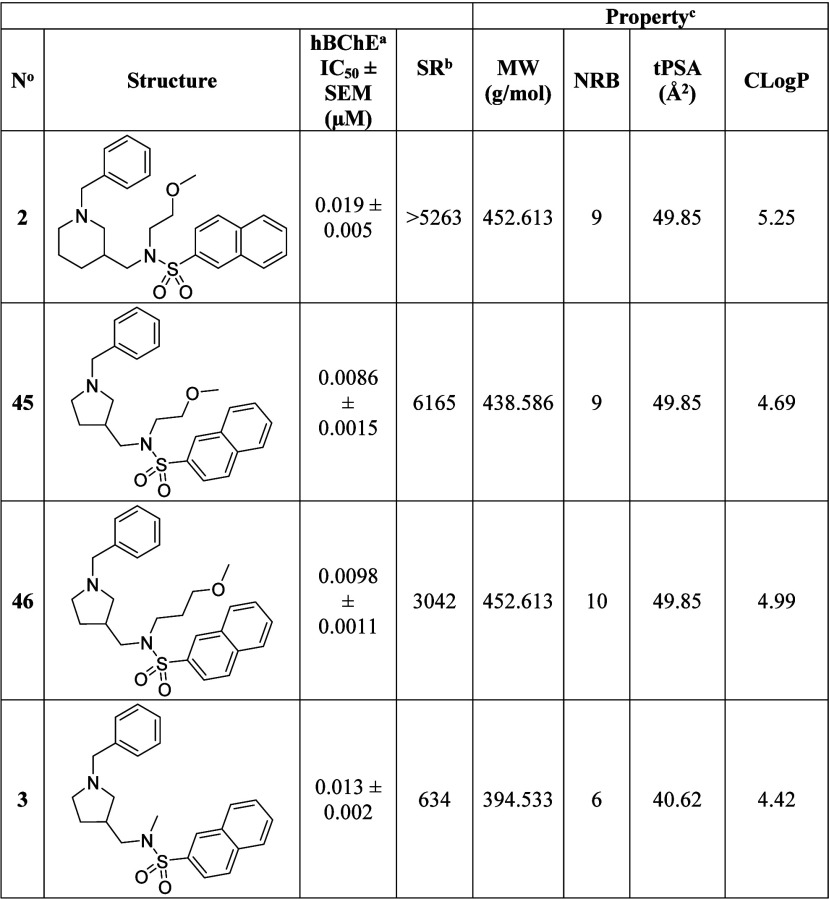
Structures, In Vitro Inhibitory Potencies and Selectivities toward hBChE over hAChE, along with Properties Important for Brain Exposure of Lead Compound **2** and Compounds **45**, **46**, and **3**

a The value is expressed as mean ± SEM of at least two independent experiments.

b SR, selectivity ratio: IC_50_(hAChE)/IC_50_(hBChE).

c Calculated with ChemOffice 2019.

### Synthesis of Compounds **(*S*)-(+)-3** and **(*R*)-(−)-3**

Synthesis of enantiomers of compound **3**, compounds **(** ***S*** **)-(+)-3** and **(** ***R*** **)-(−)-3** is presented in Scheme 6. Compound **(** ***S*** **)-(+)-3** was synthesized from commercially available amine **(** ***R*** **)-86** (abcr, Germany) by first reacting it with naphthalene-2-sulfonyl chloride in the presence of (CH_3_CH_2_)_3_N in CH_2_Cl_2_ ^30^ to produce sulfonamide **87**. Its sulfonamide nitrogen was methylated with methyl iodide in the presence of K_2_CO_3_ in anhydrous DMF at 60 °C to yield compound **88**. The BOC protecting group of compound **88** was removed in CH_3_OH using 2 M HCl solution in (CH_3_CH_2_)_2_O^55^ to produce hydrochloride **89**, which was then benzylated with benzyl bromide in the presence of (CH_3_CH_2_)_3_N in anhydrous CH_2_Cl_2_ to obtain the final *N*-benzylpyrrolidine ***S*** **-(+)-3**. Compound **(** ***R*** **)-(−)-3** was synthesized from commercially available amine **(** ***S*** **)-90** (abcr, Germany) using the same reagents and conditions used for the synthesis of compound **(** ***S*** **)-(+)-3**. The configuration was maintained throughout this synthetic route, even though the Cahn–Ingold–Prelog nomenclature changed in conversions **(** ***R*** **)-86** to **(** ***S*** **)-3** and **(** ***S*** **)-90** to **(** ***R*** **)-91** (Scheme 6).

**Scheme 6:**
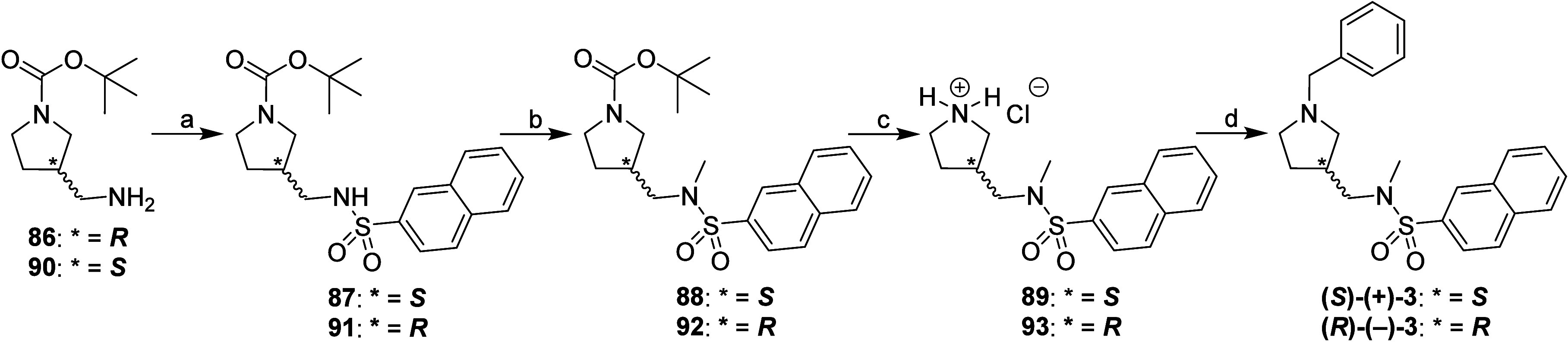
Synthesis of Compounds **(*S*)-(+)-3** and **(*R*)-(−)-3** ^a^

a Reagents and conditions: (a) naphthalene-2-sulfonyl chloride, (CH_3_CH_2_)_3_N, CH_2_Cl_2_, 0 °C to room temperature, 24 h, 99% crude; (b) CH_3_I, K_2_CO_3_, anhydrous DMF, room temperature to 60 °C, under argon, 24 h, 97 to 98% crude; (c) 2 M HCl solution in (CH_3_CH_2_)_2_O, CH_3_OH, 0 °C to room temperature, 24 h, 98 to 99% crude; (d) PhCH_2_Br, (CH_3_CH_2_)_3_N, anhydrous CH_2_Cl_2_, 0 °C to room temperature, 24 h, 65 to 66%.

### Chiral HPLC Analysis of Compounds **3**, **(*S*)-(+)-3**, and **(*R*)-(−)-3**

An analytical chiral HPLC method to determine the enantiomeric purity of synthesized enantiomers **(** ***S*** **)-(+)-3** and **(** ***R*** **)-(−)-3** was developed. Baseline separation of enantiomers of racemic compound **3** (Figure 2A) was achieved on a CHIRALPAK IB-3, 4.6 × 250 mm, 3 μm column with a mixture of *n*-hexane/[(CH_3_)_2_CHOH + 0.1% (CH_3_CH_2_)_3_N] (9/1, v/v) as the mobile phase at a 1 mL/min flow rate. Analysis of synthesized compounds **(** ***S*** **)-(+)-3** (Figure 2B) and **(** ***R*** **)-(−)-3** (Figure 2C) showed both enantiomers were obtained with ee 100%.

**Figure 2:**
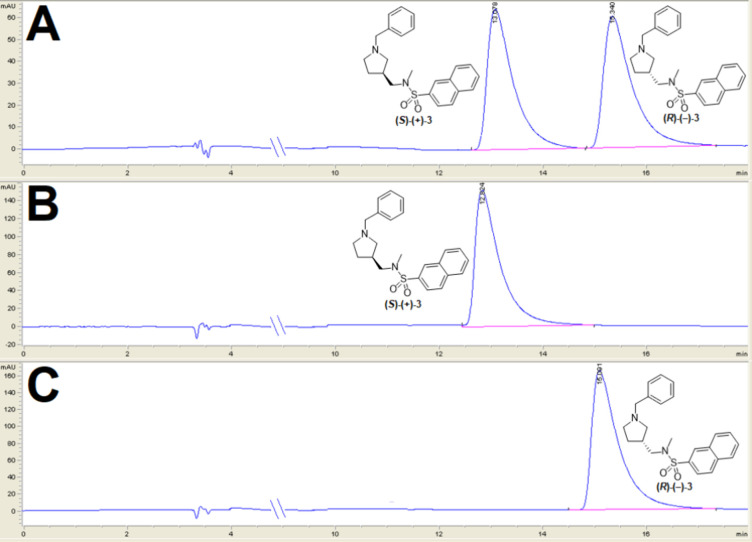
Chiral HPLC analysis of (A) racemic compound **3** [50% **(** ***S*** **)-(+)-3**: *t*_R_ = 13.08 min, 50% **(** ***R*** **)-(−)-3**: *t*_R_ = 15.34 min], (B) compound **(** ***S*** **)-(+)-3** (*t*_R_ = 12.82 min) and (C) compound **(** ***R*** **)-(−)-3 (** *t*_R_ = 15.09 min).

### Absolute Configurations of Compounds **(*S*)-(+)-3** and **(*R*)-(−)-3**

Concentrated CH_2_Cl_2_ solutions of the two enantiomers [**(** ***S*** **)-(+)-3** and **(** ***R*** **)-(−)-3**] were placed in narrow glass test tubes and layered with *n*-hexane at room temperature. Solvents were allowed to slowly diffuse loosely from the sealed tubes and crystals appeared after 3 days. Suitable crystals of each sample were selected (Figure S1) and the absolute configuration of each sample was confirmed by X-ray diffraction experiments (Figure 3). Both compounds crystallized in the achiral space group *P*212121in nearly identical unit cells.

**Figure 3:**
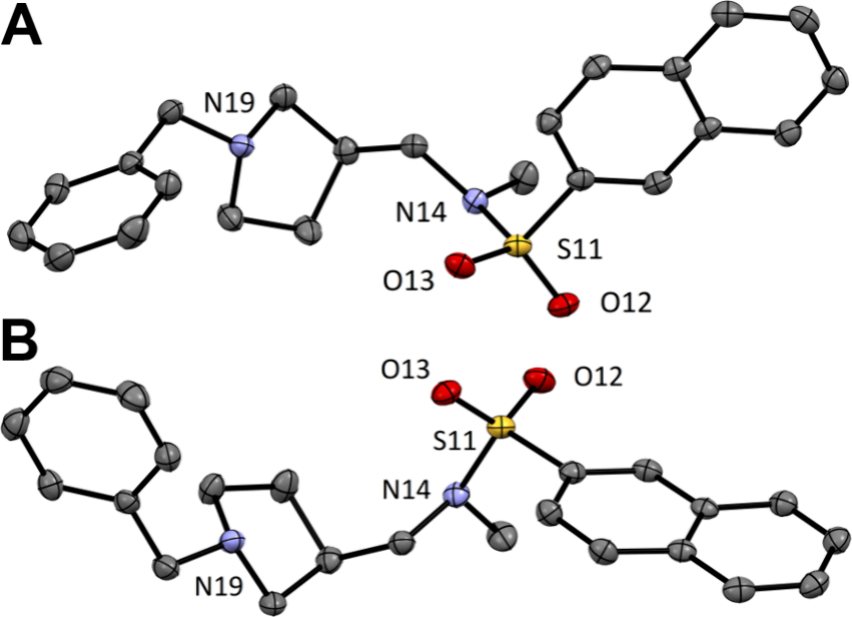
Crystal structures of compounds **(** ***S*** **)-(+)-3** (A) and **(** ***R*** **)-(−)-3** (B) with heteroatom labeling. Thermal ellipsoids are drawn at 50% probability level and hydrogen atoms are omitted for better clarity. Carbon atoms are gray, nitrogen atoms are light purple, oxygen atoms are red and sulfur atoms are yellow.

### In Vitro Cholinesterase Inhibitory Potencies of Compounds **2**, **3**, **(*S*)-(+)-3**, and **(*R*)-(−)-3** and Kinetic Evaluation of Compounds **2**, **(*S*)-(+)-3**, and **(*R*)-(−)-3**

To enable a direct comparison of the in vitro inhibitory potencies of compound **2**, **3**, **(** ***S*** **)-(+)-3** and **(** ***R*** **)-(−)-3** against hBChE and hAChE they were determined in the same assay using the method of Ellman.^56^ Their structures, inhibitory potencies (i.e., IC_50_ values), and SR are presented in Table 3. Results show that compound **(** ***R*** **)-(−)-3** is the eutomer (IC_50_ = 12 nM) and compound ***S*** **-(+)-3** is the distomer (IC_50_ = 56 nM). Compound **(** ***R*** **)-(−)-3** also has higher selectivity over hAChE (SR = 1817) than compound **(** ***S*** **)-(+)-3** (SR = 117).

**Table 3:**
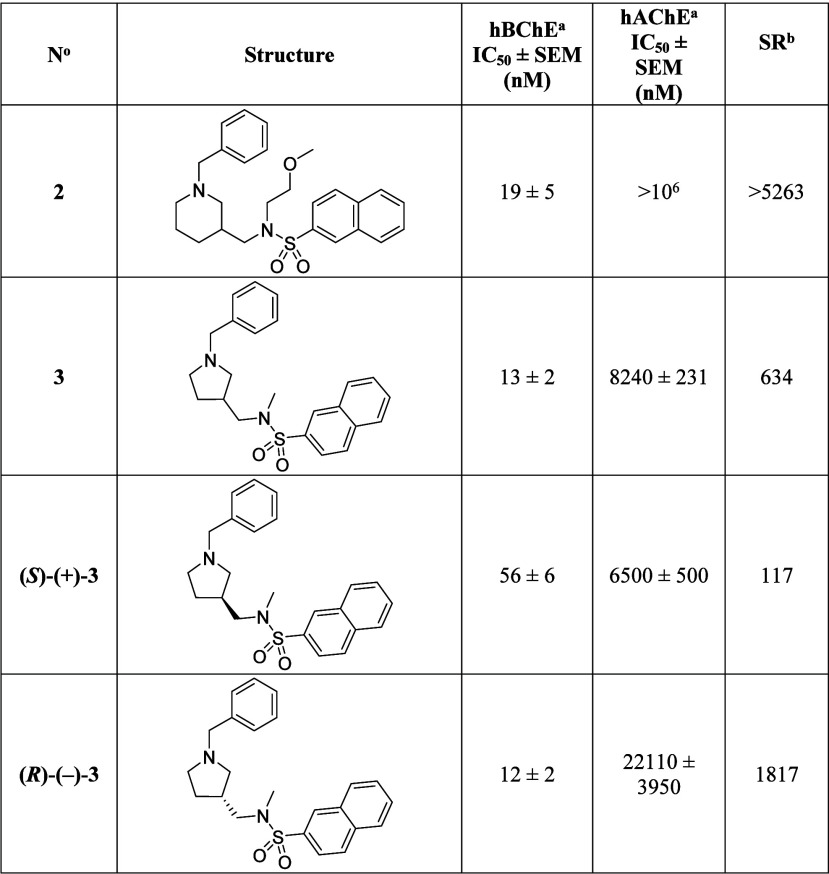
Structures, In Vitro Inhibitory Potencies and Selectivity towards hBChE of Lead Compound **2**, Racemic Compound **3**, and Compounds **(*S*)-(+)-3** and **(*R*)-(−)-3**

a The values are expressed as mean ± SEM of at least two independent experiments.

b SR, selectivity ratio: IC_50_(hAChE)/IC_50_(hBChE).

To determine the accurate affinities of compounds **2**, **(** ***S*** **)-(+)-3** and **(** ***R*** **)-(−)-3** toward hBChE and enable their direct comparison, the compounds were evaluated in the same enzyme kinetic experiments performed on a stopped-flow apparatus in line with Copland’s guidelines.^58^ The progress curves of the enzymatic reaction product formation were monitored in the presence and absence of each **(** ***S*** **)-(+)-3** and **(** ***R*** **)-(−)-3** (Figure 4A,B) and analyzed the measured progress curves with the ENZO software.^59^ This program is able to evaluate model specific kinetic parameters by fitting the parameters of the corresponding differential equations to the experimental curves. In the starting stage of the reaction no curvature was observed (Figure 4A,B). This shows that **(** ***S*** **)-(+)-3** and **(** ***R*** **)-(−)-3** are fast binding hBChE inhibitors. Among several reaction schemes tested, the one, allowing for the inhibitor to react with the free hBChE and with hBChE-substrate complex fitted the experimental data (Figure 4C) and was also in line with crystallographic findings (see below).

**Figure 4:**
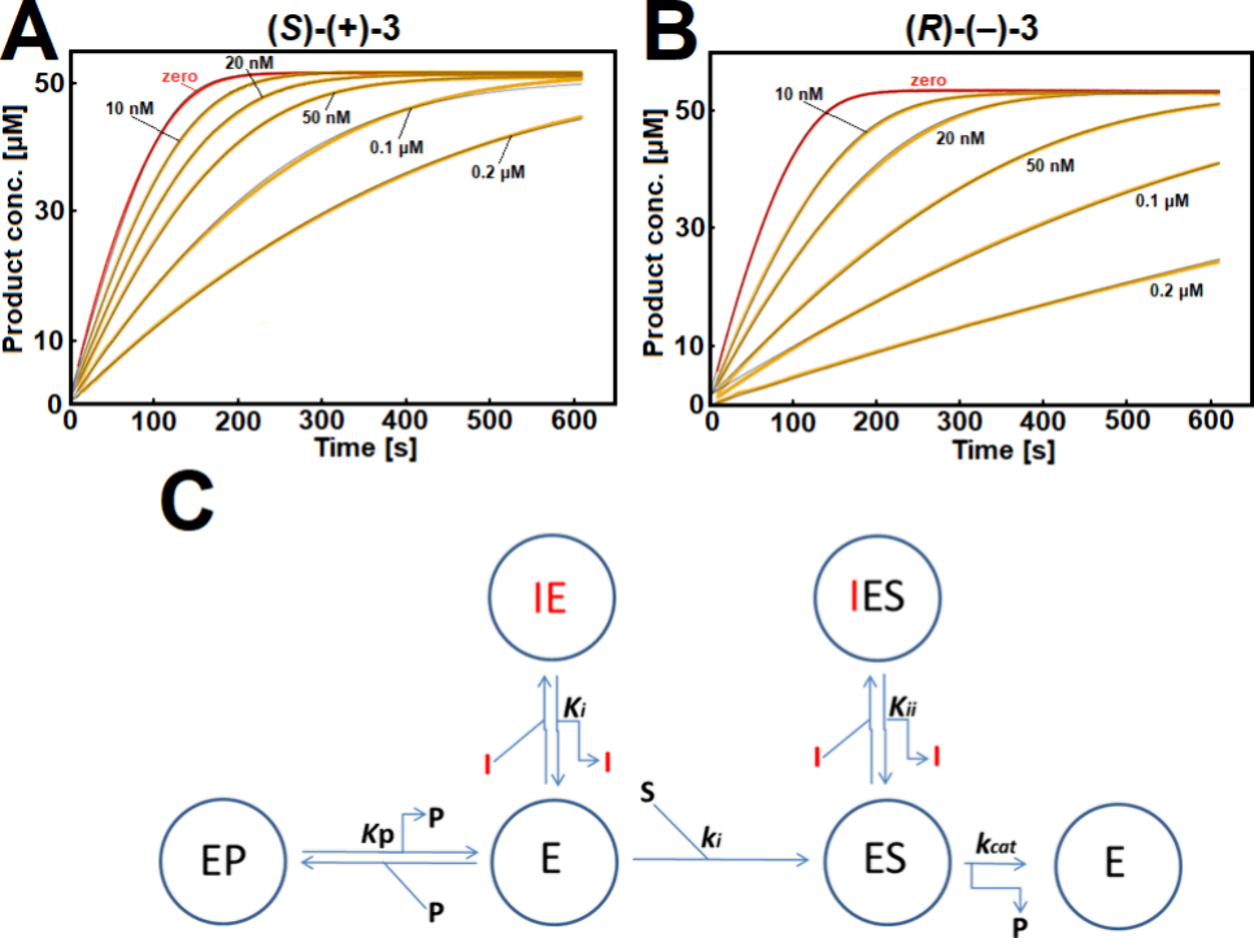
Kinetic evaluation of **(** ***S*** **)-(+)-3** and **(** ***R*** **)-(−)-3**. Progress curves for hydrolysis of 50 μM butyrylthiocholine (BTCh) by 0.5 nM hBChE in the absence (zero; red curve) and presence of (A) compound **(*S*)-(+)-3** or (B) compound **(*R*)-(−)-3** at 10 nM, 20 nM, 50 nM, 0.1 μM and 0.2 μM (orange curves). The data was obtained using a UV/vis spectrophotometer and each concentration was performed in duplicate. (C) Competitive single-step inhibition mechanism with fast association for each pure enantiomer fully reproduced the progress curves obtained. E, enzyme; I, inhibitor; P, product [thiocholine-thionitro benzoate (TCh-TNB^–^) product–the mixed anhydride generated from Ellman’s reagent and thiocholin];^60^ S, substrate; *k*_cat_, *k*_i_, *K*_i_, *K*_ii_ and *K*_p_, kinetic constants.

The inhibition constants (*K*_i_) on purified, recombinant hBChE are 10.2, 14.9, and 6.6 nM, for compounds **2**, **(** ***S*** **)-(+)-3** and **(** ***R*** **)-(−)-3**, respectively. A higher degree of stereoselective inhibition of full plasma hBChE was observed with inhibition constant 52.0 nM for compound **(** ***S*** **)-(+)-3** and 12.1 nM for compound **(** ***R*** **)-(−)-3**. This suggests that compound **(** ***S*** **)-(+)-3** has higher plasma protein binding than compound **(** ***R*** **)-(−)-3**. The inhibition constant of compound **2** on full plasma hBChE is 22.7 nM (Table 4). Cleary, compound **(** ***R*** **)-(−)-3** is the eutomer, has higher selectivity toward hBChE than compound **(** ***S*** **)-(+)-3** and is a more potent hBChE inhibitor than lead compound **2**. However, **(** ***R*** **)-(−)-3** (*K*_i_ = 6.6 nM, Table 4) is a less potent hBChE inhibitor than the enantiomers of racemate **2**: **(+)-2** (*K*_i_ = 2.01 nM) and **(−)-2** (*K*_i_ = 1.29 nM).^30^ Nevertheless, in our in vitro (see above) and in vivo (see below) pharmacological evaluation of **(** ***R*** **)-(−)-3**, this compound was compared directly to racemic compound **2** because compound **2** was previously evaluated in vivo as a racemate and not as a pure enantiomer.^30,33^

**Table 4:**
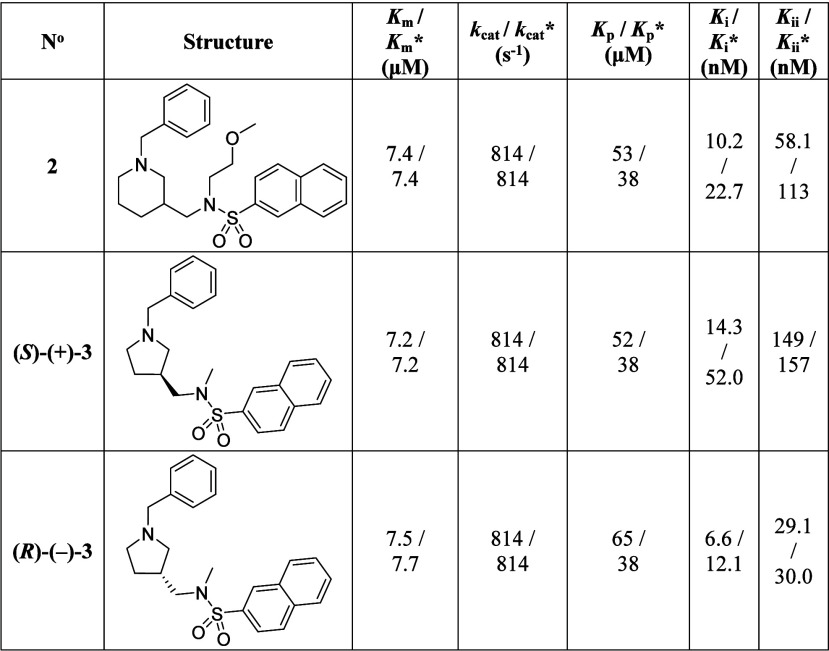
Characteristic Kinetic Parameters for the Interaction of Compounds **2**, **(*S*)-(+)-3**, and **(*R*)-(−)-3** with Purified hBChE and with Full Plasma hBChE (Noted with “*” Above the Constant), Determined in the Presence of 50 μM Butyrylthiocholine^a^

a *K*_m_, Michaelis constant; *k*_cat_, turnover number of hBChE; *K*_p_, product (TCh-TNB^–^) inhibition constant; *K*_i_, inhibition constant for binding to free hBChE; *K*_ii_, inhibition constant for binding to hBChE-substrate complex (Figure 4C).

### X-ray Crystal Structures of hBChE in Complex with Enantiomers **(*S*)-(+)-3** and **(*R*)-(−)-3**

Structures of compounds **(** ***S*** **)-(+)-3** and **(** ***R*** **)-(−)-3** bound to hBChE were determined by X-ray crystallography after protein–ligand complexes formation by crystal soaking. Both enantiomers “bind” deep in the gorge of hBChE, over the active site of the enzyme and thus prevent substrate access through steric hindrance. The naphthalene ring of both enantiomers’ forms π–π interactions with aromatic residues Trp231, Phe329 and Phe398. This is characterized by naphthalene distal-ring/residue-ring respective distances of 4.6, 5.2, and 6.1 Å for compound **(** ***S*** **)-(+)-3** and 4.6, 5.3, and 5.7 Å for compound **(** ***R*** **)-(−)-3**. Similarly, the *N*-benzyl ring of both enantiomers’ forms π–π interactions with Trp82 with a distance of 4.1 Å to the pyrrole ring. The sulfonamide oxygens of both compounds form tight hydrogen bonds with amino acid residues. One oxygen forms a hydrogen bond with the Thr120 side chain, with sulfonamide oxygen/Thr120-Oγ distances of 3.3 Å for compound **(** ***S*** **)-(+)-3** and 3.0 Å for compound **3**, while the second oxygen interacts through a hydrogen bond with a molecule of water bridged to the main chain carbonyl oxygen of Asn68. Compound **(** ***S*** **)-(+)-3** forms a somewhat looser interaction characterized by a water-sulfonyl oxygen distance of 3.9 Å compared to 3.3 Å for compound **(** ***R*** **)-(−)-3**. This could explain the fact that **(** ***R*** **)-(−)-3 (** *K*_i_ = 6.6 nM, Table 4) is a more potent hBChE inhibitor than **(** ***S*** **)-(+)-3** (*K*_i_ = 14.3 nM, Table 4). Finally, both enantiomers orient their pyrrolidine nitrogen’s toward Asp70/Tyr332, which are part of the so-called peripheral binding site of hBChE. Compound ***S*** **-(+)-3** gets closer to Tyr332 with pyrrolidine nitrogen/Tyr332-OH and pyrrolidine nitrogen/Asp70-Oδ respective distances of 3.5 and 3.9 Å, while compound **(** ***R*** **)-(−)-3**, due to its opposite stereochemical configuration, “flips” its pyrrolidine ring and orients closer to Asp70 with distances of 3.4 Å for the respective similar interactions (Figure 5).

**Figure 5:**
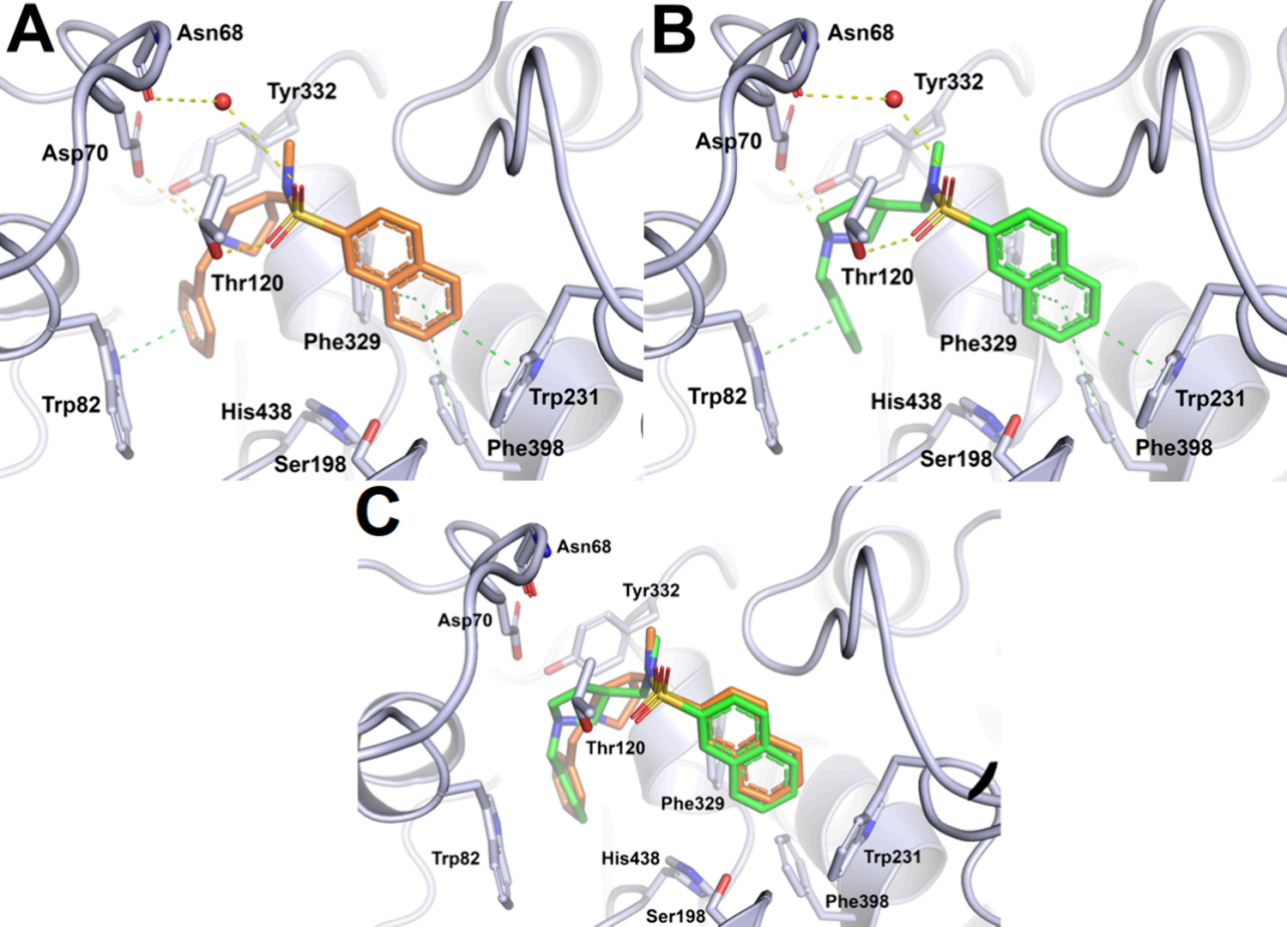
(A) X-ray structure of compound **(** ***S*** **)-(+)-3** (orange sticks) bound to hBChE, (B) X-ray structure of compound **(** ***R*** **)-(−)-3** (green sticks) bound to hBChE, and (C) superposition of both compounds **(** ***S*** **)-(+)-3** (orange sticks) and **(** ***R*** **)-(−)-3** (green sticks) bound to hBChE. Compounds **(** ***S*** **)-(+)-3**, **(** ***R*** **)-(−)-3** and amino acid residues of hBChE that form interactions are presented as sticks: carbon atoms in orange, green or gray, nitrogen, oxygen and sulfur atoms in blue, red and yellow, respectively. Water molecule is depicted as a red sphere, π–π interactions as green dashed lines and hydrogen bonds as yellow dashed lines.

### Cytotoxicity Neuroprotective Activity of Compounds **2**, **(*S*)-(+)-3**, and **(*R*)-(−)-3**

HepG2 and SH-SY5Y cells were treated with increasing concentrations (0, 1, 5, 10, 20, 50, and 100 μM) of compounds **2**, **(** ***S*** **)-(+)-3** and **(** ***R*** **)-(−)-3** and the metabolic activity of cells was assessed with the MTS assay after 48 h. Results show that compounds **2**, **(** ***S*** **)-(+)-3** and **(** ***R*** **)-(−)-3** have comparable cytotoxicity profiles in both HepG2 (Figure 6A) and SH-SY5Y (Figure 6B).

**Figure 6:**
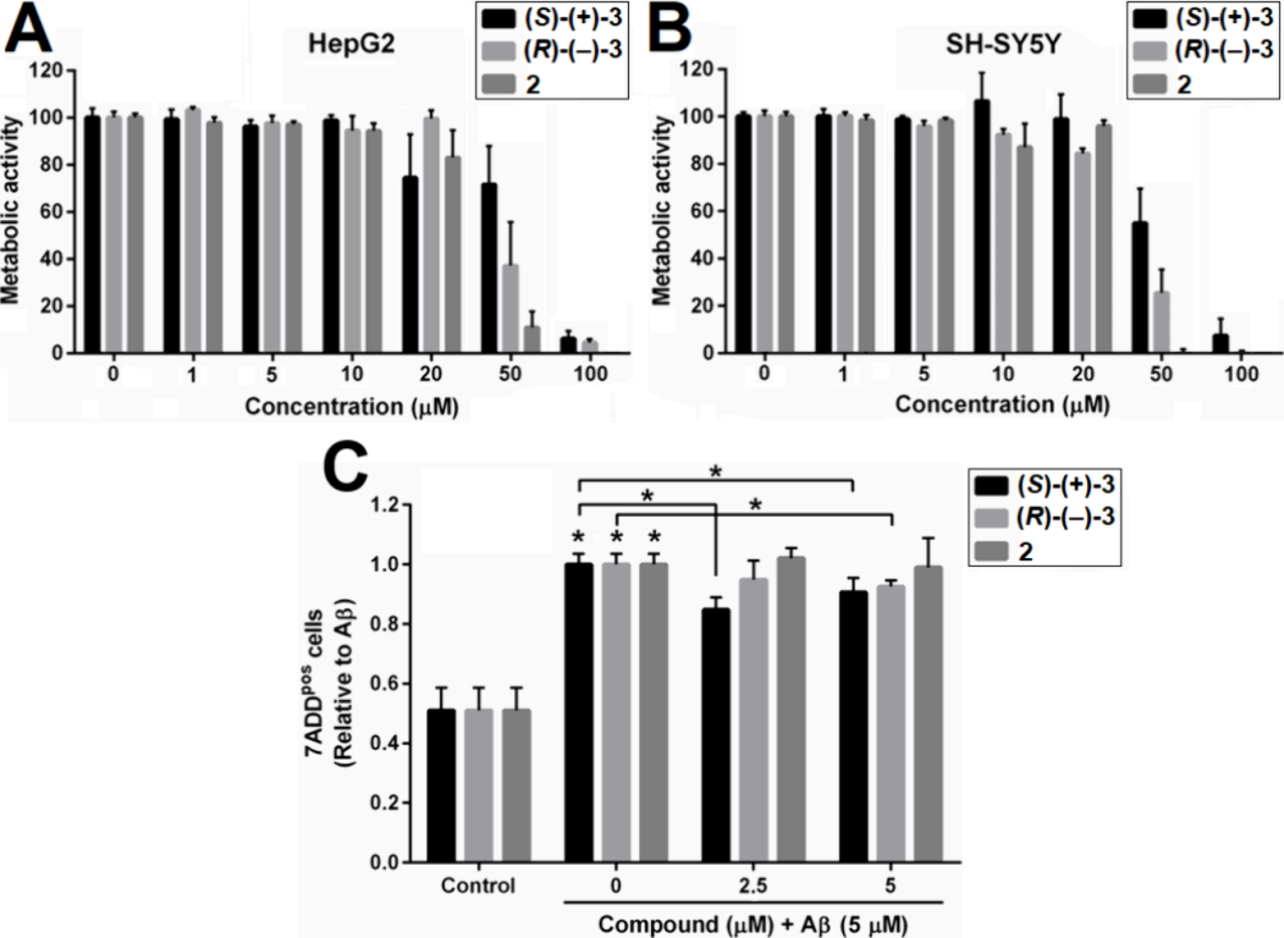
(A, B) Impairment of cell metabolic activity (relative to control) by compounds **2**, **(** ***S*** **)-(+)-3** and **(** ***R*** **)-(−)-3**. (A) HepG2 and (B) SH-SY5Y cells were treated in the presence of increasing concentrations (0, 1, 5, 10, 20, 50, and 100 μM) of compounds **2**, **(*S*)-(+)-3** and **(*R*)-(−)-3** in reduced-serum medium. The metabolic activity was determined after 48 h using the MTS assay. 0 μM of compound was considered as 100% cell viability and the data is presented as means (±SEM) of three independent experiments, each performed in quadruplicate. (C) Neuroprotective activity of compounds **2**, **(*S*)-(+)-3** and **(*R*)-(−)-3** against Aβ_1–42_-induced toxicity. SH-SY5Y cells were treated with preaggregated Aβ_1–42_ (5 μM) in the absence or presence of compounds **2**, **(*S*)-(+)-3** and **(*R*)-(−)-3** (2.5 and 5 μM). The neuroprotective effect was evaluated after 48 h by flow cytometry analysis of 7AAD staining. The chart shows the fraction of 7AAD positive cells (7AADpos) which indicates the percentage of dead cells. DMSO treated cells were considered as control cells. Cells were treated in duplicate. The values are the mean ± SEM of two independent experiments (one-way ANOVA, *t* test; *p < 0.05, ** *p* < 0.1).

To determine if compounds **2**, **(** ***S*** **)-(+)-3** and **(** ***R*** **)-(−)-3** have neuroprotective activity, SH-SY5Y cells were treated for 48 h with 5 μM of preaggregated Aβ_1–42_ in the presence or absence of **2**, **(** ***S*** **)-(+)-3** and **(** ***R*** **)-(−)-3** at concentrations 2.5 and 5 μM. The increased number of 7-AAD positive cells show that treatment with preaggregated Aβ_1–42_ only (i.e., absence of our compounds) resulted in increased cell death when compared to control cells. Compounds **(** ***S*** **)-(+)-3** (at 2.5 and 5 μM) and **(** ***R*** **)-(−)-3** (at 5 μM) have neuroprotective activity as they diminished Aβ_1–42_-induced cell death. Compound **2** does not have neuroprotective activity at either concentration (Figure 6C).

### Solubility of Compounds **2** HCl and **(*R*)-(−)-3** HCl in Various Aqueous Media

Compound **(** ***R*** **)-(−)-3** was chosen over compound **(** ***S*** **)-(+)-3** for further biological evaluation because it is the more potent and more selective hBChE inhibitor. Compound **(** ***R*** **)-(−)-3** was converted into its hydrochloride salt [**(** ***R*** **)-(−)-3 HCl**] in CH_3_OH using 2 M solution of HCl in (CH_3_CH_2_)_2_O and measured its solubility and the solubility of hydrochloride of compound **2** (**2 HCl**) in six aqueous media using a small volume shake flask technique. The results are presented in Table 5 in mM for easier comparison. The solubility of compound **(** ***R*** **)-(−)-3 HCl** compared to compound **2 HCl** is lower in all aqueous media examined (Table 5). This was an expected outcome of the structural modifications aimed to increase the lipophilicity needed for improved brain exposure.

**Table 5:**
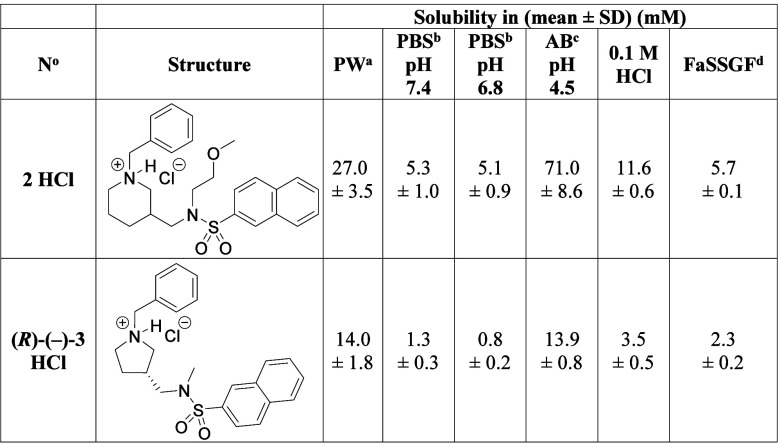
Solubilities of Compounds **2** HCl and **(*R*)-(−)-3** HCl in Various Aqueous Media

a PW, purified water.

b PBS, phosphate-buffered saline.

c AB, acetate buffer.

d FaSSGF, fasted state simulated gastric fluid.

### Permeability of Compounds **2** and **(*R*)-(−)-3** in Caco-2 Cells

Simple in vitro bidirectional permeability measurements in Caco-2 (heterogeneous human epithelial colorectal adenocarcinoma) cells are commonly used in preclinical drug research to predict absorption of compounds in humans^60^ because permeability values (*P*_app_) in these cells correlate well with in vivo permeation.^49^ Since Caco-2 cells express membrane transport proteins like breast cancer resistance protein (BCRP) or P-glycoprotein,^61,62^ which actively efflux drugs, these cells are also used to investigate a compound’s susceptibility to transport mechanisms.^49^

The *P*_app_ of compounds **2** and **(** *R* **)-(−)-3** in both the absorptive [apical-to-basolateral (A–B); *P*_app(A–B)_] and eliminatory [basolateral-to-apical (B–A); *P*_app(B–A)_] directions were determined and their efflux ratios (ER) [*P*_app(B–A)_/*P*_app(A–B)_] calculated (Table 6).

**Table 6:**
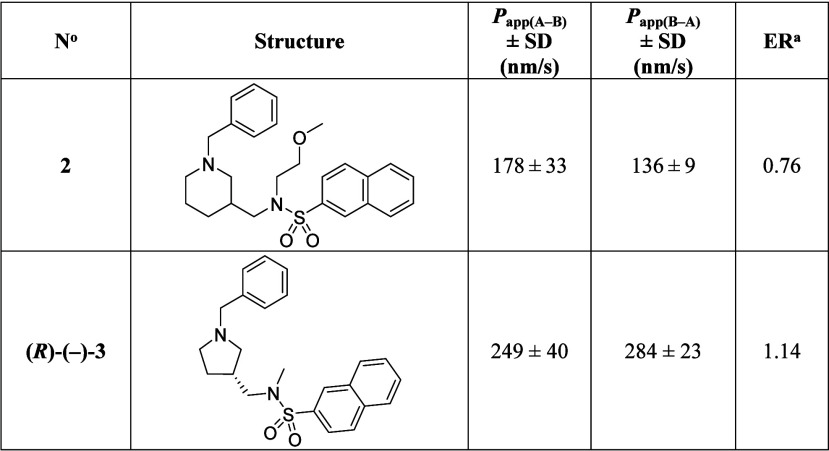
Permeability Values (*P*_app_) and the Efflux Ratios of Compounds **2** and **(*R*)-(−)-3** in Caco-2 Cells

a ER, Efflux ratio **=** *P*_app(B–A)_/*P*_app(A–B)_.

The *P*_app(A–B)_ values are 187 and 249 nm/s for compounds **2** and **(** *R* **)-(−)-3**, respectively (Table 6). These values classify the in vitro permeabilities of both compounds as “high” according to the biopharmaceutical classification system, meaning that the rate of intestinal absorption would not limit the oral bioavailability of these compounds.^63^ No significant difference between *P*_app_ measured in the two directions (expressed as ER) indicates no active transport or efflux^63,64^ for compound **2** (ER = 0.76; Table 6) or **(** *R* **)-(−)-3** (ER = 1.14; Table 6).

### In Vivo Brain–Plasma Distribution of Compounds **2** and **(*R*)-(−)-3**

Brain exposure is imperative for drugs designed to treat brain diseases like AD.^49^ The distributions of compounds **2** and **(** ***R*** **)-(−)-3** [used in the assay as **2 HCl** and **(** ***R*** **)-(−)-3 HCl**] between the brain and the plasma of 3-month-old female and male Balb/c mice were measured and compared using liquid chromatography combined with tandem mass spectrometry (LC-MS/MS) for the analysis. One hour after the mice were given via oral gavage tap water solutions of compounds **2 HCl** and **(** ***R*** **)-(−)-3 HCl** (10 mg/kg dose) or donepezil hydrochloride (positive control; 2 mg/kg dose) or tap water (negative control), they were euthanized. Blood samples were collected and brain tissue isolated. The method for preparation of plasma and brain samples for LC-MS/MS quantification includes homogenization, centrifugation, and solid-phase extraction. The following brain-to-plasma ratios were calculated: 1.3 ± 0.4 for compound **2**, 9.0 ± 1.6 for compound **(** ***R*** **)-(−)-3** and 5.4 ± 1.0 for donepezil (lit.^65^ 6.3) Therefore, our structural modifications of lead compound **2** produced a hBChE inhibitor [compound **(** ***R*** **)-(−)-3**] that is not only more potent, but also 7-fold more brain permeable.

### Activity of Compound **(*R*)-(−)-3** in Mice with Scopolamine-Induced Cognitive Deficits

For the assessment of potential procognitive effects of compound **(** ***R*** **)-(−)-3** [used as **(** ***R*** **)-(−)-3 HCl** in all in vivo assays] two behavioral assays were performed, the passive avoidance (PA) task and novel object recognition (NOR) task. These assays were selected because they are complementary to each other in terms of differential stimuli used and various types of memory functions they can assess. Both of these tasks enable the evaluation of a compounds’ effect on animals’ learning skills, memory acquisition and memory retention.^66^

A nonselective muscarinic ACh receptor (mAChR) antagonist scopolamine [used as a hydrobromide salt in 1 mg/kg dose injected subcutaneously (s.c.)] was used to induce cognitive deficits in adult male Albino Swiss (CD-1) mice in the PA and NOR tasks. Scopolamine was administrated 30 min prior to the acquisition (training) trials. In experimental animals, scopolamine produces cognitive deficits resembling those caused by the cholinergic hypofunction in AD.^67–70^ ChE (i.e., AChE and/or BChE) inhibitors prevent the ChE (i.e., AChE and/or BChE) catalyzed hydrolysis/deactivation of ACh and thus increase its synaptic levels (notably in the brain) which then displaces scopolamine from mAChR and results in the alleviation of scopolamine-induced cognitive deficits.^69,71^ Scopolamine-induced amnesia is therefor frequently used for assessment of procognitive effects of ChE inhibitors (i.e., AChE and/or BChE) in preclinical AD research.^67,72–75^

The PA task is a fear-motivated task that uses an aversive stimulus (i.e., a foot shock) to assess long-term contextual memory based on negative reinforcement. It is one of the most abundantly used behavioral assays for measuring procognitive effects of drug candidates in rodents. The PA task is very powerful when used to test a compounds’ effect on memory consolidation. Since it is based on single-trial learning and requires no pretraining, it is a high-throughput behavioral assay, which explains its continued popularity in behavioral pharmacology.^76^

In the PA task memory performance is positively correlated with the latency to escape from the white compartment of the device and the better the recollection, the longer the latency. In our study, results show a prolongation of the retention trial step-through latency compared to that of the acquisition trial in vehicle-treated mice (i.e., not treated with scopolamine). This indicates unimpaired learning abilities and memory of these animals. The comparison of the retention trial step-through latency of vehicle-treated mice and the retention trial latency of scopolamine-treated mice control reveals memory and learning deficits in the latter group. In contrast to this, in the retention trial, mice treated with scopolamine and compound **(** ***R*** **)-(−)-3** [10 mg/kg injected intraperitoneally (i.p.)] demonstrate prolonged step-through latency compared to mice treated with scopolamine only (Figure 7A). This clearly shows that compound **(** ***R*** **)-(−)-3** improves scopolamine-induced contextual memory deficits. At the dose 1 mg/kg compound **(** ***R*** **)-(−)-3** (injected i.p.) is not effective in this assay (Figure 7A).

**Figure 7:**
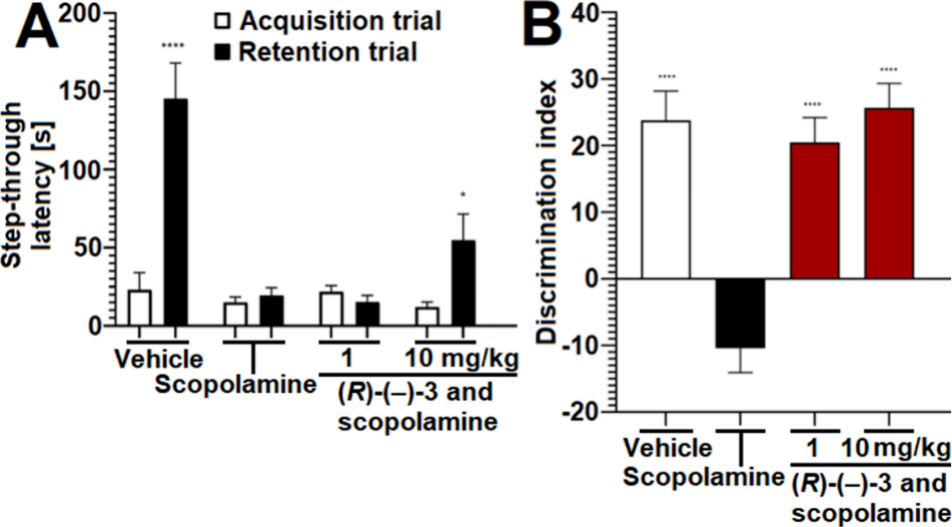
Effects of compound **(** ***R*** **)-(−)-3** on cognitive functions of mice with scopolamine-induced cognitive deficits using the PA and NOR tasks. (A) Effect of compound **(** ***R*** **)-(−)-3** (1 and 10 mg/kg, injected i.p.) on fear-motivated contextual learning and memory in the PA task. The results are presented as mean step-through latency (±SEM) assessed in the acquisition trial and 24 h later (in the retention trial) for *n* = 8–10. Statistical analysis: repeated measures ANOVA followed by Dunnett’s *post hoc* comparison. Significance vs scopolamine-treated mice in the retention trial: * *p* < 0.05 and **** *p* < 0.0001. (B) Effects of compound **(** ***R*** **)-(−)-3** (1 and 10 mg/kg, injected i.p.) in the NOR task. Scopolamine (1 mg/kg, injected s.c.) was administrated 30 min before the trial and compound **(** ***R*** **)-(−)-3** (injected i.p.) 60 min before the trial. The data is shown as means (±SEM) for *n* = 8–10. Statistical analysis: one-way ANOVA followed by Dunnett’s *post hoc* comparison. Significance vs scopolamine-treated mice: * *p* < 0.05, **** *p* < 0.0001.

The NOR task is a behavioral assay for testing the neurobiology of nonspatial memory in rodents. In contrast to the PA task, the NOR task is based on the natural tendency of rodents to explore novel objects and the preference to explore the novel object in the absence of external reinforcements. This behavior reflects on learning abilities and recognition memory. This behavioral assay measures working memory, attention and preference for novelty.^77–79^

In the NOR task, discrimination index (DI) reflects on the preference of mice to explore a novel or a familiar object; the higher the DI the stronger the preference for the novel object. In our study scopolamine induces memory deficits in mice as it decreases the DI. Mice treated with scopolamine and compound **(** ***R*** **)-(−)-3** (1 and 10 mg/kg, injected i.p.) exhibit preference for the novel object as shown by the increased DI (Figure 7B). This shows that compound **(** ***R*** **)-(−)-3** reverses scopolamine-induced recognition memory deficits.

### Activity of Compound **(*R*)-(−)-3** in Mice with Aβ_1–42_-Induced Cognitive Deficits

Encouraged by the procognitive effects of compound **(** ***R*** **)-(−)-3** in mice with scopolamine-induced cognitive deficits (Figure 7), the effects of rivastigmine and compounds **2** and **(** ***R*** **)-(−)-3** were directly compared in mice with Aβ_1–42_-induced cognitive deficits using the Morris water maze (MWM) task. In mice, intracerebroventricular (i.c.v.) injection of Aβ_1–42_ oligomers solution substantially abolishes their information processing which leads to memory impairment and acutely induces AD-like cognitive deficits.^80,81^ Mice treated with Aβ not only show AD-like behavioral abnormalities but they can also exhibit Aβ pathology, the common feature shared by familial and sporadic AD.^81^ ICR mice were used for this behavioral assay.

The MWM task is a hippocampus-dependent spatial learning and memory task used to evaluate a compound’s (i) effect on working and long-term memory by measuring the time needed to reach the escape platform (i.e., escape time latency) during the acquisition (i.e., spatial learning) phase, (ii) effect on cognition and memory (assessed in the retention, drug-off trial) by measuring the total distance swam to get to the escape platform and (iii) production of sensorimotor insufficiencies by measuring the mean speed of swimming.^82–84^ In the retention trial (day 6 of the MWM task) mice treated with Aβ_1–42_ oligomers (10 μg per mouse, injected i.c.v.) showed impaired learning abilities and cognitive functions compared to mice injected with saline and sham-operated mice, as they traveled longer distances to reach the target zone (Figure 8A) and had longer escape latencies (Figure 8B). Mice treated with Aβ_1–42_ oligomers and rivastigmine (1 mg/kg, injected i.p.) and mice treated with Aβ_1–42_ oligomers and compound **(** ***R*** **)-(−)-3** (10 mg/kg, injected i.p.) showed improved learning abilities and cognitive functions compared to mice treated with Aβ_1–42_ oligomers (Figure 8). Mice treated with Aβ_1–42_ oligomers and rivastigmine and mice treated with Aβ_1–42_ oligomers and compound **(** ***R*** **)-(−)-3** traveled shorter distances to reach the target zone (i.e., the former platform location) (Figure 8A) and had shorter escape latencies compared to mice treated with Aβ_1–42_ (Figure 8B). This clearly shows that compound **(** ***R*** **)-(−)-3** improves Aβ_1–42_-induced cognitive deficits and is superior at this, as compared to lead compound **2**, which was not effective in this disease model (Figure 8).

**Figure 8:**
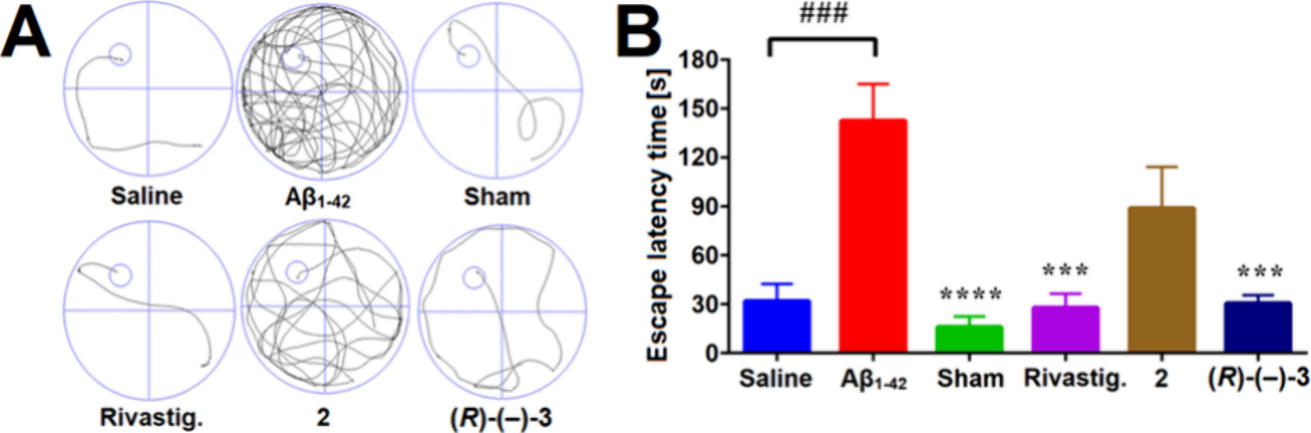
Comparison of effects of rivastigmine (Rivastig.) (1 mg/kg, injected i.p.) and compounds **2** (10 mg/kg, injected i.p.) and **(** ***R*** **)-(−)-3** (10 mg/kg, injected i.p.) on cognitive functions of mice with Aβ_1–42_-induced cognitive deficits. (A) Representative swimming path of a mouse to the target zone (i.e., the former platform location) recorded on day 6 of the MWM task (retention phase). (B) Latencies to reach the target zone measured on day 6 of the MWM task. Statistical analysis: one-way ANOVA followed by Tukey’s *post hoc* comparison; significance: ^###^ *p* < 0.001 vs saline-treated (control) mice; *** *p* < 0.001, **** *p* < 0.0001 vs Aβ_1–42_ treated mice.

### Peripheral Adverse Effects and Acute Toxicity of Compound **(*R*)-(−)-3**

Undesired peripheral side effects of anti-AD ChE inhibitors (i.e., donepezil, rivastigmine and galantamine) are caused by the excess ACh, which is the result of AChE inhibition in the parasympathetic autonomic nervous system, basal ganglia and the peripheral nervous system.^85^ This manifests in rodents as salivation, diarrhea and depressed locomotor activity.^86,87^ These effects are not expected for selective BChE inhibition, since BChE inhibition does not cause peripheral adverse effects.^27–36^ As expected, compound **(** ***R*** **)-(−)-3** does not cause salivation or diarrhea in CD-1 mice at any of the doses tested in the PA and NOR tasks (1 and 10 mg/kg, injected i.p.), at a higher dose (30 mg/kg, injected i.p.) and also in adult female Balb/c mice at doses 10 and 100 mg/kg (administrated orally as solutions in tap water).

After the toxicity test with 100 mg/kg **(** ***R*** **)-(−)-3** (administrated orally as a solution in tap water) in adult female Balb/c mice, the animals were euthanized and a complete necropsy was performed immediately. Apart from one mouse (No. 3 in Table S1) with slightly dilated and congested uterine horns, consistent with the diagnosis of cystic endometrial hyperplasia, the necropsy showed no gross lesions. Samples of stomach, small intestine (jejunum), cecum, colon, liver, kidney, adrenal gland, brain, spleen, lung and heart were collected and a histopathologic examination was performed. In this examination no histopathological lesions were found that would indicate toxic effects of compound **(** ***R*** **)-(−)-3** at the dose 100 mg/kg (Table S1).

### Effects of Compound **(*R*)-(−)-3** on Mice Motor Performance

The rotarod test is a behavioral assay for evaluation of the effects of drug candidates on motor performance of rodents. In this assay, the time a rodent spends on the rotating rotarod is measured and is a direct reflection of the animals’ balance coordination, endurance, motor planning and physical condition.^88^ At three different rotations speeds [6, 18, and 24 rotations per minute (rpm)] in the rotarod test, mice treated with compound **(** ***R*** **)-(−)-3** (10 mg/kg, injected i.p.) showed no motor deficits compared to mice treated with the vehicle only (Figure 9A–C). Therefore, just like lead compound **2**,^30^ optimized lead compound **(** ***R*** **)-(−)-3** does not produce any motor deficits in mice.

**Figure 9:**
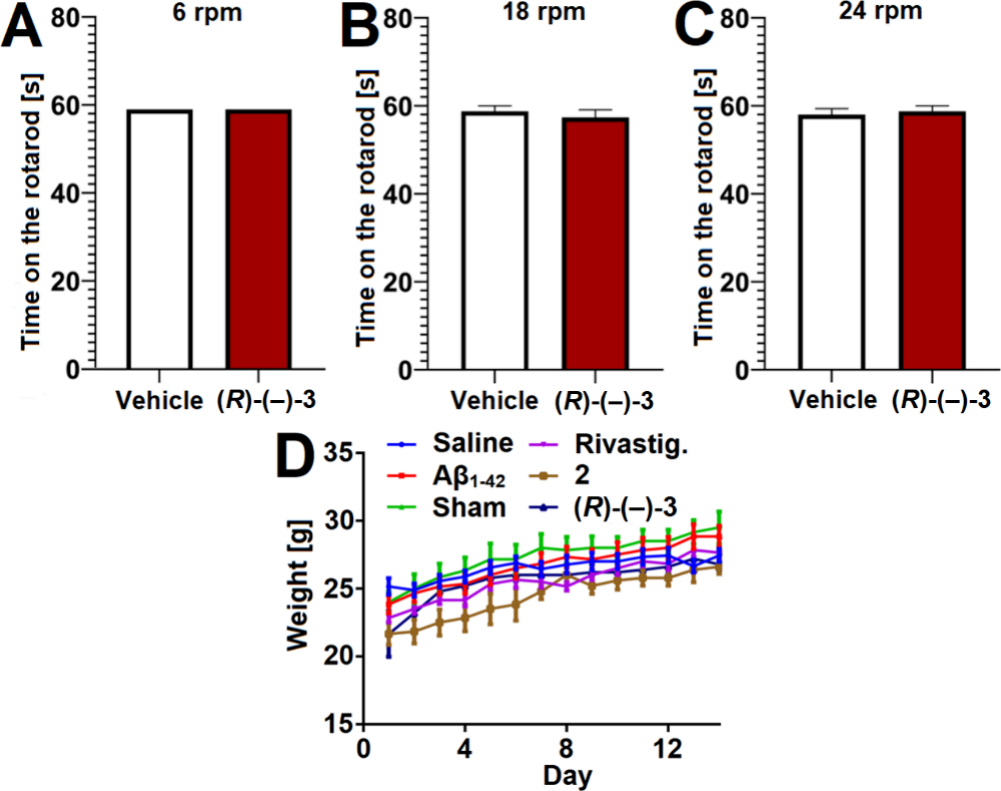
(A, B and C) Effect of the compound **(** ***R*** **)-(−)-3** (10 mg/kg, injected i.p.) on motor coordination of mice measured in the rotarod test. The results are presented as mean time (±SEM) spent on the rotarod rotating at (A) 6 rpm, (B) 18 rpm and (C) 24 rpm for *n* = 8. Statistical analysis: Student’s *t* test. Significance vs vehicle-treated mice: *p* > 0.05. (D) Changes of mice body weight during the Aβ_1–42_-induced cognitive deficits assay. The values are presented as mean (±SD) for *n* = 6.

### Effects Compound **(*R*)-(−)-3** on B of Mice Body Weight

The body weight of experimental animals is an essential parameter for affirming normal physiological functioning of the internal organs and should be monitored during in vivo experiments. Reduced body weight due to the administered test compounds is one of the primary signs of a toxicological response and considered a vital sign of distress, severe pain or impending death of the animals.^89^ Thus, the body weight of all mice in the Aβ_1–42_-induced cognitive deficits assay was monitored daily. The observed steady body weight gains (Figure 9D) are good indicators of tolerability of compounds **2** and **(** ***R*** **)-(−)-3** and that they are not acutely toxic to mice.

## Conclusions

In our attempt to optimize lead compound **2**, a potent and selective hBChE inhibitor (*K*_i_ = 10.2 nM) with procognitive effects in vivo, we designed, synthesized and evaluated a series of 23 of its derivatives. Compound **(** ***R*** **)-(−)-3** is the most promising compound of the series and superior to compound **2** because it is (i) a pure enantiomer, (ii) a more potent hBChE inhibitor (*K*_i_ = 6.6 nM), (ii) less cytotoxic, (iii) 7 times more brain permeable, (iv) as effective in alleviating scopolamine-induced AD-like cognitive deficits in mice, (v) effective in alleviating Aβ_1–42_-induced AD-like cognitive in mice and (vi) not acutely toxic and does not produce any cholinergic adverse effects or motor deficits in mice. Considering these properties, compound **(** ***R*** **)-(−)-3** is a new promising lead compound for treatment of patients suffering from AD.

## Experimental Section

### General Chemistry Methods

^1^H NMR and ^13^C NMR were recorded at 400.130 and 100.613 MHz, respectively, on an NMR spectrophotometer (Bruker Avance III). Chemical shifts (δ) are reported in parts per million (ppm) and are referenced to the deuterated solvent used. The coupling constants (*J*) are reported in Hz, and indicate the splitting patterns as s, singlet; bs, broad singlet; d, doublet; dd, doublet of doublets; td, triplet of doublets; qd, quartet of doublets; pd, pentet of doublets; m, multiplet; t, triplet; bt, broad triplet; tt, triplet of triplets; dt, doublet of triplets; q, quartet; bq, broad quartet; p, pentet; dp, doublet of pentets. Optical rotations were measured on an Anton Paar MCP 150 polarimeter. Mass spectra were recorded on a LC-MS/MS system (Q Exactive Plus; Thermo Scientific, MA, USA). Melting point of **(** ***R*** **)-(−)-3 HCl** was determined on a Mettler Toledo Melting Point System MP30. Solvents were evaporated under reduced pressure. Solvents and reagents were purchased from abcr, Acros Organics, Alfa Aesar, Euriso-Top, Enamine, Fluka, Merck, Sigma-Aldrich, and TCI Europe, and used without further purification, unless otherwise stated. Anhydrous THF was prepared from commercially available THF which was refluxed under argon over sodium and benzophenone for 2 h, then fractionally distilled under argon through a helices-packed column prior to use. Anhydrous DMF and CH_2_Cl_2_ were purchased from Acros Organics and used without further purification. Flash column chromatography was performed on silica gel 60 for column chromatography (particle size, 230–400 mesh) and analytical thin-layer chromatography was performed on silica gel aluminum sheets (0.20 mm; 60 F254; Merck), with visualization by ultraviolet light and/or visualization reagents. Analytical reversed-phase UPLC methods A and B were recorded on an LC system (Dionex Ultimate 3000 Binary Rapid Separation; Thermo Scientific) equipped with an autosampler, a binary pump system, a photodiode array detector, a thermostated column compartment, and the Chromeleon Chromatography Data System. The detector on the UPLC system was set to 210, 254, and 280 nm. The column used for methods A and B was a C18 analytical column (50 × 2.1 mm, 1.8 μm; Acquity UPLC HSS C18SB) thermostated at 40 °C. Chiral HPLC method C was recorded on an Agilent 1100 LC modular system equipped with an autosampler, a quarternary pump system, a photodiode array detector, a thermostated column compartment, and a ChemStation data system. The detector was set to 210, 254, and 280 nm. The column used for chiral HPLC method C was a CHIRALPAK IB-3, 4.6 × 250 mm, 3 μm column, which was thermostated at 25 °C.

#### UPLC Method A

The sample solution (2 μL; 0.1 mg/mL in CH_3_CN) was injected and eluted at a flow rate of 0.3 mL/min, using a linear gradient of mobile phase A (0.1% [v/v] aqueous TFA) and mobile phase B (CH_3_CN). The gradient for method A (for mobile phase B) was: 0–5 min, 20–90%; 5–7.5 min, 90%; 7.5–8 min, 90–20%.

#### UPLC Method B

The sample solution (2 μL; 0.1 mg/mL in CH_3_CN) was injected and eluted at a flow rate of 0.4 mL/min, using a linear gradient of mobile phase A (0.1% [v/v] aqueous TFA) and mobile phase B (CH_3_CN). The gradient for method A (for mobile phase B) was: 0–8 min, 10–90%; 8–10 min, 90%; 10–11 min, 90–10%.

#### Chiral HPLC Method C

The sample solution [10 μL; 0.5 mg/mL in *i*PrOH or mixture of *i*PrOH + 0.1% (CH_3_CH_2_)_3_N for compound **(** ***R*** **)-(−)-3 HCl**] was injected and eluted for 18 min with a flow rate of 1 mL/min, using a 9/1 [v/v] mixture of *n*-hexane/[(CH_3_)_2_CHOH + 0.1% (CH_3_CH_2_)_3_N] as the mobile phase.

### General Synthetic Procedures

#### General Procedure for Synthesis of Sulfonamide Bond (General Procedure 1)

Amine (1.0 equiv) was dissolved in CH_2_Cl_2_ and cooled to 0 °C. The solution was stirred and (CH_3_CH_2_)_3_N (1.0 equiv) was added dropwise. After 15 min, naphthalene-2-sulfonyl chloride (1.0 equiv) was added, the reaction mixture allowed to warm up to room temperature and stirred for 24 h. The reaction mixture was transferred into a separating funnel, washed with H_2_O, followed by saturated aqueous NaHCO_3_ solution, dried over anhydrous Na_2_SO_4_ and evaporated. The crude product was purified by flash column chromatography.

#### General Procedure for Reductive Amination (General Procedure 2)

Ketone (1.0 equiv) was dissolved in 1,2-dichloroethane at room temperature, the solution stirred and agitated with a stream of argon for 15 min. NaBH­(OAc)_3_ (1.5 equiv) was added followed by 2 M CH_3_NH_2_ in THF, 2 M CH_3_CH_3_NH_2_ in THF, H_2_N­(CH_2_)_2_OCH_3_ or H_2_N­(CH_2_)_3_OCH_3_ (1.0 equiv) and AcOH (1.0 equiv). The resulting suspension was stirred under argon for 24 h, then opened to air and quenched with 1 M aqueous NaOH solution. The mixture was transferred into a separating funnel, and CH_2_Cl_2_ was added. The separating funnel was shaken vigorously, the organic phase was separated, dried over anhydrous Na_2_SO_4_ and evaporated. The crude amine was used in the next step without further purification.

#### General Procedure for Removal of the Benzyloxycarbonyl Group (Cbz) Protecting Group (General Procedure 3)

Cbz protected amine (1.0 equiv) was dissolved in CH_3_OH or a mixture of CH_3_OH and THF at room temperature, the solution stirred and agitated with a stream of argon for 15 min. Pd/C (10 wt %) (20% mass of Cbz protected amine) was added and the resulting suspension agitated with a stream of hydrogen for 15 min. After 24 h the reaction mixture was agitated with a stream of argon for 15 min, filtered with suction through a pad of Celite and evaporated to produce the crude secondary amine which was used in the next step without further purification.

#### General Procedure for Alkylation of Piperidine and Pyrrolidine Nitrogen with Benzyl Bromide (General Procedure 4)

The piperidine (1.0 equiv) was dissolved in acetone at room temperature. The solution was stirred and Cs_2_CO_3_ (1.0 equiv; 2.0 equiv for synthesis of compound **79**) was added, followed by benzyl bromide (1.0 equiv). After 24 h, the reaction mixture was filtered with suction, and the filtrate evaporated. The crude product was purified by flash column chromatography.

#### General Procedure for Synthesis of Amide Bond (General Procedure 5)

Carboxylic acid (1.0 equiv) was dissolved in CH_2_Cl_2_ the solution stirred and cooled to 0 °C. Et_3_N (2.0 equiv) was added dropwise, followed by TBTU (1.0 equiv). The resulting suspension was stirred for 1 h, and during this time the suspension transformed into a solution. Two M solution of CH_3_NH_2_ in THF, 2 M solution of CH_3_CH_2_NH_2_ in THF, H_2_N­(CH_2_)_2_OCH_3_ or H_2_N­(CH_2_)_3_OCH_3_ (2.0 equiv) was added dropwise, the reaction mixture allowed to warm up to room temperature, stirred for 24 h and evaporated. The crude product was purified by flash column chromatography.

#### General Procedure for Reduction of Amide Bonds (General Procedure 6)

To a three-neck round-bottomed flask equipped with a stirring bar and a reflux condenser, LiAlH_4_ (3.5 equiv) was added under an argon atmosphere. Anhydrous THF was added with a double-tipped needle. A solution of diamide (1.0 equiv) in anhydrous THF was added with a double-tipped needle, and the reaction mixture was refluxed for 3 h. The mixture was then cooled to 0 °C and the excess hydride was decomposed by dropwise addition of H_2_O (*n* grams of LiAlH_4_ require *n* mL of H_2_O) followed by 15% aqueous NaOH (*n* grams of LiAlH_4_ require n mL of 15% aqueous NaOH) and then H_2_O (*n* grams of LiAlH_4_ require 3 × *n* mL of H_2_O). After vigorous stirring for 12 h at room temperature, the mixture was filtered under suction and the white precipitate thoroughly washed with THF. The combined filtrates were evaporated to produce the crude amine which was used in the next step without further purification.

#### General Procedure of Methylation of the Sulfonamide Nitrogen (General Procedure 7)

Sulfonamide (1.0 equiv) was dissolved in anhydrous DMF under argon at room temperature and the solution stirred. K_2_CO_3_ (3.0 equiv) was added, followed by CH_3_I (3.0 equiv). After stirring at 60 °C for 24 h the reaction mixture was transferred into a separating funnel. H_2_O was added and the resulting solution was extracted with (CH_3_CH_2_)_2_O. The organic phase was washed with H_2_O, sat. brine solution, dried over anhydrous Na_2_SO_4_ and evaporated to produce the crude methylated sulfonamide which was used in the next step without further purification.

### General Procedure for Removal of the *tert*-Butyloxycarbonyl (BOC) Protecting Group

#### (General Procedure 8)

BOC protected amine (1.0 equiv) was dissolved or suspended in CH_3_OH at room temperature. The solution or suspension was stirred and agitated with a stream of argon for 15 min then cooled to 0 °C. Two M HCl solution in (CH_3_CH_2_)_2_O (3.0 equiv; 20.0 equiv for synthesis of compound **85**) was added, the reaction mixture allowed to warm to room temperature, stirred for 24 h and evaporated. A stirring bar and (CH_3_CH_2_)_2_O were added to the oily residue and the mixture stirred until the oily residue transformed into a white solid. The precipitate was allowed to settle to the bottom of the flask. The supernatant was removed, (CH_3_CH_2_)_2_O was added again, and this procedure was repeated thrice. After the final supernatant was removed, the remaining (CH_3_CH_2_)_2_O was removed by evaporation at reduced pressure to produce the crude hydrochloride, which was used in the next step without further purification.

#### General Procedure for Alkylation of Pyrrolidine Nitrogen with Benzyl Bromide in CH_2_Cl_2_ (General Procedure 9)

Crude amine hydrochloride (1.0 equiv) was dissolved in anhydrous CH_2_Cl_2_ at room temperature, the solution stirred and cooled to 0 °C. (CH_3_CH_2_)_3_N (2.0 equiv) was added dropwise, followed by dropwise addition of benzyl bromide (1.0 equiv). The reaction mixture was allowed to warm to room temperature, stirred for 24 h, transferred into a separating funnel, washed with H_2_O, dried over anhydrous Na_2_SO_4_ and evaporated at room temperature. The crude product was purified by flash column chromatography.

##### Declaration of Purity

All assayed final compounds were ≥95% pure as determined by UPLC.

### Synthesis and Characterization of the Synthesized Compounds

#### Synthesis and Characterization of 1,3-Disubstituted Type A Inhibitors

##### Synthesis of N-(1-Benzylpiperidin-3-yl)­naphthalene-2-sulfonamide (**4**)

Synthesized from compound **14** (0.105 g, 0.552 mmol, 1.0 equiv), naphthalene-2-sulfonyl chloride (0.125 g, 0.552 mmol, 1.0 equiv) and (CH_3_CH_2_)_3_N (0.077 mL, 0.552 mmol, 1.0 equiv) in CH_2_Cl_2_ (10 mL) via general procedure 1. The crude product was purified by flash column chromatography using CH_2_Cl_2_/CH_3_OH (70:1, v/v) as the eluent to produce 0.201 g of compound **4** as a white solid (96% yield). *R*_f_ = 0.38 (CH_2_Cl_2_/CH_3_OH, 50:1, v/v). ^1^H NMR (CDCl_3_): δ 1.40–1.50 (m, 2 H), 1.56 (bs, 1 H), 1.66–1.76 (m, 1 H), 2.13 (bs, 3 H), 2.56 (bs, 1 H), 3.25 (d, *J* = 13.1 Hz, 1 H), 3.40–3.47 (m, 2 H), 5.37 (bs, 1 H), 7.18–7.30 (m, 5 H), 7.57–7.64 (m, 3 H), 7.78 (d, *J* = 8.8 Hz, 1 H), 7.88 (dd, *J*_1_ = 16.8 Hz, *J*_2_ = 7.8 Hz, 2 H), 8.38 (s, 1 H). ^13^C NMR (CDCl_3_): δ 21.64, 30.24, 49.28, 53.24, 57.93, 62.63, 122.00, 126.99, 127.28, 127.73, 127.69, 128.09, 128.47, 128.81, 129.08, 129.30, 131.91, 134.46, 137.66, 137.75. HRMS (ESI+): *m*/*z* calcd for C_22_H_25_N_2_O_2_S 381.1631; found 381.1628. UPLC purity 99%, at 254 nm (method A, *t*_R_ = 2.92 min).

##### Synthesis of Benzyl 3-(Methylamino)­piperidine-1-carboxylate (**18**)

Synthesized from compound **16** (1.250 g, 5.359 mmol, 1.0 equiv), 2 M solution of CH_3_NH_2_ in THF (2.679 mL, 5.359 mmol, 1.0 equiv), NaBH­(OAc)_3_ (1.704 g, 8.039 mmol, 1.5. equiv) and AcOH (0.307 mL, 5.359 mmol, 1.0 equiv) in 1,2-dichloroethane (50 mL) via general procedure 2 to produce 1.225 g of compound **18** as a colorless oil (92% yield). *R*_f_ = 0.33 (CH_2_Cl_2_/CH_3_OH, 2:1, v/v).

##### Synthesis of Benzyl 3-(*N*-Methylnaphthalene-2-sulfonamido)­piperidine-1-carboxylate (**26**)

Synthesized from compound **18** (1.225 g, 4.933 mmol, 1.0 equiv), naphthalene-2-sulfonyl chloride (1.118 g, 4.933 mmol, 1.0 equiv) and (CH_3_CH_2_)_3_N (0.688 mL, 4.933 mmol, 1.0 equiv) in CH_2_Cl_2_ (30 mL) via general procedure 1. The crude product was purified by flash column chromatography using CH_2_Cl_2_/CH_3_OH (75:1, v/v) as the eluent to produce 0.971 g of compound **26** as a colorless oil (45% yield). *R*_f_ = 0.48 (CH_2_Cl_2_/CH_3_OH, 50:1, v/v). ^1^H NMR (CDCl_3_): δ 1.53–1.55 (M, 2 H), 1.69–1.72 (m, 2 H), 2.52 (bs, 1 H), 2.66 (bs, 1 H), 2.81 (s, 3 H), 3.98–4.12 (m, 3 H), 4.95–5.14 (m, 2 H), 7.33–7.40 (m, 5 H), 7.58–7.66 (m, 2 H), 7.71 (bs, 1 H), 7.85–7.97 (m, 3 H), 8.38 (s, 1 H). ^13^C NMR (CDCl_3_): δ 24.63, 28.43, 29.35, 43.76, 46.70, 53.58, 67.22, 122.20, 127.51, 127.88, 127.94, 128.05, 128.31, 128.52, 128.67, 129.20, 129.56, 132.14, 134.71, 136.52, 136.58, 155.08. HRMS (ESI+): *m*/*z* calcd for C_24_H_27_N_2_O_4_S 439.1686; found 439.1722. UPLC purity 99%, at 254 nm (method A, *t*_R_ = 4.580 min).

##### Synthesis of *N*-Methyl-N-(piperidin-3-yl)­naphthalene-2-sulfonamide (**34**)

Synthesized from compound **26** (0.940 g, 2.143 mmol, 1.0 equiv), Pd/C (10 wt %) (0.188 g, 20% mass of compound **26**) and gaseous hydrogen in a mixture of CH_3_OH (35 mL) and THF (10 mL) via general procedure 3 to produce 0.603 g of **34** as a colorless oil (92% yield).

##### Synthesis of N-(1-Benzylpiperidin-3-yl)-*N*-methylnaphthalene-2-sulfonamide (**6**)

Synthesized from compound **34** (0.603 g, 1.981 mmol, 1.0 equiv), Cs_2_CO_3_ (0.645 g, 1.981 mmol, 1.0 equiv) and benzyl bromide (0.235 mL, 1.981 mmol, 1.0 equiv) in acetone (20 mL) via general procedure 4. The crude product was purified by flash column chromatography using CH_2_Cl_2_/CH_3_OH (50:1, v/v) as the eluent to produce 0.700 g of compound **6** as a slightly golden oil (90% yield). *R*_f_ = 0.33 (CH_2_Cl_2_/CH_3_OH, 40:1, v/v). ^1^H NMR (CDCl_3_): δ 1.28–1.36 (m, 1 H), 1.53–1.55 (m, 1 H), 1.59–1.64 (m, 2 H), 1.73–1.79 (m, 1 H), 1.87 (t, *J* = 10.6 Hz, 1 H), 2.69 (dt, *J*_1_ = 10.3, Hz, *J*_2_ = 2.0 Hz, 1 H), 2.75 (bd, *J* = 10.8 Hz, 1 H), 2.81 (s, 3 H), 3.35 (d, *J* = 13.2 Hz, 1 H), 3.36 (d, *J* = 13.2 Hz, 1 H), 4.03–4.11 (m, 1 H), 7.19–7.25 (m, 5 H), 7.58–7.65 (m, 2 H), 7.68 (dd, *J*_1_ = 8.6 Hz, *J*_2_ = 1.9 Hz, 1 H), 7.79 (d, *J* = 8.8 Hz, 1 H), 7.86–7.88 (m, 1 H), 7.92–7.94 (m, 1 H), 8.34 (d, *J* = 1.3 Hz, 1 H). ^13^C NMR (CDCl_3_): δ 24.26, 27.66, 29.26, 52.59, 53.98, 56.06, 62.58, 121.88, 126.69, 127.15, 127.60, 127.71, 127.84, 128.24, 128.51, 128.82, 129.13, 131.78, 134.25, 136.51, 137.66. HRMS (ESI+): *m*/*z* calcd for C_23_H_27_N_2_O_2_S 395.1788; found 395.1785. UPLC purity 99%, at 254 nm (method A, *t*_R_ = 3.23 min).

##### Synthesis of Benzyl 3-(Ethylamino)­piperidine-1-carboxylate (**19**)

Synthesized from compound **16** (1.250 g, 5.359 mmol, 1.0 equiv), 2 M solution of CH_3_CH_2_NH_2_ in THF (2.679 mL, 5.359 mmol, 1.0 equiv), NaBH­(OAc)_3_ (1.704 g, 8.039 mmol, 1.5. equiv) and AcOH (0.307 mL, 5.359 mmol, 1.0 equiv) in 1,2-dichloroethane (50 mL) via general procedure 2 to produce 1.131 g of compound **19** as a colorless oil (80% yield). *R*_f_ = 0.41 (CH_2_Cl_2_/CH_3_OH, 2:1, v/v).

##### Synthesis of Benzyl 3-(N-Ethylnaphthalene-2-sulfonamido)­piperidine-1-carboxylate (**27**)

Synthesized from compound **19** (1.131 g, 4.311 mmol, 1.0 equiv), naphthalene-2-sulfonyl chloride (0.977 g, 4.311 mmol, 1.0 equiv) and (CH_3_CH_2_)_3_N (0.601 mL, 4.311 mmol, 1.0 equiv) in CH_2_Cl_2_ (30 mL) via general procedure 1. The crude product was purified by flash column chromatography using CH_2_Cl_2_/CH_3_OH (100:1, v/v) as the eluent to produce 1.160 g of compound **27** as a colorless oil (59% yield). *R*_f_ = 0.59 (CH_2_Cl_2_/CH_3_OH, 50:1, v/v). ^1^H NMR (CDCl_3_): δ 1.25 (t, *J* = 7.0 Hz, 3 H), 1.47–1.55 (m, 1 H), 1.64 (bs, 1 H), 1.72 (bd, *J* = 13.8 Hz, 1 H), 1.84 (bs, 1 H), 2.55 (bs, 1 H), 2.76 (dd, *J*_1_ = 12.5 Hz, *J*_2_ = 11.5 Hz, 1 H), 3.28 (q, *J* = 7.1 Hz, 2 H), 3.76–3.84 (m, 1 H), 4.12 (bs, 2 H), 4.95–5.13 (m, 2 H), 7.33–7.39 (m, 5 H), 7.58–7.65 (m, 2 H), 7.72 (bs, 1 H), 7.89 (bs, *J* = 27.6 Hz, 3 H), 8.41 (s, 1 H). ^13^C NMR (CDCl_3_): δ 17.41, 24.96, 29.59, 39.39, 43.75, 47.87, 54.98, 67.09, 122.10, 127.41, 127.78, 127.83, 127.95, 128.10, 128.44, 128.55, 129.12, 129.43, 132.06, 134.55, 136.56, 137.95, 154.97. HRMS (ESI+): *m*/*z* calcd for C_25_H_29_N_2_O_4_S 453.1843; found 453.1838. UPLC purity 99%, at 254 nm (method A, *t*_R_ = 4.73 min).

##### Synthesis of N-Ethyl-N-(piperidin-3-yl)­naphthalene-2-sulfonamide (**35**)

Synthesized from compound **27** (1.080 g, 2.386 mmol, 1.0 equiv), Pd/C (10 wt %) (0.216 g, 20% mass of compound **27**) and gaseous hydrogen in CH_3_OH (30 mL) via general procedure 3 to produce 0.658 g of compound **35** as a colorless oil (87% yield).

##### Synthesis of N-(1-Benzylpiperidin-3-yl)-N-ethylnaphthalene-2-sulfonamide (**7**)

Synthesized from compound **35** (0.658 g, 2.066 mmol, 1.0 equiv), Cs_2_CO_3_ (0.673 g, 2.066 mmol, 1.0 equiv) and benzyl bromide (0.246 mL, 2.066 mmol, 1.0 equiv) in acetone (30 mL) via general procedure 4. The crude product was purified by flash column chromatography using CH_2_Cl_2_/CH_3_OH (60:1, v/v) as the eluent to produce 0.711 g of compound **7** as a white solid (84% yield). *R*_f_ = 0.30 (CH_2_Cl_2_/CH_3_OH, 60:1, v/v). ^1^H NMR (CDCl_3_): δ 1.25 (t, *J* = 7.0 Hz, 3 H), 1.38 (qd, *J*_1_ = 12.0 Hz, *J*_2_ = 5.0 Hz, 1 H), 1.59–1.69 (m, 3 H), 1.78 (td, *J*_1_ = 11.4 Hz, *J*_2_ = 3.1 Hz, 1 H), 1.92 (t, *J* = 10.8 Hz, 1 H), 2.74–2.79 (m, 2 H), 3.21–3.34 (m, 2 H), 3.38 (d, *J* = 13.2 Hz, 1 H), 3.46 (d, *J* = 13.2 Hz, 1 H), 3.94 (tt, *J*_1_ = 11.4 Hz, *J*_2_ = 3.7 Hz, 1 H), 7.17–7.23 (m, 5 H), 7.61 (pd, *J*_1_ = 8.7 Hz, *J*_2_ = 1.5 Hz, 2 H), 7.69 (dd, *J*_1_ = 8.7 Hz, *J*_2_ = 1.8 Hz, 1 H), 7.79 (d, *J* = 8.7 Hz, 1 H), 7.86–7.88 (m, 1 H), 7.91–7.93 (m, 1 H), 8.38 (d, *J* = 1.3 Hz, 1 H). ^13^C NMR (CDCl_3_): δ 17.73, 24.76, 29.25, 39.09, 52.89, 55.33, 57.58, 62.80, 122.09, 126.85, 127.25, 127.74, 127.89, 128.02, 128.34, 128.68, 129.06, 129.21, 131.99, 134.40, 137.86, 138.15. HRMS (ESI+): *m*/*z* calcd for C_24_H_29_N_2_O_2_S 409.1944; found 409.1941. UPLC purity 98%, at 254 nm (method A, *t*_R_ = 3.43 min).

##### Synthesis of Benzyl 3-((2-Methoxyethyl)­amino)­piperidine-1-carboxylate (**20**)

Synthesized from compound **16** (1.250 g, 5.359 mmol, 1.0 equiv), NH_2_(CH_2_)_2_OCH_3_ (0.461 mL, 5.359 mmol, 1.0 equiv), NaBH­(OAc)_3_ (1.704 g, 8.039 mmol, 1.5 equiv) and AcOH (0.307 mL, 5.359 mmol, 1.0 equiv) in 1,2-dichloroethane (50 mL) via general procedure 2 to produce 1.397 g of compound **20** as a colorless oil (89% yield). *R*_f_ = 0.41 (CH_2_Cl_2_/CH_3_OH, 9:1, v/v).

##### Synthesis of Benzyl 3-(N-(2-Methoxyethyl)­naphthalene-2-sulfonamido)­piperidine-1-carboxylate (**28**)

Synthesized from compound **20** (1.397 mL, 4.778 mmol, 1.0 equiv), naphthalene-2-sulfonyl chloride (1.083 g, 4.778 mmol, 1.0 equiv) and (CH_3_CH_2_)_3_N (0.666 mL, 4.778, 1.0 equiv) in CH_2_Cl_2_ (25 mL) via general procedure 1. The crude product was purified by flash column chromatography using CH_2_Cl_2_/CH_3_OH (50:1, v/v) as the eluent to produce 0.560 g of compound **28** as a colorless oil (24% yield). *R*_f_ = 0.36 (CH_2_Cl_2_/CH_3_OH, 50:1, v/v). ^1^H NMR (CDCl_3_): δ 1.45–1.55 (m, 1 H), 1.60–1.71 (m, 3 H), 2.54 (bs, 1 H), 2.78 (t, *J* = 11.5 Hz, 1 H), 3.28–3.42 (m, 5 H), 3.52–3.61 (m, 2 H), 3.78 (bs, 1 H), 4.11 (bs, 2 H), 4.93–5.13 (m, 2 H), 7.33–7.39 (m, 5 H), 7.58–7.65 (m, 2 H), 7.73 (bs, 1 H), 7.82–7.98 (m, 3 H), 8.41 (bs, 1 H). ^13^C NMR (CDCl_3_): δ 24.99, 29.31, 43.74, 43.93, 47.79, 55.11, 58.83, 67.12, 72.66, 122.13, 127.51, 127.84, 127.86, 127.98, 128.29, 128.48, 128.69, 129.20, 129.53, 132.10, 134.68, 136.64, 137.48, 155.03. HRMS (ESI+): *m*/*z* calcd for C_26_H_31_N_2_O_5_S 483.1948; found 483.1948. UPLC purity 99%, at 254 nm (method A, *t*_R_ = 4.67 min).

##### Synthesis of N-(2-Methoxyethyl)-N-(piperidin-3-yl)­naphthalene-2-sulfonamide (**36**)

Synthesized from compound **28** (0.523 g, 1.084 mmol, 1.0 equiv), Pd/C (10 wt %) (0.105 g, 20% mass of compound **28**) and hydrogen in CH_3_OH (15 mL) via general procedure 3 to produce 0.309 g of compound **36** as a colorless oil (82% yield).

##### Synthesis of N-(1-Benzylpiperidin-3-yl)-N-(2-methoxyethyl)­naphthalene-2-sulfonamide (**8**)

Synthesized from compound **36** (0.287 g, 0.824 mmol, 1.0 equiv), Cs_2_CO_3_ (0.268 g, 0.447 mmol, 1.0 equiv) and benzyl bromide (0.098 mL, 0.447 mmol, 1.0 equiv) in acetone (15 mL) via general procedure 4. The crude product was purified by flash column chromatography using CH_2_Cl_2_/CH_3_OH (50:1, v/v) as the eluent to produce 0.322 g of compound **8** as a slightly golden oil (85% yield). *R*_f_ = 0.26 (CH_2_Cl_2_/CH_3_OH, 50:1, v/v). ^1^H NMR (CDCl_3_): δ 1.31–1.42 (m, 1 H), 1.51–1.64 (m, 3 H), 1.75–1.81 (m, 1 H), 1.90 (t, *J* = 10.7 Hz, 1 H), 2.72–2.75 (m, 2 H), 3.28–3.40 (m, 6 H), 3.46 (q, *J* = 13.1 Hz, 1 H), 3.57 (t, *J* = 6.8 Hz, 2 H), 3.89–3.96 (m, 1 H), 7.16–7.23 (m, 5 H), 7.58–7.66 (m, 2 H), 7.70 (dd, *J*_1_ = 8.7 Hz, *J*_2_ = 1.9 Hz, 1 H), 7.79 (d, *J* = 8.8 Hz, 1 H), 7.87 (d, *J* = 7.9 Hz, 1 H), 7.92 (d, *J* = 7.8 Hz, 1 H), 8.38 (d, *J* = 1.5 Hz, 1 H). ^13^C NMR (CDCl_3_): δ 24.67, 29.02, 43.35, 52.83, 55.33, 57.27, 58.72, 62.77, 72.73, 122.07, 126.87, 127.31, 127.75, 128.00, 128.04, 128.44, 128.73, 129.08, 129.27, 131.97, 134.48, 137.56, 137.79. HRMS (ESI+): *m*/*z* calcd for C_25_H_31_N_2_O_3_S 439.2050; found 439.2946. UPLC purity 98%, at 254 nm (method A, *t*_R_ = 3.44 min).

##### Synthesis of Benzyl 3-((3-Methoxypropyl)­amino)­piperidine-1-carboxylate (**21**)

Synthesized from compound **16** (1.250 g, 5.359 mmol, 1.0 equiv), 3-methoxypropan-1-amine (0.549 mL, 5.359 mmol, 1.0 equiv), NaBH­(OAc)_3_ (1.704 g, 8.039 mmol, 1.5. equiv) and AcOH (0.307 mL, 5.359 mmol, 1.0 equiv) in 1,2-dichloroethane (50 mL) via general procedure 2 to produce 1.460 g of compound **21** as a colorless oil (89% yield). *R*_f_ = 0.54 (CH_2_Cl_2_/CH_3_OH, 4:1, v/v).

##### Synthesis of Benzyl 3-(N-(3-Methoxypropyl)­naphthalene-2-sulfonamido)­piperidine-1-carboxylate (**29**)

Synthesized from compound **21** (1.460 g, 4.765 mmol, 1.0 equiv), naphthalene-2-sulfonyl chloride (1.080 g, 4.765 mmol, 1.0 equiv) and (CH_3_CH_2_)_3_N (0.664 mL, 4.765 mmol, 1.0 equiv) in CH_2_Cl_2_ (30 mL) via general procedure 1. The crude product was purified by flash column chromatography using CH_2_Cl_2_/CH_3_OH (60:1, v/v) as the eluent to produce 1.110 g of compound **29** as a colorless oil (47% yield). *R*_f_ = 0.32 (CH_2_Cl_2_/CH_3_OH, 60:1, v/v). ^1^H NMR (CDCl_3_): δ 1.46–1.54 (m, 1 H), 1.61–1.71 (m, 2 H), 1.78 (bs, 1 H), 1.90–1.97 (m, 2 H), 2.55 (bs, 1 H), 2.75 (t, *J* = 12.0 Hz, 1 H), 3.25–3.29 (m, 5 H), 3.39 (t, *J* = 5.8 Hz, 2 H), 3.79 (bs, 1 H), 4.10 (bs, 2 H), 4.90–5.12 (m, 2 H), 7.32–7.39 (m, 5 H), 7.58–7.65 (m, 2 H), 7.72 (bs, 1 H), 7.83–7.90 (m, 3 H), 8.40 (s, 1 H). ^13^C NMR (CDCl_3_): δ 25.00, 29.41, 31.95, 42.28, 43.76, 47.71, 55.21, 58.60, 67.15, 69.80, 122.19, 127.48, 127.85, 127.90, 128.01, 128.28, 128.50, 128.64, 129.22, 129.51, 132.13, 134.66, 136.63, 137.68, 155.04. HRMS (ESI+): *m*/*z* calcd for C_27_H_33_N_2_O_5_S 497.2105; found 497.2097. UPLC purity 99%, at 254 nm (method A, *t*_R_ = 4.74 min).

##### Synthesis of N-(3-Methoxypropyl)-N-(piperidin-3-yl)­naphthalene-2-sulfonamide (**37**)

Synthesized from compound **29** (1.080 g, 2.175 mmol, 1.0 equiv), Pd/C (10 wt %) (0.216 g, 20% mass of compound **29**) and gaseous hydrogen in of CH_3_OH (40 mL) via general procedure 3 to produce 0.644 g of compound **37** as a colorless oil (82% yield).

##### Synthesis of N-(1-Benzylpiperidin-3-yl)-N-(3-methoxypropyl)­naphthalene-2-sulfonamide (**9**)

Synthesized from synthesis of compound **37** (0.644 g, 1.774 mmol, 1.0 equiv), Cs_2_CO_3_ (0.579 g, 1.774 mmol, 1.0 equiv) and benzyl bromide (0.211 mL, 1.774 mmol, 1.0 equiv) in acetone (20 mL) via general procedure 4. The crude product was purified by flash column chromatography using CH_2_Cl_2_/CH_3_OH (40:1, v/v) as the eluent to produce 0.727 g of compound **9** as a slightly golden oil (91% yield). *R*_f_ = 0.28 (CH_2_Cl_2_/CH_3_OH, 40:1, v/v). ^1^H NMR (CDCl_3_): δ 1.38 (qd, *J*_1_ = 11.7 Hz, *J*_2_ = 4.6 Hz, 1 H), 1.50–1.58 (m, 1 H), 1.61–1.64 (m, 2 H), 1.79 (dt, *J*_1_ = 11.4 Hz, *J*_2_ = 2.7 Hz, 1 H), 1.89–1.98 (m, 3 H), 2.72–2.75 (m, 2 H), 3.21–3.46 (m, 9 H), 3.89–3.97 (m, 1 H), 7.16–7.22 (m, 5 H), 7.62 (pd, *J*_1_ = 7.3 Hz, *J*_2_ = 1.6 Hz, 2 H), 7.69 (dd, *J*_1_ = 8.7 Hz, *J*_2_ = 1.9 Hz, 1 H), 7.86–7.88 (m, 1 H), 7.92–7.94 (m, 1 H), 8.37 (d, J = 1.5 Hz, 1 H). ^13^C NMR (CDCl_3_): δ 24.47, 28.76, 31.87, 41.67, 52.57, 55.21, 57.08, 58.15, 62.47, 69.60, 121.80, 126.58, 127.04, 127.49, 127.62, 127.74, 128.13, 128.37, 128.74, 128.99, 131.68, 134.13, 137.60. HRMS (ESI+): *m*/*z* calcd for C_26_H_33_N_2_O_3_S 453.2206; found 453.2202. UPLC purity 98%, at 254 nm (method A, *t*_R_ = 3.49 min).

#### Synthesis and Characterization of 1,4-Disubstituted Type A Inhibitors

##### Synthesis of N-(1-Benzylpiperidin-4-yl)­naphthalene-2-sulfonamide (**5**)

Synthesized from compound **15** (0.107 mL, 0.526 mmol, 1.0 equiv), naphthalene-2-sulfonyl chloride (0.119 g, 0.526 mmol, 1.0 equiv) and (CH_3_CH_2_)_3_N (0.073 mL, 0.526 mmol, 1.0 equiv) in CH_2_Cl_2_ (10 mL) via general procedure 1. The crude product was purified by flash column chromatography using CH_2_Cl_2_/CH_3_OH (20:1, v/v) as the eluent to produce 0.192 g of compound **5** as a white solid (96% yield). *R*_f_ = 0.34 (CH_2_Cl_2_/CH_3_OH, 20:1, v/v). ^1^H NMR (CDCl_3_): δ 1.46 (bq, *J* = 9.7 Hz, 2 H), 1.74 (bd, *J* = 10.3 Hz, 2 H), 1.99 (bt, *J* = 9.8 Hz, 2 H), 2.67 (d, *J* = 10.4 Hz, 2 H), 3.23 (bs, 1 H), 3.41 (s, 2 H), 4.65 (bs, 1 H), 7.21–7.26 (m, 5 H), 7.63 (p, *J* = 6.7 Hz, 2 H), 7.83–7.97 (m, 4 H), 8.45 (s, 1 H). ^13^C NMR (CDCl_3_): δ 32.79, 50.77, 51.59, 62.68, 122.20, 126.88, 127.40, 127.78, 128.04, 128.60, 128.88, 129.12, 129.41, 132.00, 134.59, 137.98, 138.02. HRMS (ESI+): *m*/*z* calcd for C_22_H_25_N_2_O_2_S 381.1631; found 381.1628. UPLC purity 99%, at 254 nm (method A, *t*_R_ = 2.97 min).

##### Synthesis of Benzyl 4-(Methylamino)­piperidine-1-carboxylate (**22**)

Synthesized from compound **17** (1.250 g, 5.359 mmol, 1.0 equiv), 2 M solution of CH_3_NH_2_ in THF (2.679 mL, 5.359 mmol, 1.0 equiv), NaBH­(OAc)_3_ (1.704 g, 8.039 mmol, 1.5. equiv) and AcOH (0.307 mL, 5.359 mmol, 1.0 equiv) in 1,2-dichloroethane (50 mL) via general procedure 2 to produce 1.233 g of compound **22** as a colorless oil (93% yield). *R*_f_ = 0.25 (CH_2_Cl_2_/CH_3_OH, 2:1, v/v).

##### Synthesis of Benzyl 4-(*N*-Methylnaphthalene-2-sulfonamido)­piperidine-1-carboxylate (**30**)

Synthesized from compound **22** (1.233 g, 4.965 mmol, 1.0 equiv), naphthalene-2-sulfonyl chloride (1.125 g, 4.965 mmol, 1.0 equiv) and (CH_3_CH_2_)_3_N (0.692 mL, 4.965 mmol, 1.0 equiv) in CH_2_Cl_2_ (30 mL) via general procedure 1. The crude product was purified by flash column chromatography using CH_2_Cl_2_/CH_3_OH (75:1, v/v) as the eluent to produce 1.583 g of compound **30** as a white solid (73% yield). *R*_f_ = 0.28 (CH_2_Cl_2_/CH_3_OH, 75:1, v/v). ^1^H NMR (CDCl_3_): δ 1.48–1.54 (m, 4 H), 2.78 (s, 5 H), 4.04 (septet, *J* = 5.3 Hz, 1 H), 4.20 (bs, 2 H), 5.08 (s, 2 H), 7.28–7.38 (m, 5 H), 7.64 (pd, *J*_1_ = 7.1 Hz, *J*_2_ = 1.5 Hz, 2 H), 7.77 (dd, *J*_1_ = 8.7 Hz, *J*_2_ = 1.8 Hz, 1 H), 7.90–7.93 (m, 1 H), 7.95–7.96 (m, 1 H), 7.97–7.98 (m, 1 H), 8.39 (d, *J* = 1.3 Hz, 1 H). ^13^C NMR (CDCl_3_): δ 28.78, 29.28, 43.36, 54.93, 67.19, 122.15, 127.58, 127.87, 128.02, 128.16, 128.44, 128.72, 129.12, 129.51, 132.16, 134.66, 136.49, 136.80, 154.93. HRMS (ESI+): *m*/*z* calcd for C_24_H_27_N_2_O_4_S 439.1686; found 439.1709. UPLC purity 99%, at 254 nm (method A, *t*_R_ = 4.60 min).

##### Synthesis of *N*-Methyl-N-(piperidin-4-yl)­naphthalene-2-sulfonamide (**38**)

Synthesized from compound **30** (1.541 g, 3.514 mmol, 1.0 equiv), Pd/C (10 wt %) (0.308 g, 20% mass of compound **30**) and gaseous hydrogen in a mixture of CH_3_OH (30 mL) via general procedure 3 to produce 0.946 g of compound **38** as a colorless oil (88% yield).

##### Synthesis of N-(1-Benzylpiperidin-4-yl)-*N*-methylnaphthalene-2-sulfonamide (**10**)

Synthesized from compound **38** (0.946 g, 3.108 mmol, 1.0 equiv), Cs_2_CO_3_ (1.013 g, 3.108 mmol, 1.0 equiv) and benzyl bromide (0.369 mL, 3.108 mmol, 1.0 equiv) in acetone (25 mL) via general procedure 4. The crude product was purified by flash column chromatography using CH_2_Cl_2_/CH_3_OH (50:1, v/v) as the eluent to produce 1.117 g of compound **10** as a white solid (91% yield). *R*_f_ = 0.25 (CH_2_Cl_2_/CH_3_OH, 50:1, v/v). ^1^H NMR (CDCl_3_): δ 1.41–1.44 (m, 2 H), 1.62–1.72 (m, 2 H), 2.00 (t, *J* = 11.3 Hz, 2 H), 2.82–2.86 (m, 5 H), 3.45 (s, 2 H), 3.88 (tt, *J*_1_ = 12.0 Hz, *J*_2_ = 4.1 Hz, 1 H), 7.21–7.25 (m, 3 H), 7.27–7.31 (m, 2 H), 7.58–7.66 (m, 2 H), 7.77 (dd, *J*_1_ = 8.7 Hz, *J*_2_ = 1.8 Hz, 1 H), 7.89–7.97 (m, 3 H), 8.38 (s, 1 H). ^13^C NMR (CDCl_3_): δ 28.76, 29.20, 52.67, 55.22, 62.67, 122.23, 126.92, 127.36, 127.74, 127.95, 128.05, 128.47, 128.90, 129.00, 129.26, 132.06, 134.48, 136.94, 137.97. HRMS (ESI+): *m*/*z* calcd for C_23_H_27_N_2_O_2_S 395.1788; found 395.1785. UPLC purity 98%, at 254 nm (method A, *t*_R_ = 3.25 min).

##### Synthesis of Benzyl 4-(Ethylamino)­piperidine-1-carboxylate (**23**)

Synthesized from compound **17** (1.250 g, 5.359 mmol, 1.0 equiv), 2 M solution of CH_3_CH_2_NH_2_ in THF (2.679 mL, 5.359 mmol, 1.0 equiv), NaBH­(OAc)_3_ (1.704 g, 8.039 mmol, 1.5. equiv) and AcOH (0.307 mL, 5.359 mmol, 1.0 equiv) in 1,2-dichloroethane (50 mL) via general procedure 2 to produce 1.298 g of compound **23** as a colorless oil (92% yield). *R*_f_ = 0.31 (CH_2_Cl_2_/CH_3_OH, 4:1, v/v).

##### Synthesis of Benzyl 4-(N-Ethylnaphthalene-2-sulfonamido)­piperidine-1-carboxylate (**31**)

Synthesized from compound **23** (1.298 g, 4.948 mmol, 1.0 equiv), naphthalene-2-sulfonyl chloride (1.122 g, 4.948 mmol, 1.0 equiv) and (CH_3_CH_2_)_3_N (0.690 mL, 4.948 mmol, 1.0 equiv) in CH_2_Cl_2_ (30 mL) via general procedure 1. The crude product was purified by flash column chromatography using CH_2_Cl_2_/CH_3_OH (60:1, v/v) as the eluent to produce 1.840 g of compound **31** as a white solid (82% yield). *R*_f_ = 0.28 (CH_2_Cl_2_/CH_3_OH, 60:1, v/v). ^1^H NMR (CDCl_3_): δ 1.25 (t, *J* = 7.1 Hz, 3 H), 1.59 (bd, *J* = 2.6 Hz, 4 H), 2.74 (bs, 2 H), 3.27 (q, *J* = 7.1 Hz, 2 H), 3.86–3.94 (m, 1 H), 4.20 (bs, 2 H), 5.09 (s, 2 H), 7.28–7.38 (m, 5 H), 7.59–7.67 (m, 2 H), 7.78 (dd, *J*_1_ = 8.7 Hz, *J*_2_ = 1.8 Hz, 1 H), 7.90–7.97 (m, 3 H), 8.41 (d, *J* = 1.3 Hz, 1 H). ^13^C NMR (CDCl_3_): δ 17.58, 30.75, 38.58, 43.65, 55.96, 67.20, 122.13, 127.53, 127.84, 127.89, 128.02, 128.07, 128.44, 128.65, 129.12, 129.45, 132.14, 136.50, 138.15, 154.93. HRMS (ESI+): *m*/*z* calcd for C_25_H_29_N_2_O_4_S 453.1843; found 453.1873. UPLC purity 99%, at 254 nm (method A, *t*_R_ = 4.75 min).

##### Synthesis of N-Ethyl-N-(piperidin-4-yl)­naphthalene-2-sulfonamide (**39**)

Synthesized from compound **31** (1.796 g, 2.386 mmol, 1.0 equiv), Pd/C (10 wt %) (0.359 g, 20% mass of compound **31**) and gaseous hydrogen in a mixture of CH_3_OH (30 mL) and THF (10 mL) via general procedure 3 to produce 1.197 g of compound **39** as a colorless oil (95% yield).

##### Synthesis of N-(1-Benzylpiperidin-4-yl)-N-ethylnaphthalene-2-sulfonamide (**11**)

Synthesized from compound **39** (1.197 g, 3.759 mmol, 1.0 equiv), Cs_2_CO_3_ (1.224 g, 3.759 mmol, 1.0 equiv) and benzyl bromide (0.446 mL, 3.759 mmol, 1.0 equiv) in acetone (30 mL) via general procedure 4. The crude product was purified by flash column chromatography using CH_2_Cl_2_/CH_3_OH (50:1, v/v) as the eluent to produce 0.711 g of compound **11** as a white solid (84% yield). *R*_f_ = 0.23 (CH_2_Cl_2_/CH_3_OH, 50:1, v/v). ^1^H NMR (CDCl_3_): δ 1.27 (t, *J* = 7.0 Hz, 3 H), 1.51–1.55 (m, 2 H), 1.69 (qd, *J*_1_ = 12.1 Hz, *J*_2_ = 3.6 Hz, 2 H), 1.96 (td, *J*_1_ = 11.8 Hz, *J*_2_ = 2.0 Hz, 2 H), 2.85 (d, *J* = 11.7 Hz, 2 H), 3.31 (q, *J* = 7.1 Hz, 2 H), 3.44 (s, 2 H), 3.76 (tt, *J*_1_ = 12.0 Hz, *J*_2_ = 4.0 Hz, 1 H), 7.20–7.30 (m, 5 H), 7.57–7.64 (m, 2 H), 7.78 (dd, *J*_1_ = 8.7 Hz, *J*_2_ = 1.8 Hz, 1 H), 7.87–7.96 (m, 3 H), 8.40 (d, *J* = 1.2 Hz, 1 H). ^13^C NMR (CDCl_3_): δ 17.66, 30.65, 38.38, 52.96, 56.19, 62.66, 122.21, 126.92, 127.31, 127.71, 127.85, 128.05, 128.39, 128.89, 129.00, 129.20, 132.04, 134.41, 137.99, 138.35. HRMS (ESI+): *m*/*z* calcd for C_24_H_29_N_2_O_2_S 409.1944; found 409.1942. UPLC purity 99%, at 254 nm (method A, *t*_R_ = 3.41 min).

##### Synthesis of Benzyl 4-((2-Methoxyethyl)­amino)­piperidine-1-carboxylate (**24**)

Synthesized from compound **17** (1.250 g, 5.359 mmol, 1.0 equiv), 2-methoxyethan-1-amine (0.461 mL, 5.359 mmol, 1.0 equiv), NaBH­(OAc)_3_ (1.704 g, 8.039 mmol, 1.5 equiv) and AcOH (0.307 mL, 5.359 mmol, 1.0 equiv) in 1,2-dichloroethane (50 mL) via general procedure 2 to produce 1.490 g of compound **24** as a colorless oil (95% yield). *R*_f_ = 0.30 (CH_2_Cl_2_/CH_3_OH (9:1, v/v).

##### Synthesis of Benzyl 4-(N-(2-Methoxyethyl)­naphthalene-2-sulfonamido)­piperidine-1-carboxylate (**32**)

Synthesized from compound **24** (1.490 g, 5.096 mmol, 1.0 equiv), naphthalene-2-sulfonyl chloride (1.155 g, 5.096 mmol, 1.0 equiv) and (CH_3_CH_2_)_3_N (0.710 mL, 5.096 mmol, 1.0 equiv) in CH_2_Cl_2_ (30 mL) via general procedure 1. The crude product was purified by flash column chromatography using CH_2_Cl_2_/CH_3_OH (70:1, v/v) as the eluent to produce 2.187 g of compound **32** as a white solid (89% yield). *R*_f_ = 0.39 (CH_2_Cl_2_/CH_3_OH, 60:1, v/v). ^1^H NMR (CDCl_3_): δ 1.59–1.63 (m, 4 H), 2.73 (bs, 2 H), 3.33–3.35 (m, 5 H), 3.57 (t, *J* = 6.5 Hz, 2 H), 3.79–3.90 (m, 1 H), 4.19 (bs, 2 H), 5.08 (s, 2 H), 7.28–7.39 (m, 5 H), 7.60–7.67 (m, 2 H), 7.79 (dd, *J*_1_ = 8.7 Hz, *J*_2_ = 1.9 Hz, 1 H), 7.91 (dd, *J*_1_ = 7.7 Hz, *J*_2_ = 1.3 Hz, 1 H), 7.95–7.97 (m, 2 H), 8.42 (d, *J* = 1.5 Hz, 1 H). ^13^C NMR (CDCl_3_): δ 30.54, 43.09, 43.59, 56.19, 58.84, 67.21, 72.60, 122.14, 127.61, 127.88, 128.02, 128.21, 128.46, 128.77, 129.16, 129.52, 132.14, 134.69, 136.54, 137.69, 154.92. HRMS (ESI+): *m*/*z* calcd for C_26_H_31_N_2_O_5_S 483.1948; found 483.1947. UPLC purity 99%, at 254 nm (method A, *t*_R_ = 4.66 min).

##### Synthesis of N-(2-Methoxyethyl)-N-(piperidin-4-yl)­naphthalene-2-sulfonamide (**40**)

Synthesized from compound **32** (2.150 g, 4.455 mmol, 1.0 equiv), Pd/C (10 wt %) (0.430 g, 20% mass of compound **32**) and gaseous hydrogen in a mixture of CH_3_OH (30 mL) and THF (25 mL) via general procedure 3 to produce 1.334 g of compound **40** as a colorless oil (86% yield). *R*_f_ = 0.00 (CH_2_Cl_2_/CH_3_OH, 50:1, v/v).

##### Synthesis of N-(1-Benzylpiperidin-4-yl)-N-(2-methoxyethyl)­naphthalene-2-sulfonamide (**12**)

Synthesized from synthesis of compound **40** (1.310 g, 3.759 mmol, 1.0 equiv), Cs_2_CO_3_ (1.225 g, 3.759 mmol, 1.0 equiv) and benzyl bromide (0.447 mL, 3.759 mmol, 1.0 equiv) in acetone (30 mL) via general procedure 4. The crude product was purified by flash column chromatography using CH_2_Cl_2_/CH_3_OH (40:1, v/v) as the eluent to produce 1.504 g of compound **12** as an off-white solid (91% yield). *R*_f_ = 0.27 (CH_2_Cl_2_/CH_3_OH, 40:1, v/v). ^1^H NMR (CDCl_3_): δ 1.48 (dd, *J*_1_ = 12.0 Hz, *J*_2_ = 1.5 Hz, 2 H), 1.68 (qd, *J*_1_ = 12.4 Hz, *J*_2_ = 3.9 Hz, 2 H), 1.96 (td, *J*_1_ = 12.4, *J*_2_ = 2.0 Hz, 2 H), 2.84 (d, *J* = 11.7 Hz, 2 H), 3.34 (s, 3 H), 3.37 (t, *J* = 6.9 Hz, 2 H), 3.43 (s, 2 H), 3.59 (t, *J* = 7.0 Hz, 2 H), 3.69–3.77 (m, 1 H), 7.20–7.25 (m, 3 H), 7.26–7.30 (m, 2 H), 7.58–7.56 (m, 2 H), 7.79 (dd, *J*_1_ = 8.6 Hz, *J*_2_ = 1.9 Hz, 1 H), 7.88–7.96 (m, 3 H), 8.41 (d, *J* = 1.5 Hz, 1 H). ^13^C NMR (CDCl_3_): δ 30.45, 42.61, 52.86, 56.33, 58.74, 62.58, 72.61, 122.21, 126.90, 127.38, 127.73, 128.02, 128.05, 128.51, 128.58, 129.02, 129.26, 132.04, 134.50, 137.80, 138.06. HRMS (ESI+): *m*/*z* calcd for C_25_H_31_N_2_O_3_S 439.2050; found 439.2047. UPLC purity 99%, at 254 nm (method A, *t*_R_ = 3.41 min).

##### Synthesis of Benzyl 4-((3-Methoxypropyl)­amino)­piperidine-1-carboxylate (**25**)

Synthesized from compound **17** (1.250 g, 5.359 mmol, 1.0 equiv), 3-methoxypropan-1-amine (0.549 mL, 5.359 mmol, 1.0 equiv), NaBH­(OAc)_3_ (1.704 g, 8.039 mmol, 1.5 equiv) and AcOH (0.307 mL, 5.359 mmol, 1.0 equiv) in 1,2-dichloroethane (50 mL) via general procedure 2 to produce 1.616 g of compound **25** as a colorless oil (98% yield). *R*_f_ = 0.47 (CH_2_Cl_2_/CH_3_OH (4:1, v/v).

##### Synthesis of Benzyl 4-(N-(3-Methoxypropyl)­naphthalene-2-sulfonamido)­piperidine-1-carboxylate (**33**)

Synthesized from compound **25** (1.611 g, 5.258 mmol, 1.0 equiv), naphthalene-2-sulfonyl chloride (1.192 g, 5.258 mmol, 1.0 equiv) and (CH_3_CH_2_)_3_N (0.733 mL, 5.258 mmol, 1.0 equiv) in CH_2_Cl_2_ (30 mL) via general procedure 1. The crude product was purified by flash column chromatography using CH_2_Cl_2_/CH_3_OH (55:1, v/v) as the eluent to produce 2.508 g of compound **33** as a colorless oil (96% yield). *R*_f_ = 0.47 (CH_2_Cl_2_/CH_3_OH, 50:1, v/v). ^1^H NMR (CDCl_3_): δ 1.56 (bs, 4 H), 1.94 (quint., *J* = 6.8 Hz, 2 H), 2.73 (bs, 2 H), 3.27 (t, *J* = 7.7 Hz, 2 H), 3.30 (s, 3 H), 3.40 (t, *J* = 5.9 Hz, 2 H), 3.84–3.92 (m, 1 H), 4.19 (bs, 2 H), 5.08 (s, 2 H), 7.28–7.38 (m, 5 H), 7.59–7.67 (m, 2 H), 7.78 (dd, *J*_1_ = 8.7 Hz, *J*_2_ = 1.8 Hz, 1 H), 7.90–7.97 (m, 3 H), 8.41 (d, *J* = 1.5 Hz, 1 H). ^13^C NMR (CDCl_3_): δ 30.48, 31.99, 41.49, 43.57, 56.20, 58.58, 67.19, 69.88, 122.13, 127.56, 127.85, 127.88, 128.01, 128.13, 128.44, 128.70, 129.14, 129.48, 132.13, 134.61, 136.49, 137.83, 154.90. HRMS (ESI+): *m*/*z* calcd for C_27_H_33_N_2_O_5_S 497.2105; found 497.2104. UPLC purity 99%, at 254 nm (method A, *t*_R_ = 4.73 min).

##### Synthesis of N-(3-Methoxypropyl)-N-(piperidin-4-yl)­naphthalene-2-sulfonamide (**41**)

Synthesized from compound **33** (2.398 g, 4.829 mmol, 1.0 equiv), Pd/C (10 wt %) (0.480 g, 20% mass of compound **33**) and gaseous hydrogen in CH_3_OH (40 mL) via general procedure 3 to produce 1.506 g of compound **41** as a colorless oil (86% yield).

##### Synthesis of N-(1-Benzylpiperidin-4-yl)-N-(3-methoxypropyl)­naphthalene-2-sulfonamide (**13**)

Synthesized from compound **41** (1.506 g, 4.155 mmol, 1.0 equiv), Cs_2_CO_3_ (1.354 g, 4.155 mmol, 1.0 equiv) and benzyl bromide (0.494 mL, 4.155 mmol, 1.0 equiv) in acetone (35 mL) via general procedure 4. The crude product was purified by flash column chromatography using CH_2_Cl_2_/CH_3_OH (40:1, v/v) as the eluent to produce 1.202 g of compound **13** as a white solid (37% yield). *R*_f_ = 0.28 (CH_2_Cl_2_/CH_3_OH, 40:1, v/v). ^1^H NMR (CDCl_3_): δ 1.49 (bd, *J* = 11.2 Hz, 2 H), 1.65–1.74 (m, 2 H), 1.92–2.01 (m, 4 H), 2.84 (d, *J* = 11.4 Hz, 2 H), 3.29–3.33 (m, 5 H), 3.40–3.43 (m, 4 H), 3.47 (tt, *J*_1_ = 12.0 Hz, *J*_2_ = 3.8 Hz, 1 H), 7.21–7.30 (m, 5 H), 7.58–7.64 (m, 2 H), 7.78 (dd, *J*_1_ = 8.7 Hz, *J*_2_ = 1.5 Hz, 1 H), 7.87–7.96 (m, 3 H), 8.39 (s, 1 H). ^13^C NMR (CDCl_3_): δ 30.42, 32.06, 41.35, 52.95, 56.43, 58.45, 62.67, 69.99, 122.23, 126.92, 127.33, 127.72, 127.92, 128.05, 128.44, 128.92, 129.02, 129.22, 132.05, 134.45, 137.93, 138.04. HRMS (ESI+): *m*/*z* calcd for C_26_H_33_N_2_O_3_S 453.2206; found 453.2202. UPLC purity 99%, at 254 nm (method A, *t*_R_ = 3.48 min).

#### Synthesis and Characterization of Type B Inhibitors

##### Synthesis of N-((1-Benzylpyrrolidin-3-yl)­methyl)­naphthalene-2-sulfonamide (**42**)

Synthesized from compound **43** (0.100 g, 0.526 mmol, 1.0 equiv), naphthalene-2-sulfonyl chloride (0.119 g, 0.526 mmol, 1.0 equiv) and (CH_3_CH_2_)_3_N (0.073 mL, 0.526 mmol, 1.0 equiv) in CH_2_Cl_2_ (15 mL) via general procedure 1. The crude product was purified by flash column chromatography using CH_2_Cl_2_/CH_3_OH (20:1, v/v) as the eluent to produce 0.160 g of compound **42** as a white solid (80% yield). *R*_f_ = 0.45 (CH_2_Cl_2_/CH_3_OH, 10:1, v/v). ^1^H NMR (CDCl_3_): δ 1.50–1.58 (m, 1 H), 1.90–1.99 (m, 1 H), 2.21–2.37 (m, 4 H), 2.85–2.92 (m, 2 H), 3.00 (dd, *J*_1_ = 10.6 Hz, *J*_2_ = 2.2 Hz, 1 H), 3.41 (dd, *J*_1_ = 84.5 Hz, *J*_2_ = 12.6. Hz, 2 H), 6.33 (bs, 1 H), 7.31–7.38 (m, 5 H), 7.57–7.64 (m, 3 H), 7.83–7.92 (m, 3 H), 8.36 (s, 1 H). ^13^C NMR (CDCl_3_): δ 27.49, 35.44, 48.32, 53.78, 57.86, 59.90, 122.24, 127.21, 127.37, 127.80, 128.37, 128.48, 128.54, 128.79, 129.14, 129.33, 132.02, 134.59, 136.42, 138.54. HRMS (ESI+): *m*/*z* calcd for C_22_H_25_N_2_O_2_S 381.1631; found 381.1621. UPLC purity 99%, at 254 nm (method A, *t*_R_ = 3.02 min).

##### Synthesis of 1-Benzoylpyrrolidine-3-carboxylic Acid (**48**)

Compound **47** (10.000 g, 65.967 mmol, 1.0 equiv) was added into a 500 mL round-bottom flask equipped with a magnetic stirring bar. One M aqueous NaOH solution (180 mL) cooled to 0 °C was added, the resulting solution stirred and cooled to 0 °C. A solution of benzoyl chloride (7.657 mL, 65.967 mmol, 1.0 equiv) in 1,4-dioxane (60 mL) was added dropwise via a dropping funnel. The pH of the reaction mixture was adjusted to 9 using 1 M aqueous NaOH solution, allowed to warm to room temperature and stirred for 24 h. The pH of the reaction mixture was adjusted to 9 using 1 M aqueous NaOH solution, transferred into a 500 mL separating funnel and washed with (CH_3_CH_2_)_2_O (3 × 150 mL). The aqueous phase was transferred into a 500 mL Erlenmeyer flask, a stirring bar was added, the solution stirred and cooled to 0 °C. The pH was adjusted to 1–2 with concentrated H_2_SO_4_, transferred into a 1-L separating funnel and extracted with CH_2_Cl_2_ (2 × 300 mL). The combined organic phases were transferred into a 2-L separating funnel, washed with saturated NaCl solution (600 mL), dried over Na_2_SO_4_, and evaporated. The crude product was recrystallized from EtOAc to produce 12.001 g of compound **48** as a white solid (83%). *R*_f_ = 0.46 (CH_2_Cl_2_/CH_3_OH/AcOH, 10:1:0.1, v/v). ^1^H NMR (CDCl_3_): δ 2.09–2.32 (m, 2 H), 3.13 (dp, *J*_1_ = 45.1 Hz, *J*_2_ = 7.0 Hz, 1 H), 3.46–3.82 (m, 3 H), 3.93 (d, *J* = 7.0 Hz, 1 H), 7.37–7.52 (m, 5 H), 9.17 (bs, 1 H). ^13^C NMR (CDCl_3_): δ 27.60, 29.25, 41.60, 43.39, 45.64, 48.49, 48.79, 51.22, 127.01, 127.04, 128.24, 128.28, 130.21, 135.76, 170.13, 170.27, 175.43, 176.30. HRMS (ESI+): *m*/*z* calcd for C_12_H_14_NO_3_ 220.0964; found 220.0968. UPLC purity 99%, at 254 nm (method A, *t*_R_ = 1.45 min).

##### Synthesis of 1-Benzoyl-*N*-methylpyrrolidine-3-carboxamide (**49**)

Synthesized from compound **48** (2.900 g, 13.228 mmol, 1.0 equiv), (CH_3_CH_2_)_3_N (3.687 mL, 26.455 mmol, 2.0 equiv), TBTU (4.247 g, 13.228 mmol, 1.0 equiv), and 2 M solution of CH_3_NH_2_ in THF (13.228 mL, 26.455 mmol, 2.0 equiv) in CH_2_Cl_2_ (75 mL) via general procedure 5. The crude product was purified by flash column chromatography using CHCl_3_/CH_3_OH (20:1, v/v) as the eluent to produce 3.020 g of **49** as a colorless oil (98% yield). *R*_f_ = 0.41 (CH_2_Cl_2_/CH_3_OH, 10:1, v/v). ^1^H NMR (CDCl_3_): δ 2.02–2.27 (m, 2 H), 2.77–2.96 (m, 4 H), 5.90 (bd, *J* = 30.4 Hz, 1 H), 7.36–7.44 (m, 3 H), 7.49–7.53 (m, 2 H). ^13^C NMR (CDCl_3_): δ 26.12, 26.21, 28.38, 29.94, 42.83, 44.79, 45.85, 48.97, 49.01, 51.96, 126.81, 126.89, 128.25, 129.98, 130.01, 136.43, 136.45, 169.61, 169.64, 171.02, 172.94. HRMS (ESI+): *m*/*z* calcd for C_13_H_17_N_2_O_2_ 233.1285; found 233.1279. UPLC purity 99%, at 254 nm (method A, *t*_R_ = 1.51 min).

##### Synthesis of 1-(1-Benzylpyrrolidin-3-yl)-*N*-methylmethanamine (**53**)

Synthesized from compound **49** (2.995 g, 12.894 mmol, 1.0 equiv) and LiAlH_4_ (1.713 g, 45.138 mmol, 3.5 equiv) in anhydrous THF (approximately 90 mL) via general procedure 6 to produce 2.570 g of compound **53** as a colorless liquid (98% crude). HRMS (ESI+): *m*/*z* calcd for C_13_H_21_N_2_ 205.1699; found 205.1700.

##### Synthesis of N-((1-Benzylpyrrolidin-3-yl)­methyl)-*N*-methylnaphthalene-2-sulfonamide (**3**)

Synthesized from compound **53** (0.100 g, 0.489 mmol, 1.0 equiv), naphthalene-2-sulfonyl chloride (0.111 g, 0.489 mmol, 1.0 equiv) and (CH_3_CH_2_)_3_N (0.068 mL, 0.489 mmol, 1.0 equiv) in CH_2_Cl_2_ (10 mL) via general procedure 1. The crude product was purified by flash column chromatography using CH_2_Cl_2_/CH_3_OH (30:1, v/v) as the eluent to produce 0.152 g of compound **3** as a colorless oil (78% yield). *R*_f_ = 0.50 (CH_2_Cl_2_/CH_3_OH, 10:1, v/v). ^1^H NMR (CDCl_3_): δ 1.51–1.59 (m, 1 H), 1.92–2.01 (m, 1 H), 2.30 (dd, *J*_1_ = 9.4 Hz, *J*_2_ = 6.2 Hz, 1 H), 2.41–2.50 (m, 1 H), 2.52–2.58 (m, 1 H), 2.60–2.65 (m, 1 H), 2.70–2.75 (m, 4 H), 2.98 (dd, *J*_1_ = 12.8 Hz, *J*_2_ = 7.3 Hz, 1 H), 3.07 (dd, *J*_1_ = 12.7 Hz, *J*_2_ = 8.2 Hz, 1 H), 3.60 (s, 2 H), 7.21–7.28 (m, 1 H), 7.29–7.32 (m, 4 H), 7.64 (dp, *J*_1_ = 7.1 Hz, *J*_2_ = 1.5 Hz, 2 H), 7.76 (dd, *J*_1_ = 8.6 Hz, *J*_2_ = 1.8 Hz, 1 H), 7.92 (d, *J* = 7.8 Hz, 1 H), 7.97 (t, *J* = 7.9 Hz, 2H), 8.35 (d, *J* = 1.1 Hz, 1 H). ^13^C NMR (CDCl_3_): δ 27.99, 34.89, 35.51, 53.33, 54.41, 57.75, 60.14, 122.54, 126.74, 127.35, 127.71, 128.05, 128.45, 128.53, 128.59, 128.98, 129.09, 132.01, 134.18, 134.54, 138.84. HRMS (ESI+): *m*/*z* calcd for C_23_H_27_N_2_O_2_S 395.1788; found 395.1783. UPLC purity 99%, at 254 nm (method A, *t*_R_ = 3.36 min).

##### Synthesis of 1-Benzoyl-N-ethylpyrrolidine-3-carboxamide (**50**)

Synthesized from compound **48** (2.900 g, 13.228 mmol, 1.0 equiv), (CH_3_CH_2_)_3_N (3.687 mL, 26.455 mmol, 2.0 equiv), TBTU (4.247 g, 13.228 mmol, 1.0 equiv), and 2 M solution of CH_3_CH_2_H_2_N in THF (13.228 mL, 26.455 mmol, 2.0 equiv) in CH_2_Cl_2_ (75 mL) via general procedure 5. The crude product was purified by flash column chromatography using CHCl_3_/CH_3_OH (20:1, v/v) as the eluent to produce 3.054 g of compound **50** as a colorless oil (94% yield). *R*_f_ = 0.45 (CH_2_Cl_2_/CH_3_OH, 10:1, v/v). ^1^H NMR (CDCl_3_): δ 1.09–1.17 (m, 3 H), 2.03–2.27 (m, 2 H), 2.74–2.94 (m, 1 H), 3.23–3.35 (m, 2 H), 3.42–3.97 (m, 4 H), 5.73 (bd, *J* = 23.8 Hz, 1 H), 7.36–7.44 (m, 3 H), 7.50–7.52 (m, 2 H). ^13^C NMR (CDCl_3_): δ 14.49, 14.55, 28.41, 29.91, 34.26, 34.32, 42.90, 44.80, 45.84, 48.96, 49.00, 51.93, 126.82, 126.89, 128.21, 129.93, 129.69, 136.44, 136.47, 169.54, 169.59, 171.19, 172.12.^94^ HRMS (ESI+): *m*/*z* calcd for C_14_H_19_N_2_O_2_ 247.1436; found 247.1441. UPLC purity 99%, at 254 nm (method A, *t*_R_ = 1.91 min).

##### Synthesis of N-((1-Benzylpyrrolidin-3-yl)­methyl)­ethanamine (**54**)

Synthesized from compound **50** (2.934 g, 11.920 mmol, 1.0 equiv) and LiAlH_4_ (1.584 g, 41.818 mmol, 3.5 equiv) in anhydrous THF (cca. 90 mL) via general procedure 6 to produce 2.506 g of compound **54** as a colorless liquid (96% crude). HRMS (ESI+): *m*/*z* calcd for C_14_H_23_N_2_ 219.1856; found 219.1854.

##### Synthesis of N-((1-Benzylpyrrolidin-3-yl)­methyl)-N-ethylnaphthalene-2-sulfonamide (**44**)

Synthesized from compound **54** (0.100 g, 0.458 mmol, 1.0 equiv), naphthalene-2-sulfonyl chloride (0.104 g, 0.458 mmol, 1.0 equiv) and (CH_3_CH_2_)_3_N (0.064 mL, 0.458 mmol, 1.0 equiv) in CH_2_Cl_2_ (10 mL) via general procedure 1. The crude product was purified by flash column chromatography using CH_2_Cl_2_/CH_3_OH (30:1, v/v) as the eluent to produce 0.156 g of compound **44** as a colorless oil (83% yield). *R*_f_ = 0.48 (CH_2_Cl_2_/CH_3_OH, 10:1, v/v). ^1^H NMR (CDCl_3_): δ 1.07 (t, *J* = 7.1 Hz, 3 H), 1.50–1.58 (m, 1 H), 1.91–2.00 (m, 1 H), 2.29 (dd, *J*_1_ = 9.2 Hz, *J*_2_ = 6.1 Hz, 1 H), 2.45–2.63 (m, 3 H), 2.69 (dd, *J*_1_ = 9.2 Hz, *J*_2_ = 7.6 Hz, 1 H), 3.17 (s, 1 H), 3.19 (d, *J* = 1.3 Hz, 1 H), 3.20–3.31 (m, 2 H), 3.57 (s, 2 H), 7.22–7.26 (m, 1 H), 7.27–7.33 (m, 4 H), 7.59–7.66 (m, 2 H), 7.77 (dd, *J*_1_ = 8.6 Hz, *J*_2_ = 1.9 Hz, 1 H), 7.89–7.97 (m, 3 H), 8.38 (d, *J* = 1.5 Hz, 1 H). ^13^C NMR (CDCl_3_): δ 13.85, 28.04, 36.16, 42.89, 51.85, 53.28, 57.74, 60.11, 122.34, 126.75, 127.29, 127.68, 128.04, 128.09, 128.42, 128.59, 128.96, 129.12, 131.99, 134.43, 136.66, 138.72. HRMS (ESI+): *m*/*z* calcd for C_24_H_29_N_2_O_2_S 409.1944; found 409.1937. UPLC purity 99%, at 254 nm (method A, *t*_R_ = 3.53 min).

##### Synthesis of 1-Benzoyl-N-(2-methoxyethyl)­pyrrolidine-3-carboxamide (**51**)

Synthesized from compound **48** (2.900 g, 13.228 mmol, 1.0 equiv), (CH_3_CH_2_)_3_N (3.687 mL, 26.455 mmol, 2.0 equiv), TBTU (4.247 g, 13.228 mmol, 1.0 equiv), and H_2_N­(CH_2_)_2_OMe (2.284 mL, 26.455 mmol, 2.0 equiv) in CH_2_Cl_2_ (75 mL) via general procedure 5. The crude product was purified by flash column chromatography using CHCl_3_/CH_3_OH (20:1, v/v) as the eluent to produce 3.236 g of compound **51** as a colorless oil (89% yield). *R*_f_ = 0.45 (CH_2_Cl_2_/CH_3_OH, 10:1, v/v). ^1^H NMR (CDCl_3_): δ 2.04–2.25 (m, 2 H), 2.79–2.98 (m, 1 H), 3.33–3.37 (m, 3 H), 3.40–3.99 (m, 8 H), 6.01 (bs, *J* = 21.0 Hz, 1 H), 7.37–7.44 (m, 3 H), 7.52 (d, *J* = 7.5 Hz, 2 H). ^13^C NMR (CDCl_3_): δ 28.47, 29.91, 39.11, 39.17, 43.02, 45.02, 45.79, 48.90, 48.97, 51.88, 58.58, 58.62, 70.76, 70.85, 126.93, 126.99, 128.19, 129.89, 129.93, 136.43, 169.55, 169.57, 171.40, 172.31.^94^ HRMS (ESI+): *m*/*z* calcd for C_15_H_21_N_2_O_3_ 277.1547; found 277.1540. UPLC purity 99%, at 254 nm (method A, *t*_R_ = 1.84 min).

##### Synthesis of N-((1-Benzylpyrrolidin-3-yl)­methyl)-2-methoxyethan-1-amine (**55**)

Synthesized from compound **51** (3.107 g, 11.244 mmol, 1.0 equiv) and LiAlH_4_ (1.494 g, 39.368 mmol, 3.5 equiv) in anhydrous THF (cca. 90 mL) via general procedure 6 to produce 2.735 g of compound **55** as a colorless liquid (98% crude). HRMS (ESI+): *m*/*z* calcd for C_15_H_25_N_2_O 249.1961; found 249.1957.

##### Synthesis of N-((1-Benzylpyrrolidin-3-yl)­methyl)-N-(2-methoxyethyl)­naphthalene-2-sulfonamide (**45**)

Synthesized from compound **55** (0.100 g, 0.403 mmol, 1.0 equiv), naphthalene-2-sulfonyl chloride (0.091 g, 0.403 mmol, 1.0 equiv) and (CH_3_CH_2_)_3_N (0.056 mL, 0.403 mmol, 1.0 equiv) in CH_2_Cl_2_ (10 mL) via general procedure 1. The crude product was purified by flash column chromatography using CH_2_Cl_2_/CH_3_OH (30:1, v/v) as the eluent to produce 0.153 g of compound **45** as a colorless oil (86% yield). *R*_f_ = 0.45 (CH_2_Cl_2_/CH_3_OH, 10:1, v/v). ^1^H NMR (CDCl_3_): δ 1.47–1.56 (m, 1 H), 1.89–1.98 (m, 1 H), 2.27 (dd, *J*_1_ = 9.1 Hz, *J*_2_ = 5.9 Hz, 1 H), 2.48–2.62 (m, 3 H), 2.67 (t, *J* = 8.3 Hz, 1 H), 3.16–3.26 (m, 5 H), 3.31 (t, *J* = 6.2 Hz, 2 H), 3.50 (t, *J* = 6.2 Hz, 2 H), 3.57 (s, 2 H), 7.21–7.25 (m, 1 H), 7.27–7.33 (m, 4 H), 7.59–7.66 (m, 2 H), 7.78 (dd, *J*_1_ = 8.7 Hz, *J*_2_ = 1.8 Hz, 1 H), 7.90–7.98 (m, 3 H), 8.39 (d, *J* = 1.5 Hz, 1 H). ^13^C NMR (CDCl_3_): δ 28.00, 36.13, 47.82, 53.30, 53.73, 57.70, 58.56, 60.13, 71.17, 122.42, 126.72, 127.32, 127.68, 128.03, 128.26, 128.48, 128.58, 128.98, 129.08, 131.96, 134.49, 136.23, 138.81. HRMS (ESI+): *m*/*z* calcd for C_25_H_31_N_2_O_3_S 439.2050; found 439.2043. UPLC purity 99%, at 254 nm (method A, *t*_R_ = 3.50 min).

##### Synthesis of 1-Benzoyl-N-(3-methoxypropyl)­pyrrolidine-3-carboxamide (**52**)

Synthesized from compound **48** (2.900 g, 13.228 mmol, 1.0 equiv), (CH_3_CH_2_)_3_N (3.687 mL, 26.455 mmol, 2.0 equiv), TBTU (4.247 g, 13.228 mmol, 1.0 equiv), and H_2_N­(CH_2_)_3_OMe (2.711 mL, 26.455 mmol, 2.0 equiv) in CH_2_Cl_2_ (75 mL) via general procedure 5. The crude product was purified by flash column chromatography using CHCl_3_/CH_3_OH (20:1, v/v) as the eluent to produce 3.352 g of compound **52** as a colorless oil (87% yield). *R*_f_ = 0.43 (CH_2_Cl_2_/CH_3_OH, 10:1, v/v). IR (ATR): ^1^H NMR (CDCl_3_): δ 1.72–1.82 (m, 2 H), 2.03–2.25 (m, 2 H), 2.76–2.95 (m, 1 H), 3.29–3.98 (m, 11 H), 6.32 (bs, 1 H), 7.37–7.44 (m, 3 H), 7.51–7.53 (m, 2 H). ^13^C NMR (CDCl_3_): δ 28.35, 28.77, 28.87, 29.84, 38.02, 43.08, 45.08, 45.80, 48.89, 48.95, 51.86, 58.62, 58.66, 71.55, 126.88, 126.95, 128.18, 129.87, 129.91, 136.48, 136.51, 169.52, 169.55, 171.13, 172.06.^94^ HRMS (ESI+): *m*/*z* calcd for C_16_H_23_N_2_O_3_ 291.1703; found 291.1696. UPLC purity 99%, at 254 nm (method A, *t*_R_ = 2.13 min).

##### Synthesis of N-((1-Benzylpyrrolidin-3-yl)­methyl)-3-methoxypropan-1-amine (**56**)

Synthesized from compound **52** (2.884 g, 9.932 mmol, 1.0 equiv) and LiAlH_4_ (1.319 g, 34.756 mmol, 3.5 equiv) in anhydrous THF (cca. 90 mL) via general procedure 6 to produce 2.560 g of compound **56** as a colorless liquid (98% crude). HRMS (ESI+): *m*/*z* calcd for C_16_H_27_N_2_O 263.2118; found 263.2114.

##### Synthesis of N-((1-Benzylpyrrolidin-3-yl)­methyl)-N-(3-methoxypropyl)­naphthalene-2-sulfonamide (**46**)

Synthesized from compound **56** (0.100 g, 0.381 mmol, 1.0 equiv), naphthalene-2-sulfonyl chloride (0.086 g, 0.381 mmol, 1.0 equiv) and (CH_3_CH_2_)_3_N (0.053 mL, 0.381 mmol, 1.0 equiv) in CH_2_Cl_2_ (10 mL) via general procedure 1. The crude product was purified by flash column chromatography using CH_2_Cl_2_/CH_3_OH (30:1, v/v) as the eluent to produce 0.160 g of compound **46** as a colorless oil (93% yield). *R*_f_ = 0.48 (CH_2_Cl_2_/CH_3_OH, 10:1, v/v). ^1^H NMR (CDCl_3_): δ 1.47–1.55 (m, 1 H), 1.77–1.84 (m, 2 H), 1.89–1.98 (m, 1 H), 2.27 (dd, *J*_1_ = 9.2 Hz, *J*_2_ = 5.7 Hz, 1 H), 2.44–2.59 (m, 3 H), 2.64 (dd, *J*_1_ = 9.2 Hz, *J*_2_ = 7.5 Hz, 1 H), 3.15 (s, 1 H), 3.17 (d, *J* = 1.3 Hz, 1 H), 3.22 (q, *J* = 7.4 Hz, 2 H), 3.26 (s, 3 H), 3.34 (t, *J* = 5.9 Hz, 2 H), 3.56 (s, 2 H), 7.22–7.25 (m, 1 H), 7.27–7.32 (m, 4 H), 7.59–7.66 (m, 2 H), 7.77 (dd, *J*_1_ = 8.6 Hz, *J*_2_ = 1.8 Hz, 1 H), 7.89–7.98 (m, 3 H), 8.38 (d, *J* = 1.5 Hz, 1 H). ^13^C NMR (CDCl_3_): δ 28.09, 29.09, 36.11, 46.05, 53.14, 53.29, 57.72, 58.38, 60.11, 69.60, 122.39, 126.72, 127.30, 127.68, 128.02, 128.22, 128.46, 128.57, 128.99, 129.12, 131.99, 134.47, 136.20, 138.83. HRMS (ESI+): *m*/*z* calcd for C_26_H_33_N_2_O_3_S 453.2206; found 453.2200. UPLC purity 99%, at 254 nm (method A, *t*_R_ = 3.56 min).

##### Synthesis of *tert*-Butyl (S)-3-((Naphthalene-2-sulfonamido)­methyl)­pyrrolidine-1-carboxylate (**87**)

Synthesized from compound **86** (4.950 g, 24.715 mmol, 1.0 equiv), naphthalene-2-sulfonyl chloride (5.602 g, 24.715 mmol, 1.0 equiv) and (CH_3_CH_2_)_3_N (3.445 mL, 24.715 mmol, 1.0 equiv) in CH_2_Cl_2_ (150 mL) via general procedure 1 to produce 9.555 g of crude compound **87** as a white solid (99% yield). This product was used in the next step without further purification. *R*_f_ = 0.36 (CH_2_Cl_2_/CH_3_OH, 30:1, v/v). HRMS (ESI+): *m*/*z* calcd for C_20_H_27_N_2_O_4_S 391.1686; found 391.1681.

##### Synthesis of *tert*-Butyl (S)-3-((*N*-methylnaphthalene-2-sulfonamido)­methyl)­pyrrolidine-1-carboxylate (**88**)

Synthesized from compound **87** (4.752 g, 12.169 mmol, 1.0 equiv), K_2_CO_3_ (5.046 g, 36.510 mmol, 3.0 equiv) and CH_3_I (2.273 mL, 36.510 mmol, 3.0 equiv) in anhydrous DMF (48 mL) via general procedure 7 to produce 4.809 g of crude compound **88** as a white solid (98% yield). *R*_f_ = 0.70 (CH_2_Cl_2_/CH_3_OH, 20:1, v/v). HRMS (ESI+): *m*/*z* calcd for C_21_H_29_N_2_O_4_S 405.1843; found 405.1839.

##### Synthesis of (S)-*N*-Methyl-N-(pyrrolidin-3-ylmethyl)­naphthalene-2-sulfonamide hydrochloride (**89**)

Synthesized for compound **88** (4.389 g, 10.850 mmol, 1.0 equiv) and 2 M HCl solution in (CH_3_CH_2_)_2_O (17 mL, 32.550 mmol, 3.0 equiv) via general procedure 8 to produce 3.616 g of crude compound **89** as a white solid (98% yield). *R*_f_ = 0.06 (CH_2_Cl_2_/CH_3_OH, 20:1, v/v). HRMS (ESI+): *m*/*z* calcd for C_16_H_21_N_2_O_2_S 305.1318; found 305.1314.

##### Synthesis of (S)-N-((1-Benzylpyrrolidin-3-yl)­methyl)-*N*-methylnaphthalene-2-sulfonamide [***S*-(+)-3**]

Synthesized from compound **89** (3.562 g, 10.473 mmol, 1.0 equiv), (CH_3_CH_2_)_3_N (2.920 mL, 20.947 mmol, 2.0 equiv) and benzyl bromide (1.244 mL, 10.473 mmol, 1.0 equiv) in anhydrous CH_2_Cl_2_ (150 mL) via general procedure 9. The crude product was purified by flash column chromatography using CH_2_Cl_2_/CH_3_OH (25:1, v/v) as the eluent to produce 2.747 g of compound ***S*** **-(+)-3** as a white solid (66% yield). *R*_f_ = 0.49 (CH_2_Cl_2_/CH_3_OH, 10:1, v/v). [α]_D_^25^ 17.80 (c 0.5, CHCl_3_). ^1^H NMR (CDCl_3_): δ 1.51–1.59 (m, 1 H), 1.92–2.01 (m, 1 H), 2.29 (dd, *J*_1_ = 9.4; *J*_2_ = 6.1 Hz, 1 H), 2.40–2.50 (m, 1 H), 2.51–2.57 (m, 1 H), 2.60–2.65 (m, 1 H), 2.69–2.75 (m, 4 H), 2.98 (dd, *J*_1_ = 12.8; *J*_2_ = 7.3 Hz, 1 H), 3.06 (dd, *J*_1_ = 12.8 Hz, *J*_2_ = 8.3 Hz, 1 H), 3.60 (s, 2 H), 7.21–7.26 (m, 1 H), 7.27–7.37 (m, 4 H), 7.60–7.67 (m, 2 H), 7.76 (dd, *J*_1_ = 8.6, *J*_2_ = 1.8 Hz, 1 H), 7.91–7.99 (m, 3 H), 8.35 (d, *J* = 1.0 Hz, 1 H). ^13^C NMR (CDCl_3_): δ 28.14, 35.03, 35.67, 53.49, 54.56, 57.96, 60.33, 122.70, 126.86, 127.47, 127.84, 128.18, 128.62, 128.65, 128.72, 129.15, 129.20, 132.16, 134.33, 134.69, 139.02. HRMS (ESI+): *m*/*z* calcd for C_23_H_27_N_2_O_2_S 395.1788; found 395.1782. UPLC purity 99%, at 254 nm (method B, *t*_R_ = 4.43 min). Chiral HPLC ee 100%, at 254 nm (method C, *t*_R_ = 12.84 min).

##### Synthesis of *tert*-Butyl (R)-3-((Naphthalene-2-sulfonamido)­methyl)­pyrrolidine-1-carboxylate (**91**)

Synthesized from compound **90** (5.000 g, 24.965 mmol, 1.0 equiv), naphthalene-2-sulfonyl chloride (5.656 g, 24.965 mmol, 1.0 equiv) and (CH_3_CH_2_)_3_N (3.480 mL, 24.965 mmol, 1.0 equiv) in CH_2_Cl_2_ (150 mL) via general procedure 1 to produce 9.719 g of crude compound **91** as a white solid. This product was used in the next step without further purification (99% yield). *R*_f_ = 0.36 (CH_2_Cl_2_/CH_3_OH, 30:1, v/v). HRMS (ESI+): *m*/*z* calcd for C_20_H_27_N_2_O_4_S 391.1686; found 391.1681.

##### Synthesis of *tert*-Butyl (R)-3-((*N*-Methylnaphthalene-2-sulfonamido)­methyl)­pyrrolidine-1-carboxylate (**92**)

Synthesized from compound **91** (4.862 g, 12.419 mmol, 1.0 equiv), K_2_CO_3_ (5.162 g, 37.257 mmol, 3.0 equiv) and CH_3_I (2.325 mL, 37.257 mmol, 3.0 equiv) in anhydrous DMF (49 mL) via general procedure 7 to produce 4.880 g of crude compound **92** as a white solid (97% yield). *R*_f_ = 0.33 (CH_2_Cl_2_/CH_3_OH, 30:1, v/v). HRMS (ESI+): *m*/*z* calcd for C_21_H_29_N_2_O_2_S 405.1843; found 405.1839.

##### Synthesis of (R)-*N*-Methyl-N-(pyrrolidin-3-ylmethyl)­naphthalene-2-sulfonamide hydrochloride (**93**)

Synthesized from compound **92** (4.880 g, 12.064 mmol, 1.0 equiv) and 2 M HCl solution in (CH_3_CH_2_)_2_O (18 mL, 36.191 mmol, 3.0 equiv) in CH_3_OH (98 mL) via general procedure 8 to produce 4.084 g of crude compound **93** as a white solid (99% yield). *R*_f_ = 0.06 (CH_2_Cl_2_/CH_3_OH, 20:1, v/v). HRMS (ESI+): *m*/*z* calcd for C_16_H_21_N_2_O_2_S 305.1318; found 305.1314.

##### Synthesis of (R)-N-((1-Benzylpyrrolidin-3-yl)­methyl)-*N*-methylnaphthalene-2-sulfonamide [**(*R*)-(−)-3**]

Synthesized from compound **93** (4.084 g, 12.008 mmol, 1.0 equiv), (CH_3_CH_2_)_3_N (3.347 mL, 24.016 mmol, 2.0 equiv) and benzyl bromide (1.426 mL, 12.008 mmol, 1.0 equiv) in anhydrous CH_2_Cl_2_ (150 mL) via general procedure 9. The crude product was purified by flash column chromatography using CH_2_Cl_2_/CH_3_OH (25:1, v/v) as the eluent to produce 3.078 g of compound **(** ***R*** **)-(−)-3** as a white solid (65% yield). *R*_f_ = 0.49 (CH_2_Cl_2_/CH_3_OH, 10:1, v/v). [α]_D_^25^–18.30 (c 0.5, CHCl_3_). ^1^H NMR (CDCl_3_): δ 1.51–1.59 (m, 1 H), 1.92–2.01 (m, 1 H), 2.29 (dd, *J*_1_ = 9.4; *J*_2_ = 6.2 Hz, 1 H), 2.41–2.50 (m, 1 H), 2.51–2.57 (m, 1 H), 2.60–2.65 (m, 1 H), 2.69–2.75 (m, 4 H), 2.98 (dd, *J*_1_ = 12.7; *J*_2_ = 7.3 Hz, 1 H), 3.06 (dd, *J*_1_ = 12.8 Hz, *J*_2_ = 8.3 Hz, 1 H), 3.60 (s, 2 H), 7.21–7.26 (m, 1 H), 7.27–7.37 (m, 4 H), 7.60–7.67 (m, 2 H), 7.76 (dd, *J*_1_ = 8.6, *J*_2_ = 1.8 Hz, 1 H), 7.91–7.99 (m, 3 H), 8.35 (d, *J* = 1.0 Hz, 1 H). ^13^C NMR (CDCl_3_): δ 28.14, 35.03, 35.67, 53.49, 54.56, 57.96, 60.33, 122.70, 126.87, 127.47, 127.84, 128.19, 128.63, 128.65, 128.73, 129.15, 129.21, 132.16, 134.32, 134.69, 139.01. HRMS (ESI+): *m*/*z* calcd for C_23_H_27_N_2_O_2_S 395.1788; found 395.1783. UPLC purity 99%, at 254 nm (method B, *t*_R_ = 4.44 min). Chiral HPLC ee 100%, at 254 nm (method C, *t*_R_ = 15.09 min).

##### Synthesis of (R)-N-((1-Benzylpyrrolidin-3-yl)­methyl)-*N*-methylnaphthalene-2-sulfonamide hydrochloride [**(*R*)-(−)-3** HCl]

Compound **(** ***R*** **)-(−)-3** (6.415 g, 16.260 mmol, 1.0 equiv) and a stirring bar were added into a 500 mL round-bottom flask. CH_3_OH (130 mL) was added, the resulting suspension stirred and agitated with a stream of argon for 15 min. The suspension was cooled to 0 °C and 2 M HCl solution in (CH_3_CH_2_)_2_O (8.943 mL, 17.886 mmol, 1.1 equiv) was added dropwise. The reaction mixture was allowed to warm to room temperature and stirred under argon for 24 h. During this time the suspension transformed into a solution. The solvent was evaporated to produce a white foam. (CH_3_CH_2_)_2_O (350 mL) and a stirring bar were added and the suspension stirred until (2 h) the foam transformed into a loose white solid. The stirring was stopped and the white solid was allowed to settle to the bottom of the flask. The supernatant was removed and (CH_3_CH_2_)_2_O (350 mL) was added and the resulting suspension stirred for 2 h. The stirring was stopped and the white solid was allowed to settle to the bottom of the flask. This process was repeated two more times. After the removal of the final supernatant the white solid was dried by evaporation to produce 6.951 g of **(** *R* **)-(−)-3 HCl** (99% yield). mp 181–183 °C. ^1^H NMR (CDCl_3_): δ 1.76–2.15 (m, 1 H), 2.20–2.48 (m, 1 H), 2.76 (s, 2 H), 2.84–3.33 (m, 6 H), 3.53–3.76 (m, 2 H), 4.20–4.30 (m, 2 H), 7.43–7.49 (m, 3 H), 7.60–7.79 (m, 5 H), 7.91–7.95 (m, 1 H), 7.97–8.04 (m, 2 H), 8.33–8.42 (m, 1 H), 12.91 (bs, 1 H). Chiral HPLC ee 100%, at 254 nm (method C, *t*_R_ = 14.72 min).

#### Synthesis and Characterization of Type C Inhibitors

##### Synthesis of N-(1-Benzylpyrrolidin-3-yl)­naphthalene-2-sulfonamide (**57**)

Synthesized from compound **62** (0.098 mL, 0.567 mmol, 1.0 equiv), naphthalene-2-sulfonyl chloride (0.129 g, 0.567 mmol, 1.0 equiv) and (CH_3_CH_2_)_3_N (0.079 mL, 0.567, 1.0 equiv) in CH_2_Cl_2_ (10 mL) via general procedure 1. The crude product was purified by flash column chromatography using CH_2_Cl_2_/CH_3_OH (40:1, v/v) as the eluent to produce 0.185 g of compound **57** as a brown oil (97% yield). *R*_f_ = 0.37 (CH_2_Cl_2_/CH_3_OH, 20:1, v/v). ^1^H NMR (CDCl_3_): δ 1.52–1.60 (m, 1 H), 2.03–2.12 (m, 1 H), 2.15–2.21 (m, 1 H), 2.33–2.41 (m, 2 H), 2.72 (sextet, *J* = 4.4 Hz, 1 H), 3.49 (dd, *J*_1_ = 32.1 Hz, *J*_2_ = 12.8 Hz, 2 H), 3.90 (bs, 1 H), 5.11 (bs, 1 H), 7.17–7.24 (m, 5 H), 7.58–7.66 (m, 2 H), 7.78 (dd, *J*_1_ = 8.7 Hz, *J*_2_ = 1.9 Hz, 1 H), 7.88–7.95 (m, 3 H), 8.42 (d, *J* = 1.4 Hz, 1 H). ^13^C NMR (CDCl_3_): δ 32.19, 51.69, 52.49, 59.51, 59.98, 122.18, 127.03, 127.37, 127.76, 128.11, 128.15, 128.58, 128.62, 129.08, 129.39, 131.99, 134.57, 137.45, 137.72. HRMS (ESI+): *m*/*z* calcd for C_21_H_23_N_2_O_2_S 367.1475; found 367.1472. UPLC purity 97%, at 254 nm (method A, *t*_R_ = 2.92 min).

##### Synthesis of Benzyl 3-(Methylamino)­pyrrolidine-1-carboxylate (**64**)

Synthesized from compound **63** (1.250 g, 5.702 mmol, 1.0 equiv), 2 M solution of CH_3_NH_2_ in THF (2.851 mL, 5.702 mmol, 1.0 equiv), NaBH­(OAc)_3_ (1.813 g, 8.553 mmol, 1.5. equiv) and AcOH (0.326 mL, 5.702 mmol, 1.0 equiv) in 1,2-dichloroethane (50 mL) via general procedure 2 to produce 1.249 g of compound **64** as a colorless oil (94% yield). *R*_f_ = 0.32 (CH_2_Cl_2_/CH_3_OH, 2:1, v/v).

##### Synthesis of Benzyl 3-(*N*-Methylnaphthalene-2-sulfonamido)­pyrrolidine-1-carboxylate (**68**)

Synthesized from compound **64** (1.249 g, 5.331 mmol, 1.0 equiv), naphthalene-2-sulfonyl chloride (1.208 g, 5.331 mmol, 1.0 equiv) and (CH_3_CH_2_)_3_N (0.743 mL, 5.331 mmol, 1.0 equiv) in CH_2_Cl_2_ (30 mL) via general procedure 1. The crude product was purified by flash column chromatography using CH_2_Cl_2_/CH_3_OH (60:1, v/v) as the eluent to produce 0.729 g of compound **68** as a colorless oil (32% yield). *R*_f_ = 0.36 (CH_2_Cl_2_/CH_3_OH, 40:1, v/v). IR (ATR): ^1^H NMR (CDCl_3_): δ 1.76–2.01 (m, 2 H), 2.81 (s, 3 H), 3.12–3.21 (m, 1 H), 3.27–3.34 (m, 1 H), 3.43–3.58 (m, 2 H), 4.60 (sextet, *J* = 7.5 Hz, 1 H), 5.08 (s, 2 H), 7.28–7.37 (m, 5 H), 7.65 (p, *J* = 7.3 Hz, 2 H), 7.76 (dd, *J*_1_ = 8.7 Hz, *J*_2_ = 1.3 Hz, 1 H), 7.93 (d, *J* = 7.8 Hz, 1 H), 7.96 (s, 1 H), 7.99 (s, 1 H), 8.38 (s, 1 H). ^13^C NMR (CDCl_3_): δ 27.00, 28.35, 29.25, 44.01, 44.34, 46.54, 46.83, 55.20, 55.93, 66.82, 122.09, 122.14, 127.63, 127.79, 127.87, 127.94, 128.38, 128.45, 128.83, 129.10, 129.56, 129.61, 132.10, 134.73, 135.41, 136.48, 154.47.^94^ HRMS (ESI+): *m*/*z* calcd for C_23_H_25_N_2_O_4_S 425.1530; found 425.1526. UPLC purity 98%, at 254 nm (method A, *t*_R_ = 4.42 min).

##### Synthesis of *N*-Methyl-N-(pyrrolidin-3-yl)­naphthalene-2-sulfonamide (**72**)

Synthesized from compound **68** (0.670 g, 1.578 mmol, 1.0 equiv), Pd/C (10 wt %) (0.140 g, 20% mass of compound **68**) and gaseous hydrogen in a mixture of CH_3_OH (20 mL) and THF (5 mL) via general procedure 3 to produce 0.361 g of compound **72** as a slightly golden oil (79% yield).

##### Synthesis of N-(1-Benzylpyrrolidin-3-yl)-*N*-methylnaphthalene-2-sulfonamide (**58**)

Synthesized from compound **72** (0.361 g, 1.243 mmol, 1.0 equiv), Cs_2_CO_3_ (0.405 g, 1.243 mmol, 1.0 equiv) and benzyl bromide (0.148 mL, 1.243 mmol, 1.0 equiv) in acetone (20 mL) via general procedure 4. The crude product was purified by flash column chromatography using CH_2_Cl_2_/CH_3_OH (60:1, v/v) as the eluent to produce 0.140 g of compound **58** as a golden oil (30% yield). *R*_f_ = 0.36 (CH_2_Cl_2_/CH_3_OH, 50:1, v/v). ^1^H NMR (CDCl_3_): δ 1.50–1.55 (m, 1 H), 1.89–1.98 (m, 1 H), 2.14 (q, *J* = 8.4 Hz, 1 H), 2.37 (dd, *J*_1_ = 10.3 Hz, *J*_2_ = 8.1 Hz, 1 H), 2.45 (dd, *J*_1_ = 10.5 Hz, *J*_2_ = 3.7 Hz, 1 H), 2.65–2.70 (m, 1 H), 2.86 (s, 3 H), 3.41 (d, *J* = 13.0 Hz, 1 H), 3.50 (d, *J* = 12.8 Hz, 1 H), 4.66–4.73 (m, 1 H), 7.18–7.23 (m, 5 H), 7.63 (pd, *J*_1_ = 7.1 Hz, *J*_2_ = 1.5 Hz, 2 H), 7.74 (dd, *J*_1_ = 8.6 Hz, *J*_2_ = 1.8 Hz, 1 H), 7.89–7.96 (m, 3 H), 8.35 (d, *J* = 1.2 Hz, 1 H). ^13^C NMR (CDCl_3_): δ 27.69, 29.40, 55.99, 56.64, 59.82, 65.19, 122.45, 126.89, 126.95, 127.43, 127.49, 127.83, 128.15, 128.39, 128.45, 128.58, 129.08, 129.24, 132.13, 134.62, 135.81, 138.45. HRMS (ESI+): *m*/*z* calcd for C_22_H_25_N_2_O_2_S 381.1631; found 381.1628. UPLC purity 96%, at 254 nm (method A, *t*_R_ = 3.18 min).

##### Synthesis of Benzyl 3-(Ethylamino)­pyrrolidine-1-carboxylate (**65**)

Synthesized from compound **63** (1.250 g, 5.702 mmol, 1.0 equiv), 2 M solution of CH_3_CH_2_NH_2_ in THF (2.851 mL, 5.702 mmol, 1.0 equiv), NaBH­(OAc)_3_ (1.813 g, 8.553 mmol, 1.5. equiv) and AcOH (0.326 mL, 5.702 mmol, 1.0 equiv) in 1,2-dichloroethane (50 mL) via general procedure 2 to produce 1.381 g of compound **65** as a slightly golden oil (98% yield). *R*_f_ = 0.52 (CH_2_Cl_2_/CH_3_OH, 2:1, v/v).

##### Synthesis of Benzyl 3-(N-Ethylnaphthalene-2-sulfonamido)­pyrrolidine-1-carboxylate (**69**)

Synthesized from compound **65** (1.381 g, 5.557 mmol, 1.0 equiv), naphthalene-2-sulfonyl chloride (1.260 g, 5.557 mmol, 1.0 equiv) and (CH_3_CH_2_)_3_N (0.775 mL, 5.557 mmol, 1.0 equiv) in CH_2_Cl_2_ (30 mL) via general procedure 1. The crude product was purified by flash column chromatography using CH_2_Cl_2_/CH_3_OH (100:1, v/v) as the eluent to produce 1.238 g of compound **69** as a slightly golden oil (53% yield). *R*_f_ = 0.38 (CH_2_Cl_2_/CH_3_OH, 50:1, v/v). ^1^H NMR (CDCl_3_): δ 1.30 (q, *J* = 7.2 Hz, 3 H), 1.80–2.10 (m, 2 H), 3.05–3.14 (m, 1 H), 3.17–3.34 (m, 3 H), 3.48–3.60 (m, 2 H), 4.46–4.58 (m, 1 H), 5.04–5.11 (m, 2 H), 7.28–7.36 (m, 5 H), 7.60–7.68 (m, 2 H), 7.77 (dd, *J*_1_ = 8.6 Hz, *J*_2_ = 1.7 Hz, 1 H), 7.91 (d, *J* = 7.9 Hz, 1 H), 7.95 (s, 1 H), 7.97 (s, 1 H), 8.41 (s, 1 H). ^13^C NMR (CDCl_3_): δ 17.37, 28.21, 29.46, 39.16, 43.84, 44.08, 47.35, 47.65, 55.70, 56.38, 66.89, 122.09, 122.15, 127.64, 127.90, 128.01, 128.41, 128.45, 128.83, 129.18, 129.58, 129.65, 132.16, 134.75, 136.57, 137.04, 137.09, 154.58, 154.62.^94^ HRMS (ESI+): *m*/*z* calcd for C_24_H_27_N_2_O_4_S 439.1686; found 439.1695. UPLC purity 99%, at 254 nm (method A, *t*_R_ = 4.57 min).

##### Synthesis of N-Ethyl-N-(pyrrolidin-3-yl)­naphthalene-2-sulfonamide (**73**)

Synthesized from compound **69** (1.243 g, 2.834 mmol, 1.0 equiv), Pd/C (10 wt %) (0.249 g, 20% mass of compound **69**) and gaseous hydrogen in a mixture of CH_3_OH (30 mL) and THF (5 mL) via general procedure 3 to produce 0.767 g of compound **73** as a slightly golden oil (89% yield).

##### Synthesis of N-(1-Benzylpyrrolidin-3-yl)-N-ethylnaphthalene-2-sulfonamide (**59**)

Synthesized from compound **73** (0.767 g, 2.520 mmol, 1.0 equiv), Cs_2_CO_3_ (0.821 g, 2.520 mmol, 1.0 equiv) and benzyl bromide (0.299 mL, 2.52 mmol, 1.0 equiv) in acetone (30 mL) via general procedure 4. The crude product was purified by flash column chromatography using CH_2_Cl_2_/CH_3_OH (50:1, v/v) as the eluent to produce 0.320 g of compound **59** as a slightly brown oil (32% yield). *R*_f_ = 0.30 (CH_2_Cl_2_/CH_3_OH, 50:1, v/v). ^1^H NMR (CDCl_3_): δ 1.33 (t, *J* = 7.0 Hz, 3 H), 1.49–1.57 (m, 1 H), 1.95–2.03 (m, 1 H), 2.17 (q, *J* = 8.3 Hz, 1 H), 2.39–2.49 (m, 2 H), 2.69 (td, *J*_1_ = 8.5 Hz, *J*_2_ = 3.2 Hz, 1 H), 3.30–3.53 (m, 4 H), 4.53–4.60 (m, 1 H), 7.18–7.25 (m, 5 H), 7.58–7.66 (m, 2 H), 7.76 (dd, *J*_1_ = 8.7 Hz, *J*_2_ = 1.8 Hz, 1 H), 7.88–7.96 (m, 3 H), 8.38 (d, *J* = 1.3 Hz, 1 H). ^13^C NMR (CDCl_3_): δ 17.61, 29.33, 39.44, 52.95, 56.66, 57.75, 59.84, 122.41, 126.90, 127.33, 127.73, 128.08, 128.23, 128.37, 128.45, 128.99, 129.15, 132.04, 134.49, 137.04, 138.40. HRMS (ESI+): *m*/*z* calcd for C_23_H_27_N_2_O_2_S 395.1788; found 395.1785. UPLC purity 98%, at 254 nm (method A, *t*_R_ = 3.39 min).

##### Synthesis of Benzyl 3-((2-Methoxyethyl)­amino)­pyrrolidine-1-carboxylate (**66**)

Synthesized from compound **63** (1.250 g, 5.702 mmol, 1.0 equiv), 2-methoxyethan-1-amine (0.490 mL, 5.702 mmol, 1.0 equiv), NaBH­(OAc)_3_ (1.813 g, 8.552 mmol, 1.5 equiv) and AcOH (0.326 mL, 5.702 mmol, 1.0 equiv) in 1,2-dichloroethane (50 mL) via general procedure 2 to produce 1.487 g of compound **66** as a colorless oil (94% yield). *R*_f_ = 0.33 (CH_2_Cl_2_/CH_3_OH (9:1, v/v).

##### Synthesis of Benzyl 3-(N-(2-Methoxyethyl)­naphthalene-2-sulfonamido)­pyrrolidine-1-carboxylate (**70**)

Synthesized from compound **66** (1.487 g, 5.324 mmol, 1.0 equiv), naphthalene-2-sulfonyl chloride (1.211 g, 5.324 mmol, 1.0 equiv) and (CH_3_CH_2_)_3_N (0.746 mL, 5.324 mmol, 1.0 equiv) in CH_2_Cl_2_ (30 mL) via general procedure 1. The crude product was purified by flash column chromatography using CH_2_Cl_2_/CH_3_OH (50:1, v/v) as the eluent to produce 1.217 g of compound **70** as a slightly golden oil (49% yield). *R*_f_ = 0.31 (CH_2_Cl_2_/CH_3_OH, 50:1, v/v). ^1^H NMR (CDCl_3_): δ 1.88–2.03 (m, 2 H), 3.08–3.40 (m, 7 H), 3.46–3.64 (m, 4 H), 4.48 (septet, *J* = 8.6 Hz, 1 H), 5.08 (d, *J* = 8.7 Hz, 2 H), 7.28–7.32 (m, 5 H), 7.65 (p, *J* = 7.7 Hz, 2 H), 7.78 (dd, *J*_1_ = 8.7 Hz, *J*_2_ = 1.8 Hz, 1 H), 7.92 (d, *J* = 8.1 Hz, 1 H), 7.97 (d, *J* = 8.4 Hz, 2 H), 8.41 (s, 1 H). ^13^C NMR (CDCl_3_): δ 27.99, 29.22, 43.79, 44.04, 47.38, 47.59, 55.93, 56.63, 58.90, 66.81, 72.40, 122.09, 122.14, 127.68, 127.78, 127.86, 127.91, 128.42, 128.50, 128.90, 129.18, 129.61, 129.69, 132.13, 134.80, 136.53, 136.61, 154.51, 154.61.^94^ HRMS (ESI+): *m*/*z* calcd for C_25_H_29_N_2_O_5_S 469.1792; found 469.1792. UPLC purity 99%, at 254 nm (method A, *t*_R_ = 4.51 min).

##### Synthesis of N-(2-Methoxyethyl)-N-(pyrrolidin-3-yl)­naphthalene-2-sulfonamide (**74**)

Synthesized from compound **70** (1.182 g, 2.523 mmol, 1.0 equiv), Pd/C (10 wt %) (0.236 g, 20% mass of compound **70**) and gaseous hydrogen in CH_3_OH (30 mL) via general procedure 3 to produce 0.666 g of compound **74** as a colorless oil (79% yield).

##### Synthesis of N-(1-Benzylpyrrolidin-3-yl)-N-(2-methoxyethyl)­naphthalene-2-sulfonamide (**60**)

Synthesized from compound **74** (0.666 g, 1.991 mmol, 1.0 equiv), Cs_2_CO_3_ (0.649 g, 1.991 mmol, 1.0 equiv) and benzyl bromide (0.237 mL, 1.991 mmol, 1.0 equiv) in acetone (20 mL) via general procedure 4. The crude product was purified by flash column chromatography using CH_2_Cl_2_/CH_3_OH (40:1, v/v) as the eluent to produce 0.310 g of compound **60** as a brown oil (37% yield). *R*_f_ = 0.33 (CH_2_Cl_2_/CH_3_OH, 40:1, v/v). ^1^H NMR (CDCl_3_): δ 1.52–1.58 (m, 1 H), 1.93–2.01 (m, 1 H), 2.08–2.15 (m, 1 H), 2.36 (t, *J* = 8.3 Hz, 1 H), 2.49 (dd, *J*_1_ = 10.4 Hz, *J*_2_ = 3.5 Hz, 1 H), 2.71 (td, *J*_1_ = 8.8 Hz, *J*_2_ = 3.2 Hz, 1 H), 3.35 (s, 3 H), 3.37–3.53 (m, 4 H), 3.59–3.72 (m, 2 H), 4.51–4.57 (m, 1 H), 7.17–7.25 (m, 5 H), 7.63 (qd, *J*_1_ = 7.2 Hz, *J*_2_ = 1.5 Hz, 2 H), 7.77 (dd, *J*_1_ = 8.7 Hz, *J*_2_ = 1.8 Hz, 1 H), 7.92 (q, *J* = 9.0 Hz, 3 H), 8.38 (d, *J* = 1.3 Hz, 1 H). ^13^C NMR (CDCl_3_): δ 29.20, 43.67, 53.04, 56.75, 57.85, 58.73, 59.82, 72.50, 122.48, 126.89, 127.41, 127.77, 128.08, 128.43, 128.58, 129.04, 129.22, 132.06, 134.59, 136.49, 138.38. HRMS (ESI+): *m*/*z* calcd for C_24_H_28_N_2_O_3_S 425.1893; found 425.1888. UPLC purity 97%, at 254 nm (method A, *t*_R_ = 3.42 min).

##### Synthesis of Benzyl 3-((3-Methoxypropyl)­amino)­pyrrolidine-1-carboxylate (**67**)

Synthesized from compound **65** (1.250 g, 5.702 mmol, 1.0 equiv), 3-methoxypropan-1-amine (0.582 mL, 5.702 mmol, 1.0 equiv), NaBH­(OAc)_3_ (1.813 g, 8.552 mmol, 1.5 equiv) and AcOH (0.326 mL, 5.702 mmol, 1.0 equiv) in 1,2-dichloroethane (50 mL) via general procedure 2 to produce 1.528 g of compound **67** as a colorless oil (92% yield). *R*_f_ = 0.62 (CH_2_Cl_2_/CH_3_OH (4:1, v/v).

##### Synthesis of Benzyl 3-(N-(3-Methoxypropyl)­naphthalene-2-sulfonamido)­pyrrolidine-1-carboxylate (**71**)

Synthesized from compound **67** (1.528 g, 5.227 mmol, 1.0 equiv), naphthalene-2-sulfonyl chloride (1.185 g, 5.227 mmol, 1.0 equiv) and (CH_3_CH_2_)_3_N (0.729 mL, 5.227 mmol, 1.0 equiv) in CH_2_Cl_2_ (30 mL) via general procedure 1. The crude product was purified by flash column chromatography using CH_2_Cl_2_/CH_3_OH (40:1, v/v) as the eluent to produce 1.216 g of compound **71** as a slightly golden oil (48% yield). *R*_f_ = 0.31 (CH_2_Cl_2_/CH_3_OH, 40:1, v/v). ^1^H NMR (CDCl_3_): δ 1.80–2.02 (m, 4 H), 3.02–3.12 (m, 1 H), 3.21–3.30 (m, 6 H), 3.36–3.55 (m, 4 H), 4.51 (septet, *J* = 8.4 Hz, 1 H), 5.03–5.10 (m, 2 H), 7.28–7.32 (m, 5 H), 7.63–7.68 (m, 2 H), 7.78 (d, *J* = 8.7 Hz, 1 H), 7.92 (d, *J* = 7.7 Hz, 1 H), 7.96 (d, *J* = 8.6 Hz, 2 H), 8.40 (s, 1 H). ^13^C NMR (CDCl_3_): δ 27.74, 29.07, 31.77, 31.88, 41.75, 43.74, 43.79, 47.05, 47.15, 55.92, 56.62, 58.55, 66.83, 69.51, 69.60, 122.10, 122.19, 127.65, 127.85, 127.88, 127.96, 128.41, 128.49, 128.86, 129.18, 129.57, 129.67, 132.13, 134.76, 136.54, 136.59, 136.62, 136.67, 154.49, 154.59. HRMS (ESI+): *m*/*z* calcd for C_26_H_31_N_2_O_5_S 483.1948; found 483.1947. UPLC purity 99%, at 254 nm (method A, *t*_R_ = 4.58 min).

##### Synthesis of N-(3-Methoxypropyl)-N-(pyrrolidin-3-yl)­naphthalene-2-sulfonamide (**75**)

Synthesized from compound **71** (1.178 g, 2.441 mmol, 1.0 equiv), Pd/C (10 wt %) (0.236 g, 20% mass of compound **71**) and gaseous hydrogen in CH_3_OH (30 mL) via general procedure 3 to produce 0.677 g of compound **75** as a slightly golden oil (80% yield).

##### Synthesis of N-(1-Benzylpyrrolidin-3-yl)-N-(3-methoxypropyl)­naphthalene-2-sulfonamide (**61**)

Synthesized from compound **75** (0.677 g, 1.942 mmol, 1.0 equiv), Cs_2_CO_3_ (0.633 g, 1.942 mmol, 1.0 equiv) and benzyl bromide (0.231 mL, 1.942 mmol, 1.0 equiv) in acetone (20 mL) via general procedure 4. The crude product was purified by flash column chromatography using CH_2_Cl_2_/CH_3_OH (50:1, v/v) as the eluent to produce 0.436 g of compound **61** as a slightly brown oil (51% yield). *R*_f_ = 0.32 (CH_2_Cl_2_/CH_3_OH, 40:1, v/v). ^1^H NMR (CDCl_3_): δ 1.47–1.56 (m, 1 H), 1.90–1.99 (m, 1 H), 2.00–2.11 (m, 2 H), 2.13–2.19 (m, 1 H), 2.40–2.48 (m, 2 H), 2.65 (td, *J*_1_ = 8.8 Hz, *J*_2_ = 3.8 Hz, 1 H), 3.29–3.39 (m, 5 H), 3.41–3.49 (m, 4 H), 4.54–4.61 (m, 1 H), 7.17–7.24 (m, 5 H), 7.59–7.66 (m, 2 H), 7.77 (dd, *J*_1_ = 8.6 Hz, *J*_2_ = 1.9 Hz, 1 H), 7.88–7.96 (m, 3 H), 8.38 (d, *J* = 1.3 Hz, 1 H). ^13^C NMR (CDCl_3_): δ 28.98, 31.79, 42.15, 52.84, 56.83, 57.50, 58.33, 59.74, 69.98, 122.44, 126.86, 127.33, 127.71, 128.03, 128.32, 128.39, 128.48, 128.98, 129.14, 132.01, 134.49, 136.64, 138.34. HRMS (ESI+): *m*/*z* calcd for C_25_H_31_N_2_O_3_S 439.2050; found 439.2047. UPLC purity 97%, at 254 nm (method A, *t*_R_ = 3.47 min).

#### Synthesis and Characterization of Type D Inhibitors

##### Synthesis of 1-Benzylimidazolidine (**79**)

Compound **80** (1 mL, 6.657 mmol, 1.0 equiv) was added into a dry 50 mL round-bottom flask equipped with a magnetic stirring bar. Dry CH_3_OH (25 mL) was added with a double tipped needle. The resulting solution was stirred and paraformaldehyde (0.300 g, 9.986 mmol, 1.5 equiv) was added, followed by 4 Å molecular sieves (2 g). The suspension was stirred at room temperature under argon for 20 h. The suspension was filtered with suction and the filtrate evaporated to produce 1.051 g of crude compound **79** as a colorless oil (97% yield). *R*_f_ = 0.52 (CH_2_Cl_2_/CH_3_OH, 10:1, v/v). This product was used in the next step without further purification.

##### Synthesis of 1-Benzyl-3-(naphthalen-2-ylsulfonyl)­imidazolidine (**76**)

Synthesized from compound **79** (0.102 g, 0.629 mmol, 1.0 equiv), naphthalene-2-sulfonyl chloride (0.143 g, 0.629 mmol, 1.0 equiv) and (CH_3_CH_2_)_3_N (0.088 mL, 0.629 mmol, 1.0 equiv) in CH_2_Cl_2_ (10 mL) via general procedure 1. The crude product was purified by flash column chromatography using CH_2_Cl_2_/CH_3_OH (100:1, v/v) as the eluent to produce 0.132 g of compound **76** as a white solid (59% yield). *R*_f_ = 0.34 (CH_2_Cl_2_/CH_3_OH, 100:1, v/v). ^1^H NMR (CDCl_3_): δ 2.83 (t, *J* = 6.5 Hz, 2 H), 3.45–3.48 (m, 4 H), 4.08 (s, 2 H), 7.07–7.09 (m, 2 H), 7.21–7.27 (m, 3 H), 7.65–7.73 (m, 2 H), 7.84 (dd, *J*_1_ = 8.6 Hz, *J*_2_ = 1.8 Hz, 1 H), 7.97–8.03 (m, 3 H), 8.41–8.42 (m, 1 H). ^13^C NMR (CDCl_3_): δ 45.64, 52.40, 57.13, 69.86, 122.85, 127.26, 127.40, 127.78, 128.22, 128.67, 128.75, 129.13, 129.19, 132.05, 134.11, 134.76, 137.44. HRMS (ESI+): *m*/*z* calcd for C_20_H_21_N_2_O_2_S 353.1318; found 353.1315. UPLC purity 98%, at 254 nm (method A, *t*_R_ = 2.84 min).

##### Synthesis of 1-Benzyl-3-(naphthalen-2-ylsulfonyl)­hexahydropyrimidine (**77**)

Synthesized from compound **81** (0.050 g, 0.284 mmol, 1.0 equiv), naphthalene-2-sulfonyl chloride (0.064 g, 0.284 mmol, 1.0 equiv) and (CH_3_CH_2_)_3_N (0.040 mL, 0.284 mmol, 1.0 equiv) in CH_2_Cl_2_ (5 mL) via general procedure 1. The crude product was purified by flash column chromatography using CH_2_Cl_2_/CH_3_OH (100:1, v/v) as the eluent to produce 0.071 g of compound **77** as a white solid (68% yield). *R*_f_ = 0.32 (CH_2_Cl_2_/CH_3_OH, 100:1, v/v). ^1^H NMR (CDCl_3_): δ 1.62 (quintet, *J* = 5.6 Hz, 2 H), 2.64 (t, *J* = 5.6 Hz, 2 H), 3.34 (t, *J* = 5.7 Hz, 2 H), 3.73 (s, 2 H), 4.09 (s, 2 H), 7.27–7.31 (m, 5 H), 7.60–7.68 (m, 2 H), 7.77 (dd, *J*_1_ = 8.7 Hz, *J*_2_ = 1.8 Hz, 1 H), 7.93 (d, *J* = 7.8 Hz, 1 H), 7.95 (s, 1 H), 7.98 (s, 1 H), 8.34 (d, *J* = 1.1 Hz, 1 H). ^13^C NMR (CDCl_3_): δ 21.00, 45.89, 50.20, 56.59, 67.40, 122.80, 127.21, 127.40, 127.83, 128.26, 128.52, 128.64, 128.93, 129.10, 129.13, 132.14, 134.70, 135.41, 137.78. HRMS (ESI+): *m*/*z* calcd for C_21_H_23_N_2_O_2_S 367.1475; found 367.1473. UPLC purity 97%, at 254 nm (method A, *t*_R_ = 3.22 min).

##### Synthesis of *tert*-Butyl 4-(Naphthalen-2-ylsulfonyl)­piperazine-1-carboxylate (**82**)

Synthesized from compound **83** (0.104 g, 0.558 mmol, 1.0 equiv), naphthalene-2-sulfonyl chloride (0.127 g, 0.558 mmol, 1.0 equiv) and (CH_3_CH_2_)_3_N (0.078 mL, 0.558 mmol, 1.0 equiv) in CH_2_Cl_2_ (10 mL) via general procedure 1. The crude product was purified by flash column chromatography using EtOAc/*n*-hex (1:3, v/v) as the eluent to produce 0.190 g of compound **82** as a white solid (90% yield). *R*_f_ = 0.26 (EtOAc/*n*-hex, 1:3, v/v). ^1^H NMR (CDCl_3_): δ 1.38 (s, 9 H), 3.04 (bt, J = 4.5 Hz, 4 H), 3.52 (t, *J* = 5.1 Hz, 4 H), 7.61–7.69 (m, 2 H), 7.74 (dd, *J*_1_ = 8.7 Hz, *J*_2_ = 1.9 Hz, 1 H), 7.93 (d, *J* = 7.7 Hz, 1 H), 7.99 (d, *J* = 8.3 Hz, 2 H), 8.33 (d, *J* = 1.6 Hz, 1 H). ^13^C NMR (CDCl_3_): δ 28.25, 43.03, 45.95, 80.35, 122.84, 127.69, 127.94, 128.99, 129.12, 129.23, 129.35, 132.19, 132.63, 134.95, 154.07. HRMS (ESI+): *m*/*z* calcd for C_19_H_24_N_2_O_4_ NaS 399.1349; found 399.1348. UPLC purity 99%, at 254 nm (method A, *t*_R_ = 4.47 min).

##### Synthesis of 1-(Naphthalen-2-ylsulfonyl)­piperazine Hydrochloride (**84**)

Synthesized for compound **82** (0.175 g, 0.465 mmol, 1.0 equiv) and 2 M solution of HCl in Et_2_O (4.650 mL, 9.300 mmol, 20 equiv) in CH_3_OH (10 mL) via general procedure 8 to produce 0.141 g of crude compound **84** as an orange solid (97% yield).

##### Synthesis of 1-Benzyl-4-(naphthalen-2-ylsulfonyl)­piperazine (**78**)

Synthesized from compound **84** (0.141 g, 0.451 mmol, 1.0 equiv), Cs_2_CO_3_ (0.294 g, 0.902 mmol, 2.0 equiv) and benzyl bromide (0.054 mL, 0.451 mmol, 1.0 equiv) in acetone (10 mL) via general procedure 4. The crude product was purified by flash column chromatography using CH_2_Cl_2_/CH_3_OH (30:1, v/v) as the eluent to produce 0.114 g of compound **78** as a white solid (85% yield). *R*_f_ = 0.56 (EtOAc/*n*-hex, 1:1, v/v). ^1^H NMR (CDCl_3_): δ 2.53 (t, *J* = 4.8 Hz, 4 H), 3.09 (bs, 4 H), 3.47 (s, 2 H), 7.19–7.28 (m, 5 H), 7.60–7.68 (m, 2 H), 7.74 (dd, *J*_1_ = 8.6 Hz, *J*_2_ = 1.7 Hz, 1 H), 7.93 (d, *J* = 7.7 Hz, 1 H), 7.97 (d, *J* = 8.2 Hz, 2 H), 8.32 (d, *J* = 1.0 Hz, 1 H). ^13^C NMR (CDCl_3_): δ 46.07, 51.94, 62.38, 122.98, 127.13, 127.79, 128.16, 128.71, 128.89, 128.95, 129.04, 129.06, 132.06, 132.50, 134.74, 137.32. HRMS (ESI+): *m*/*z* calcd for C_21_H_23_N_2_O_2_S 367.1475; found 367.1474. UPLC purity 98%, at 254 nm (method A, *t*_R_ = 3.05 min).

#### In Vitro ChE Inhibition Assay

The inhibitory activities of the compounds against ChEs were assessed using Ellman’s method^56,30^ as reported previously. Ellman’s reagent (5,5′-Dithiobis­(2-nitrobenzoic acid), DTNB), acetylthiocholine iodide (ATCI) and butyrylthiocholine iodide (BTCI) were purchased from TCI Europe and Sigma-Aldrich. Recombinant hAChE (14.4 mg/mL) and recombinant hBChE (21 mg/mL) were diluted in 0.1 M Na-phosphate (pH = 8.0). For inhibitory screening, stock solutions of the test compounds were prepared in DMSO (10 mM), and a serial dilution of the compounds was used for the dose–response determination. The assay was performed at room temperature (18–25 °C) on 96-well flat-bottom microtiter plates in 0.1 M Na-phosphate (pH = 8.0) (final volume, 300 μL), containing DTNB (final concentration, 370 μM), ATCI or BTCI for hAChE and hBChE, respectively (final concentration, 500 μM), with final concentration of DMSO 1% [v/v]. The increase in absorbance at 412 nm was followed for 2 min using a microplate reader (Synergy HT; BioTek Instruments, Inc., USA). The initial velocities in the absence (*v*_0_) and presence of the compound (*v*_*i*_) were calculated from the slope of the linear trend obtained, with each measurement carried out in triplicate. The inhibitory potencies are expressed as the residual activities (RA = *v_i_*/(*v*_o_) × 100%). The IC_50_ values were calculated using the 4-parameter logistic function in GraphPad Prism software v 10.0 (GraphPad Software, USA).

#### Absolute Configuration of Compounds **(*S*)-(+)-3** and **(*R*)-(−)-3**

##### Crystallographic Data Collection and Refinement of the Structures

X-ray diffraction data was collected on an Oxford Diffraction SuperNova diffractometer with Mo/Cu microfocus X-ray source with mirror optics and an Atlas detector at 150.00(10) K. The structures were solved in Olex2 ^90^ graphical user interface by direct methods implemented in SHELXT^91^ and refined by a full-matrix least-squares procedure based on *F* ^2^ using SHELXL.^92^ All non-hydrogen atoms were refined anisotropically. The hydrogen atoms were placed at calculated positions and treated using appropriate riding models. Figures were prepared using Mercury.^93^ The crystal structures have been submitted to the CCDC and allocated the deposition numbers 2423398 [**(** ***S*** **)-(+)-3**] and 2423399 [**(** ***R*** **)-(−)-3**]. Additional details on structural properties and refinement details are given in Table S2.

#### hBChE Inhibition and Kinetic Studies

The reaction mechanism of hBChE inhibition by compounds **2**, **(** ***S*** **)-(+)-3** and **(** ***R*** **)-(−)-3** was tested as previously described.^30^ Briefly, the time course of butyrylthiocholine (BTCh) hydrolysis by hBChE was followed according to the Ellman’s method^56^ on a PerkinElmer Lambda 45 UV/vis spectrophotometer. Buffer solutions (25 mM phosphate buffer, pH 7.0) of all components were mixed in a cuvette and the resulting solution contained 50 μM BTCh, 1 mM DTNB and 0.5 nM purified hBChE or full plasma hBChE of similar concentration. Additionally, each of the synthesized compounds was added in final concentrations between 10 and 200 nM. The absorbance was followed at 412 nm immediately after the addition of enzyme. Technically, for the final solution in the cuvette, 300 μL of buffer solution containing 0.1 mM BTCh and 2 mM DTNB was mixed with buffer containing the selected concentration of inhibitor (zero to 200 nM) in the volume achieving 300 μL upon ultimate addition of enzyme (typically: 295 μL of buffer and 5 μL of enzyme stock solution). The absorbance was followed in 0.5 mm path length cuvette for 10 min. The conditions were selected so that the curve in the absence of inhibitor always achieved the plateau, indicating the complete substrate hydrolysis. The measured progress curves were analyzed by the ENZO application.^59^

#### X-ray Crystal Structures of hBChE in Complex with Enantiomers **(*S*)-(+)-3** and **(*R*)-(−)-3**

##### Crystallization

Recombinant hBChE was produced in Chinese hamster ovary (CHO) cells^95^ and purified according to the protocol described earlier for hBuChE produced in insect cells.^96^ Crystals were obtained by vapor diffusion using the hanging drop method at 293 K and 0.1 M MES pH 6.5, 2.15 M (NH_4_)_2_SO_4_ as crystallization buffer. Ligands were initially solubilized in 100% methanol (0.1 M) and protein–ligand complexes were obtained after crystal soaking with 5 mM compounds **(** ***S*** **)-(+)-3** or **(** ***R*** **)-(−)-3** in crystallization buffer. Crystals were cryo-protected in a solution of 0.1 M MES [2-(*N*-morpholino)­ethanesulfonic acid] pH 6.5, 2.15 M (NH_4_)_2_SO_4_, 20% glycerol, before flash cooling into liquid nitrogen.

##### Structure Determination

X-ray diffraction data were collected at the BM07 beamline (FIP2–French Beamline for Investigation of Proteins) of the European Synchrotron Radiation Facility (Grenoble, France) at 100 K. Recorded images were processed with XDSME.^97^ Phases were determined by molecular replacement using the hBChE X-ray structure (PDB entry 1P0I) devoid of any ligand, glycans or H_2_O molecules with the Phaser program of the Phenix software suite.^98^ Each model was obtained by iterative cycles of building using *Coot* ^99^ and refinement using Phenix.refine. Ligand geometry restraints were generated with Phenix eLBOW^100^ using the semiempirical quantum mechanical method (AM1). HBChE structures in complex with compound **(** ***S*** **)-(+)-3** and compound **(** ***R*** **)-(−)-3** were respectively deposited into the Protein Data Bank under accession numbers 9I02.pdb and 9I03.pdb. Data collection and refinement statistics of hBChE in complex with compound **(** ***S*** **)-(+)-3** and compound **(** ***R*** **)-(−)-3** are shown in Table S3.

#### Cell-Based Assays

##### Cell Culture and Treatments

The human hepatocellular carcinoma HepG2 and human neuroblastoma SH-SY5Y cell lines were purchased from American Type Culture Collection (Manassas, VA, USA). Cells were cultured in Advanced Dulbecco’s modified Eagle’s medium (Gibco, Thermo Fisher Scientific, Waltham, MA, USA) supplemented with 10% fetal bovine serum (FBS, Gibco), 2 mM l-glutamine, 50 U/mL penicillin and 50 μg/mL streptomycin (Sigma, St. Louis, MO, USA) in a humidified atmosphere of 95% air and 5% CO_2_ at 37 °C, and grown to 80% confluence. Prior to cell treatment, complete medium was replaced with reduced-serum medium (i.e., with 2% FBS). Compounds were prepared as a stock solution of 50 mM in DMSO and were used at concentrations of 1 to 100 μM. In case of cytotoxic stimuli, the peptide Aβ_1–42_ (Merck Millipore, Darmstadt, Germany) was dissolved in DMSO at a concentration of 1 mM, and was used at concentration 5 μM.

##### MTS Assay

HepG2 and SH-SY5Y cells were seeded in 96-well plates (1 × 10^4^/well) and assessed by MTS ([3-(4,5-dimethylthiazol-2-yl)-5-(3-carboxymethoxyphenyl)-2-(4-sulfophenyl)-2*H* tetrazolium, inner salt) assay for their response to hBChE inhibitors. Cells were treated with increasing concentrations of compounds (1–100 μM) in reduced-serum medium, and metabolic activity was assessed after 48 h using the CellTiter 96 Aqueous One Solution Cell Proliferation Assay (Promega, Madison, WI, USA), in accordance with the manufacturer’s instructions. Absorbance was measured with an automatic microplate reader (Tecan Safire,^2^ Switzerland) at a wavelength of 492 nm. Results are presented as a percentage of the control (DMSO).

##### Neuroprotection Assay

The neuroprotective effect of compounds **2**, **(** ***S*** **)-(+)-3** and **(** ***R*** **)-(−)-3** on cytotoxic effect of Aβ_1–42_ was determined with the fluorescent intercalator 7-AAD assessed by flow cytometry. Prior to cell treatment, the peptide Aβ_1–42_ was aggregated at concentration of 100 μM for 24 h at 37 °C to obtain fibrils. SH-Y5Y cells were seeded in 24-well culture plates (2 × 104/well) and next day treated with preaggregated Aβ_1–42_ in the absence or presence of compounds **2**, **(** ***S*** **)-(+)-3** and **(** ***R*** **)-(−)-3** at concentrations 2.5 and 5 μM. After 48 h treatment, cells were harvested and washed with cold PBS, and labeled with 7AAD (2 μg/mL; Sigma-Aldrich) for 10 min at room temperature. Cells were then analyzed for cytotoxicity by flow cytometry on Attune NxT flow cytometer (Thermo Fisher Scientific). The percentage of 7AAD positive (7AADpos) cells was evaluated using FlowJo software (FlowJo, LLC, Ashland, OR, USA) and recorded as relative to control cells.

#### Assay for Determining the Solubility of Compounds 2 HCl and **(*R*)-(−)-3** HCl

The solubility of compounds **2 HCl** and **(** ***R*** **)-(−)-3 HCl** were determined by a miniaturized shake-flask technique. For each measurement the 15 mg of the tested compound was placed in a 1.5 mL microtube (Eppendorf Safe-Lock) and approximately 170 mg of 0.5 mm glass beads was added before the addition of 0.25 mL of the aqueous dissolution medium. The dissolution media used for these measurements were: purified water, unbuffered saline, phosphate buffered salines at pH 6.8 and 7.4 (1.19 g Na_2_HPO_4_ × 12H_2_O, 0.095 g KH_2_PO_4_ and 4.00 g NaCl with H_2_O to 500 mL and pH adjusted by 1 M aqueous HCl solution or 1 M aqueous NaOH solution), acetate buffer at pH 4.5 (5.98 g CH_3_COONa × 3H_2_O and 3.2 mL of glacial CH_3_COOH with H_2_O to 2 L), 0.1 M HCl and FaSSGF (fasted state simulated gastric fluid from 2 g NaCl, 80 mL 1 M aqueous HCl solution and H_2_O to 1 L). The mixing was initiated by homogenization on a Bullet Blender 24 (Next Advance, USA) at speed setting ≫ 3 ≪ for 3 min and continued on an orbital thermostated shaker HeatMix (Domel, Slovenia) at 1200 rpm and 25 °C for 24 h. After centrifugation at 15,000 rcf and 25 °C on the 5425R Eppendorf centrifuge the supernatants were diluted 100-fold by 10% CH_3_CN in H_2_O and analyzed by HPLC. All measurements were made in triplicates. The HPLC system used for sample analysis was an Agilent 1100 with a degasser, binary pump, well plate autosampler, column thermostat and a diode-array detector. The mobile phase was 0.5% phosphoric acid set to pH 3.0 with ammonia flowing at 2 mL/min and CH_3_CN (37 and 34% for compounds **2** and **(** ***R*** **)-(−)-3**, respectively to result in retention times of 1.0 min for both compounds), the column was a Phenomenex, Kinetex 2.6um XB-C18, 50 × 4.6 mm working at 45 °C. The injection volume was 1 μL and the detection wavelength was 231 nm.

#### Assay for Determining Permeability in Caco-2 Cells

Caco-2 cells were obtained from American Tissue Culture Collection (ATCC) HTB.37 and were used in the reported experiment at passage No. 30 with regular cell line authentication and mycoplasma testing (Eurofins Gemonics Europe). They were grown on Millicell culture insert plates with a PET membrane (surface area, 0.7 cm^2^; pore size, 1.0 μm). 60,000 cells/filter membrane were used for seeding and the growth medium was changed every 2 days. At day 15, transepithelial electrical resistance (TEER) was measured for each filter with Caco-2 cell monolayers and was in the range of 350–600 Ω × cm^2^. On day 21 the TEER values were measured again and were in the range of 350–600 Ω × cm^2^ before and after the experiment for the determination of permeability. *ABCB1* activity was confirmed with Rhodamine123 (Sigma-Aldrich, Germany) a marker substrate of *ABCB1*, which had an efflux ratio of 12.8 [*P*_app(B–A)_ was 69 nm/s and *P*_app(A–B)_ was 5.4 nm/s) within the same experiment and fluorescein was used as an internal low permeability marker to indicate potential outliers or damaged cell monolayers. Fluorescence measurements with excitation at 485 nm and emission at 520 nm were used to quantify Rhodamine123 and fluorescein in samples taken as described later for compounds **2** and **(** ***R*** **)-(−)-3**. The samples were acidified by dilution with the same volume of 0.01 M aqueous HCl solution before fluorescence measurements of Rhodamine 123 or alkalized with the same volume of 0.01 M NaOH for fluorescein.

Ringer buffer with 10 mM d-glucose or 10 mM mannitol on apical and basolateral side of the tissue, respectively, was used as an incubation saline. The cell monolayers were kept at 37 °C in a carbogen (95% O_2_ and 5% CO_2_) atmosphere during the experiment. It was started by the addition of stock solutions in the donor compartments to provide final 100 μM donor concentration of tested compounds and 20 μM for fluorescein and rhodamine123. The stock solutions were added either to the apical or to the basolateral compartment to obtain bidirectional permeability measurements. Seven samples were withdrawn in 20 min intervals. Each time 160 μL was taken from the 800 μL basolateral acceptor compartment and 80 μL from the 400 μL apical acceptor compartment. These volumes were immediately replaced by the appropriate fresh incubation saline. The concentrations of tested compounds in the samples were determined by HPLC-UV immediately after the experiment. The analysis was performed on an Agilent 1100 system (degasser, binary pump, well-plate sampler, column thermostate and a diode-array detector) using a Phenomenex Kinetex XB-C18 column (4.6 × 50 mm, 2.6 μm) at 55 °C and a mobile phase consisting of A: 0.5% ammonium phosphate at pH 3.0 and B: CH_3_CN; with a flow of 2.0 mL/min (0.0 min, 20% B; 1.0 min, 35% B; 3.5 min, 55% B). The detection wavelength was 231 nm and the retention time was 2.16 min for compound **(** ***R*** **)-(−)-3** and 2.48 min for compound **2**. All apparent permeability coefficients were calculated as described previously.^63^

#### In Vivo Brain–Plasma Distribution Assay

##### Animals and Housing Conditions

Mice used in this assay were around 3 months old female and male wild-type Balb/c mice bred in the Laboratory of Animal Genomics at the Faculty of Veterinary Medicine, University of Ljubljana. Mice were conventionally housed in open polycarbonate cages (Tecniplast, Buguggiate, Italy) and provided with wood chip bedding (Lignocel, Rettenmaier and Sons, Germany). Mice were kept under standard conditions, including a 12:12 dark-light cycle (LED, CCT 3000 k, with no more than 25 lx above the cage), a temperature range of 21–24 °C, and humidity between 50 and 60%. They were fed irradiated rodent diet with minimal levels of phytoestrogens (Teklad global 16% protein rodent diet, BN 2916, Envigo, Udine, Italy) and access to drinkable tap water *ad libitum*. The experimental design was evaluated by the National Ethics Committee for Animal Experiments and approved by the Veterinary Administration of the Republic of Slovenia, document no. U34401–10/2021/8, with amendments U34401–10/2021/13 and U34401–10/2021/1.

##### Protocol and Sample Collection

Donepezil hydrochloride (TCI Europe) was used as a positive control and vehicle (tap water) as a negative control. Four animals, two female and two male mice, were used for each substance and each control. A total of 16 animals were used (*n* = 16). The mice were administered a dose of 10 mg/kg of compound **2 HCl**, 10 mg/kg of compound **(** ***R*** **)-(−)-3 HCl** and 2 mg/kg of donepezil hydrochloride. The volume dose was 200 μL/20 g of mouse body weight. Compounds **2 HCl** and **(** ***R*** **)-(−)-3 HCl** were dissolved in tap water to obtain a final concentration of 1 mg/mL, donepezil hydrochloride was dissolved in tap water to obtain a final concentration of 0.2 mg/mL, and the vehicle was administered at the appropriate volume dose. Oral administration was performed as intragastric gavage with flexible plastic feeding tubes (20 ga × 30 mm, Instech Laboratories, USA). After 1 h, blood was collected under terminal 5% isoflurane inhalation anesthesia (Isoflurin, VetPharma, Spain) from the left ventricle using a syringe. The blood was collected in a microtube (Eppendorf, Safe-Lock tube, 0.5 mL, Eppendorf AG, Germany) and allowed to clot. After approximately 1 h, the blood was then centrifuged at 3000 rpm for 15 min at 4 °C to obtain the serum. The brain was removed from the cavity and frozen in liquid nitrogen. The serum and brain samples were stored at −80 °C for further analysis.

#### Sample Analysis

##### Blood Plasma Sample Preparation

To each 150 μL of plasma, 800 μL of 2% (v/v) ortho-phosphoric acid was added and vortexed for 30 s. The 30 mg Bond-Elut Plexa PCX solid phase extraction cartridges (Agilent, Santa Clara, CA, USA) were preconditioned by 2 mL CH_3_OH, followed by 2 mL of Milli-Q water. Afterward, 850 μL of diluted plasma sample was added to the cartridges, which was washed by 2 mL of 2% HCOOH and 2 mL of a mixture of CH_3_OH/CH_3_CN (1:1, v/v) dried for 10 min, and eluted with 1 mL of 5% ammonia in a mixture of H_2_O/CH_3_OH/CH_3_CN (20:40:40, v/v). The eluted samples were dried at 60 °C in a TurboVap (Caliper, LS, Hopkinton, MA, USA) and reconstituted in 200 μL of 80% aqueous CH_3_OH solution with 0.1% AcOH, transferred to autosampler vials and subjected for LC-MS/MS analysis.

##### Brain Sample Preparation

Approximately 150 mg of brain sample was accurately weighed and transferred to an Eppendorf microtube (2 mL), followed by the addition of an extraction solvent comprising of 300 μL of 2% (v/v) ortho-phosphoric acid, 75 μL of CH_3_OH, 50 μL of internal standard solution (224 ng/mL haloperidol in 80% aqueous CH_3_OH solution). The homogenization was performed by the addition of 1.5 measuring spoons of 0.5 mm glass beads and 3 min treatment in Bullet-blender 24 homogenizer (Next Advance, NY, USA) at the intensity setting “8”. Afterward, 450 μL of 2% (v/v) ortho-phosphoric acid was added, briefly vortexed, and centrifuged at 10,000× rcf for 5 min. The first withdrawn supernatant of 850 μL was transferred to a 5 mL Eppendorf tube, and to the remaining sediment sample, 800 μL of fresh 80% aqueous CH_3_OH solution with 0.1% AcOH was added, homogenized under the same conditions again for 1 min, centrifuged and the supernatant of 800 μL was combined with the first one, to both of which, 900 μL of 2% (v/v) ortho-phosphoric acid was added, vortexed, and 2500 μL of this sample were processed by the same solid phase-extraction protocol as described for blood plasma samples.

##### Method Calibration

The quantitation was based on matrix-matched calibration performed by spiking known amounts of standards dissolved in 80% aqueous CH_3_OH solution before the extraction to either 150 mg of brain tissue or 150 μL of blood plasma in the concentration ranges of 0.2–200 ng per g of tissue, and 0.2–200 ng/mL, respectively.

##### Instrumental Analysis

The sample extracts were analyzed on LC-MS/MS instrument comprising of Agilent Infinity II liquid chromatograph (Agilent, Santa Clara, CA, USA) and Sciex 5500 plus QTRAP hybrid ion trap-quadrupole mass spectrometer (SCIEX, Ontario, Canada), operated in positive electrospray ionization mode. The chromatographic separation was performed on a Poroshell 120 EC C18 50 mm × 2.1 mm column with 1.9 μm particles (Agilent Technologies, Santa Clara, CA, USA) maintained at 45 °C and using a linear gradient of mobile phases A and B, consisting of 0.1% formic acid in Milli-Q water and CH_3_CN, respectively, with the following steps (time, %B): (0 min, 10%; 0.2 min, 10%; 1.0 min, 38%; 2 min, 50%; 2.5 min, 90%; 2.7 min, 10%). The Turbo V ion source parameters were: curtain gas 16 psi, ionspray voltage 3500 V, temperature 650 °C, Ion source gas 1 (nebulizer) 30 psi, and Ion source gas 2 (heater gas) 36 psi. The quantification was performed in multiple reaction monitoring mode (Table S4).

##### Assay Validation

ICH M10 guideline was used as a basis for quantitation method development and performance assessment. The working range for all three analytes was established over a wide concentration range from 0.2 to 200 ng/mL for plasma and from 0.6 to 200 ng/g for brain tissue, where a bias of less than 15% has been demonstrated for all calibrators above the LLOQ, and a determination coefficient (*R* ^2^) of 0.999 or better. The accuracy, precision, matrix effect, extraction recovery, and stability were checked by analyzing the separately prepared QC (quality control) samples at low, medium, and high-concentration levels in multiplicates. The accuracy and precision data are presented in Table S5. The lower quantitation limits were set as the lowest calibrator concentration exhibiting an accuracy of 80–120% and precision better than 20% RSD, together with a signal-to-noise ratio of at least 10:1 and a signal-to-blank of at least 5 (Figure S2). Absolute matrix effect (ME), extraction recovery (RE), and process efficiency (PE) were determined by the pre- and postsample preparation spike method. Namely, three different sets of samples were prepared in triplicates on three QC levels: set A: the analytes spiked in pure reconstitution solvent, corresponding to 100% recovery of the three QC-levels; set B: postspiked analytes in the extracted blank matrix with the same concentration as in set A; set C: the prespiked QC samples in matrix. The matrix effect was calculated as ME = B/A – 1; RE was calculated as C/B and PE was calculated as C/A, and expressed in percentages (Table S6). The relative matrix effect investigation could not be performed in this study due to the lack of blank animal lots of matrix. However, in another study, using the same extraction and quantitation method, a lack of any relative matrix effect has been demonstrated for compound **2**, as the determined matrix factor RSDs of only 7.23 and 1.60%, have been observed for QC-low and QC-high, respectively, in 6 different lots of blood plasma. During quantitation, the internal standard haloperidol response has been omitted due to its lack of any benefits and even a marginal negative impact on precision, as it has been observed in other similar situations where the isotopically labeled compounds were not available as internal standards.^101^ Nevertheless, as can be seen in Table S6, the absolute matrix effect was low, and the PE was consistent across the calibrated range.

#### In Vivo Activity Assays (Scopolamine-Induced Cognitive Dysfunction and Rotarod Test)

##### Animals and Housing Conditions

Adult male Albino Swiss (CD-1) mice weighing between 18 and 22 g and purchased from the Animal Breeding Farm of the Faculty of Pharmacy, Jagiellonian University Medical College were used in the PA and NOR tasks.

Animals were housed in cages (10 mice per cage) at a constant temperature of 22 ± 2 °C, humidity of 55 ± 10% and a light/dark (12:12) cycle. The mice had unlimited access to food and water prior to the experiment. Specified conditions for the maintenance of mice were ensured throughout the experiments, including tree bedding (Transwior, Poland) and cage enrichment (tunnels, nesting material, wooden igloos, etc.). For the behavioral test, the mice were selected randomly; each group consisted of 7–10 mice. The experiments were performed between 9 AM and 3 PM. After in vivo tests, the animals were immediately euthanized. The procedures for maintenance and treatment of laboratory animals were approved by the first Local Ethics Committee of the Jagiellonian University in Krakow (Approval No. 524/2021) and the treatment of animals was in full accordance with ethical standards laid down in respective Polish and EU regulations (Directive 2010/63/EU).

##### Compounds and Dose Selection

To induce learning and memory deficits scopolamine hydrobromide (Sigma-Aldrich, Poland) was used at the dose of 1 mg/kg. This dose was chosen based on our previous studies^30,102^ and available literature data.^103^ For the in vivo tests scopolamine hydrobromide was dissolved in distilled water (Polfa Kutno, Poland) and was administered subcutaneously (s.c.) 30 min before behavioral tests during the acquisition (training) trial of PA and NOR tasks. Compound **(** ***R*** **)-(−)-3 HCl** was suspended in 1% Tween 80 (Sigma-Aldrich, Poland) and administered intraperitoneally (i.p.) 60 min before the acquisition trial of the behavioral tasks. In the rotarod test compound **(** ***R*** **)-(−)-3 HCl** was also injected i.p. 60 min before the test. In the PA and NOR tasks cognition-enhancing properties of 2 doses of compound **(** ***R*** **)-(−)-3 HCl** were assessed: 1 and 10 mg/kg.

#### Behavioral Testing Paradigms

##### Passive Avoidance Task

In order to assess the influence of compound **(** ***R*** **)-(−)-3 HCl** on fear-motivated learning and memory in mice, the PA test was conducted according to a previously described protocol used in our laboratory.^102^ The PA apparatus (Panlab Harvard Apparatus, Spain) consists of a large white-colored illuminated compartment (26 cm × 26 cm × 34 cm) and a small black-colored compartment (13 cm × 7.5 cm × 7.5 cm) which are separated from each other by a guillotine gate (5 cm × 5 cm). The PA task is divided into two trials (i.e., the acquisition trial and the retention trial) which are conducted 24 h apart.

On the first day, during the acquisition trial (a conditioning phase), the test compounds (scopolamine hydrobromide, compound **(** ***R*** **)-(−)-3 HCl** and vehicle) were injected. Then, each mouse was placed into the white compartment, and a 30-s habituation period started (guillotine gate is closed). After that, the guillotine gate was opened, and the mice were tested during the 180-s testing period. As soon as the mouse entered the black compartment of the PA device, the guillotine gate was closed and an electrical shock (intensity: 0.2 mA, duration: 2 s) was automatically applied through the grid floor. For each mouse the latency between opening of the guillotine gate and mouse’s entering the black compartment was measured. On the next day, during the retention (drug-off) trial, the mice were placed again into the white compartment for a 30-s habituation period and the latency to enter the black compartment was measured for each mouse. Increased latency to enter the black compartment in the retention trial compared to that in the acquisition trial was a measure of a compound’s cognition enhancing properties.

##### Novel Object Recognition Task

The protocol used to perform the NOR task was adapted from the original work of Ennaceur and Delacour^104^ with some minor modifications to adapt this method for mice.^105^ The experiment was conducted in opaque black boxes with dimensions of 50 × 50 × 50 cm. The 3-day procedure consists of habituation to the test arena (without any objects) for 10 min on the first day (T0), a training trial (T1) and a testing trial (T2) separated by a 24-h intertrial interval. During T1 (familiarization trial), two identical objects (A and B) were presented in the opposite corners of the arena, approximately 5 cm from its walls. During T2 (recognition trial), one of the objects (B) was replaced by a novel object (C), so that the animals were presented with A (familiar) and C (novel) objects. Both T1 and T2 trials lasted for 10 min and the animals were returned to their home cages after T1. White plastic round containers (height 5 cm, diameter 5 cm) attached to the ground with double-sided tape were used as the A, B objects and green cuboids of similar height were used as objects C. The objects were stable enough so that the animals could not displace them. The sequence of presentations and the location of the objects were randomly assigned to each mouse. The animals explored the objects by looking, licking, sniffing, or touching them while sniffing, but not when leaning against, standing, or sitting on the objects. Any mouse exploring the two objects for less than 5 s within the 10 min duration of T1 or T2 was eliminated from the study. An experimenter, blind to the drug treatment, measured the exploration time of the objects. Based on exploration time (*E*) of the two objects during *T*_2_, discrimination index (DI) was calculated according to the formula: DI = (EC – EA)/(EA + EC) × 100. Using this metric, scores approaching zero reflect no preference, while positive values reflect preference for the novel object and negative numbers reflect preference for the familiar object.

##### Rotarod Test

Three days before the rotarod test, the mice were trained on the rotarod apparatus (Rotarod apparatus, May Commat RR0711, Turkey; rod diameter: 2 cm) that was rotated at a fixed speed of 18 rpm. In each training session, the mice were placed on the rotating rod for 3 min with an unlimited number of trials. The proper experiment was performed 24 h after the last training trial. Sixty min after the administration of compound **(** ***R*** **)-(−)-3 HCl** or vehicle, the mice were tested on the rod that revolved at 6, 18, and 24 rpm. Motor impairments in mice were defined as the inability to remain on the rotarod apparatus for 1 min. The results are expressed as the mean time spent on the rotarod.^106^

##### Data Analysis

For the analysis of data obtained in the PA, NOR and rotarod tasks, GraphPad Prism 9 (GraphPad Software, USA) was used. Numerical results from the tests were expressed as mean ± standard error of the mean (SEM). Repeated measures analysis of variance (ANOVA), one-way ANOVA followed by Dunnett’s *post hoc* test, or Student’s *t* test were used for the statistical analysis of the results. *P* < 0.05 was considered significant.

#### In Vivo Activity Assays (Aβ_1–42_-Induced Cognitive Deficits)

##### Animals and Housing Conditions

The experiments were performed using ICR male mice (8–10 weeks old, weight 18–20 g), which were purchased from Shanghai Bikai Animal Breeding Factory (Shanghai, China). Animals were housed in cages (6–8 mice per cage) at a constant temperature of 25 ± 2 °C, humidity of 55 ± 10% and a light/dark (12:12) cycle. The mice had unlimited access to food and water prior to the experiment. Specified conditions for the maintenance of mice were ensured throughout the experiments, including tree bedding (Yizheng Anlimao Biotechnology Co., Ltd., China) and lab feed (Zimao, China). For the behavioral test, the mice were selected randomly. The experiments were performed between 9 AM and 3 PM. After in vivo tests, the animals were immediately euthanized. These animal experiments were performed in the Pharmaceutical Laboratory Animal Center of China Pharmaceutical University (IACUC number: SYXK (SU) 2023–0018) in accordance with the regulations of Animal Ethics Committee of Chinese Academy of Medical Sciences and Materia Medica Research Institute and under the guidance of the National Institutes of Health’s Guidelines for the Care and Use of Laboratory Animals and were approved by the Center for New Drug Evaluation and Research, China Pharmaceutical University (Nanjing, China) (Ethics Approval Number 2024–11–11).

##### Compounds and Doses

To induce cognitive dysfunction recombinant human 1,1,1,3,3,3-hexafluoro-2-propyl alcohol pretreated Aβ_1–42_ (Merck Millipore, Darmastadt, Germany) was used. Aβ_1–42_ was dissolved in DMSO and prepared with normal saline at the final concentration of 2 mg/mL, and incubated for 72 h to prepare Aβ_1–42_ oligomers (olig.). Rivastigmine tartrate (Bidepharm, China) was dissolved in 1% DMSO (with applied ultrasound), diluted 50-times with PEG400 (with applied ultrasound), and finally diluted twice with saline (with applied ultrasound) to produce the final solution. Compounds **2** and **(** ***R*** **)-(−)-3** (used as **2 HCl** and **(** *R* **)-(−)-3 HCl**, respectively) were dissolved in saline.

The mice were divided into 6 groups with 6 mice in each group: (i) Negative control group of mice received normal saline via i.p. injection, (ii) AD model group of mice received olig. Aβ_1–42_ (10 μg per mouse) via i.c.v. injection and normal saline via i.p. injection, (iii) sham-operated group of mice received i.c.v. injection of normal saline via i.c.v. and i.p. injection, (iv) positive control group of mice received i.c.v. injection olig. Aβ_1–42_ (10 μg per mouse) via i.c.v. injection and rivastigmine (1 mg/kg) via i.p. injection, (v) experimental group of mice received olig. Aβ_1–42_ (10 μg per mouse) via i.c.v. injection and compound **2 HCl** (10 mg/kg) via i.p. injection, and (vi) experimental group of mice received olig. Aβ_1–42_ (10 μg per mouse) via i.c.v. injection and compound **(** ***R*** **)-(−)-3 HCl** (10 mg/kg) via i.p. injection. During 14 days of continuous administration of test substances, the body weight of all mice was monitored daily and the MWM task was performed on days 9–14. Finally, the mice were euthanized on day 14.

##### MWM Task Paradigm

The MWM (120 cm in diameter and 60 cm in height) is a pool filled with water set at 23 ± 1 °C. It is virtually divided into four equal quadrants with the third quadrant of the water maze being the target quadrant in which during the acquisition phase (days 1–5) of the MWM task an escape platform (10 cm in diameter, 40 cm in height) invisible to the swimming animal was immersed 1 cm under the surface of water.

The behavioral study of each mouse consisted of 5 days of spatial learning (acquisition) phase and a sixth day being a drug-off, retention phase. During the spatial acquisition trial mice were assigned to training sessions (four training sessions a day; sessions were held 4 h apart) in which the mice were trained to escape from water by reaching a hidden platform whose location could be identified using distal extra-maze cues (A4-size sheets of black laminated paper with color geometric symbols) attached to the room walls and constituted navigation points. Visual cues had different colors and dimensions and were kept constant during the whole experiment. For each trial the animal was placed in the water starting from a different randomly chosen quadrant that did not contain the platform, whereas the platform was always positioned in the same place. The time taken to reach the hidden platform (escape latency) on days 1–5 were recorded for each mouse. If a mouse did not find the platform’s place within 60 s, it was given a latency score of 60 s and was gently placed on the platform for 15 s.

In the retention trial the platform was removed from the pool and a probe trial was performed (without drug treatment–assessment of memory retention). On day 6, for each mouse the following parameters were recorded and measured: swimming paths of a mouse to find the former platform location and latencies to reach the target zone (i.e., the former platform location). In this phase of the MWM task, the cutoff was 180 s.

The time spent searching for the missing platform and trajectories were recorded by Xeye Aba camera (Beijing, China) and analyzed using SMART 3.0 (Panlab–Harvard Apparatus, Spain) and GraphPad Prism 5 (GraphPad Software, USA).

#### In Vivo Toxicity Assay

##### Animals and Housing Conditions

Mice used in this assay were around 3 months old female wild-type Balb/c mice bred in the Laboratory of Animal Genomics at the Faculty of Veterinary Medicine, University of Ljubljana. Mice were conventionally housed in open polycarbonate cages (Tecniplast, Buguggiate, Italy) and provided with wood chip bedding (Lignocel, Rettenmaier and Sons, Germany). Mice were kept under standard conditions, including a 12:12 dark-light cycle (LED, CCT 3000 k, with no more than 25 lx above the cage), a temperature range of 21–24 °C, and humidity between 50–60%. They were fed irradiated rodent diet with minimal levels of phytoestrogens (Teklad global 16% protein rodent diet, BN 2916, Envigo, Udine, Italy) and access to drinkable tap water *ad libitum*. The experimental design was evaluated by the National Ethics Committee for Animal Experiments and approved by the Veterinary Administration of the Republic of Slovenia, document no. U34401–10/2021/8, with amendments U34401–10/2021/13 and U34401–10/2021/1.

##### Protocol

For this assay, the number and sex of animals were determined according to the OECD guidelines for acute toxicity tests.^107^ Compound **(** ***R*** **)-(−)-3 HCl** was initially tested on 3 animals. As all animals survived within 14 days, the test was continued with 3 more animals to confirm the safety of the dose. A total of six female animals were used for this study (*n* = 6). Based on the in vitro data, it was considered that a dose ten times higher than the intended therapeutic dose (10 mg/kg) should still be safe and have no apparent toxicity. Therefore, a dose of 100 mg/kg was chosen.

Compound **(** ***R*** **)-(−)-3 HCl** was dissolved in tap water at a concentration of 10 mg/mL. Approximately 4 h prior to administration of the solution of **(** ***R*** **)-(−)-3 HCl**, the animals had no access to food while water was available. After the fasting period, the animals were weighed and the test substance was administered as intragastric gavage using flexible plastic feeding tubes (20 ga × 30 mm, Instech Laboratories, USA). The volume dose was 200 μL/20 g of mouse body weight. After administration, the animals were not fed for a further hour. The mice were observed regularly for the first 24 h, with particular attention to the first 4 h, and then daily for a total of 14 days. Observations included changes in body weight, skin and fur, mucous membranes and behavior. Particular attention was paid to the appearance of severe signs of the nervous system, such as Tremors, twitching, salivation, diarrhea, lethargy, sleep and coma. At the end of the 14-day observation period, all experimental animals were humanely euthanized with CO_2_ and subjected to necropsy.

##### Necropsy and Histopathological Analysis

After the toxicity assay a complete necropsy was immediately performed. Samples of stomach, small intestine (jejunum), cecum, colon, liver, kidney, adrenal gland, brain, spleen, lung and heart were collected from each mouse for histopathologic analysis. In addition, other organs with gross lesions were sampled. All samples were fixed in 10% neutral buffered formalin and routinely embedded in paraffin. Four-micrometre-thick tissue sections were deparaffinized, stained with hematoxylin and eosin (HE), and examined with a light microscope. The extent of histopathological lesions was subjectively assessed according to the following criteria:

0 = no lesions, 1 = mild lesions, 2 = moderate lesions, and 3 = severe lesions.

## Supplementary Material





## Notes

Photographs of the analyzed crystals of **(** ***S*** **)-(+)-3** and **(** ***R*** **)-(−)-3);** representative LLOQ LC-MS/MS chromatograms for compounds **2**, **(** ***R*** **)-(−)-3,** and donepezil in brain extracts; results of the histopathologic examination of mice organs after a toxicity test with 100 mg/kg **(** ***R*** **)-(−)-3**); crystallographic data for **(** ***S*** **)-(+)-3** and **(** ***R*** **)-(−)-3**); mass spectrometry parameters for detection and quantification of compound **2**, compound **(** ***R*** **)-(−)-3**, donepezil and haloperidol; accuracy and precision data for compound **2**, compound **(** ***R*** **)-(−)-3,** and donepezil in blood plasma and brain tissue; and the determined absolute matrix effect, recovery and process efficiency parameters for the quantification of compound **2**, compound **(** ***R*** **)-(−)-3**, donepezil and haloperidol from blood plasma and brain tissue (PDF)
Molecular formula strings contain molecular structures of compounds and associated activity data (CSV)

## Notes

9I02: **(** ***S*** **)-(+)-3**; 9I03: **(** ***R*** **)-(−)-3**. Authors will release the atomic coordinates and experimental data upon article publication.

## Abbreviations Used

Aβ: amyloid-β
ACh: acetylcholine
AChE: acetylcholinesterase
AD: Alzheimer’s disease
BChE: butyrycholinesterase
BTCh: butyrylthiocholine
BOC: *tert*-butyloxycarbonyl
Cbz: benzyloxycarbonyl
CCD: canine cognitive dysfunction
ChEs: cholinesterases
CHO: Chinese hamster ovary
ClogP: calculated logarithm of the octanol/water partition coefficient
CNS: central nervous system
DI: discrimination index
DMF: dimethylformamide
DMSO: dimethyl sulfoxide
DTNB: 5,5′-dithiobis­(2-nitrobenzoic acid)
FBS: fetal bovine serum
hAChE: human acetylcholinesterase
hBChE: human butyrycholinesterase
i.c.v.: intracerebroventricular
i.p.: intraperitoneal
MES: 2-(*N*-morpholino)­ethanesulfonic acid
MTS: 3-(4,5-dimethylthiazol-2-yl)-5-(3-carboxymethoxyphenyl)-2-(4-sulfophenyl)-2*H*-tetrazolium, inner salt
MW: molecular weight
MWM: Morris water maze
nAChRs: nicotinic acetylcholine receptors
NOR: novel object recognition
NRB: number of rotatable bonds
PA: passive avoidance
*P*_app_: permeability values
PBS: phosphate-buffered saline
PDB: protein data bank
rpm: rotations per minute
RSD: relative standard deviation
SARs: structure–activity relationships
s.c.: subcutaneous
SEM: standard error of the mean
SR: selectivity ratio
TBTU: 2-(1*H*-benzotriazole-1-yl)-1,1,3,3-tetramethylaminium tetrafluoroborate
TCh-TNB: thiocholine-thionitro benzoic acid
TFA: 2,2,2-trifluoroacetic acid
THF: tetrahydrofuran
tPSA: topological surface area
