# Supplementary material for: Lead Optimization of a Butyrylcholinesterase Inhibitor for the Treatment of Alzheimer’s Disease
Source: J Med Chem. 2025 Jun 2;68(11):11693–723. doi: 10.1021/acs.jmedchem.5c00577 (PMC12169612; doi:10.1021/acs.jmedchem.5c00577)
Supplement: Supplementary file 1 [file jm5c00577_si_001.pdf]

Supporting Information

Lead Optimization of a Butyrylcholinesterase Inhibitor for the Treatment of Alzheimer's Disease

Urban Košak,[‡] Nika Strašek Benedik,[‡] Damijan Knez,[‡] Simon Žakelj,[‡] Jurij Trontelj,[‡] Anja Pišlar,[‡] Selena Horvat,[‡] Aljoša Bolje,[‡] Neža Žnidaršič,[¤] Neža Grgurevič,[¤] Tanja Švara,[ϴ] Jakob Kljun,[□] Anna Skrzypczak-Wiercioch,[Δ] Bingbing Lv,[ʘ] Yucheng Xiong,[ʘ] Qinjie Wang,[†] Rui Bian,[ʘ] Jikuan Shao, [ʘ] José Dias,[ø] Florian Nachon,[ø] Xavier Brazzolotto,[ø] Jure Stojan,[▪] Haopeng Sun,[ʘ] Kinga Sałat,[▲] Stanislav Gobec[‡,*]

[‡]Faculty of Pharmacy, University of Ljubljana, Aškerčeva cesta 7, 1000 Ljubljana, Slovenia

[¤]Institute of Preclinical Sciences, Veterinary Faculty, University of Ljubljana, Ljubljana, Slovenia, Gerbičeva 60, 1000 Ljubljana

[ϴ]Institute of Pathology, Wild Animals, Fish and Bees, Veterinary Faculty, University of Ljubljana, Gerbičeva 60, 1000, Ljubljana, Slovenia

[□]Faculty of Chemistry and Chemical Technology, University of Ljubljana, Večna pot 113, 1000 Ljubljana, Slovenia

[Δ]University Centre of Veterinary Medicine JU-UA, University of Agriculture in Krakow, 24/28 Mickiewicza St., 30-059, Krakow, Poland

[ʘ]School of Pharmacy, China Pharmaceutical University, Nanjing, 211198, People's Republic of China

[†]School of Pharmacy, Nanjing University of Chinese Medicine, Nanjing, 210023, People's Republic of China

[ø]Institut de Recherche Biomédicale des Armées, Département de Toxicologie et Risques Chimiques, 91220 Brétigny sur Orge, France

[▪]Institute of Biochemistry, Faculty of Medicine, University of Ljubljana, Vrazov trg 2, 1000 Ljubljana, Slovenia

[▲]Faculty of Pharmacy, Jagiellonian University Medical College, 9 Medyczna St., 30-688 Krakow, Poland

***Corresponding Author:** Stanislav Gobec
Faculty of Pharmacy, University of Ljubljana, Aškerčeva cesta 7, 1000 Ljubljana, Slovenia
Tel: +386-1-4769500; Fax: +386-1-4258031; E-mail: stanislav.gobec@ffa.uni-lj.si

Table of Contents

Figure S1. Photographs of the analyzed crystals of **(*S*)-(+)-3** (left) and **(*R*)-(–)-3** (right). S3

Figure S2. Representative LLOQ LC-MS/MS Chromatograms from Left to Right: Compound 2, Compound (*R*)-(–)-3 and Donepezil in Brain Extracts...S3

Table S1. Results of the Histopathologic Examination of Mice Organs after a Toxicity Test with 100 mg/kg (*R*)-(–)-3. ....S4

Table S2. Crystallographic Data for (*S*)-(+)-3 and (*R*)-(–)-3. ....S4

Table S3. Data Collection and Refinement Statistics of hBChE in Complex with Compound (*S*)-(+)-3 and Compound (*R*)-(–)-3. ....S5

Table S4. Mass Spectrometry Parameters for Detection and Quantification of Compound 2, Compound (*R*)-(–)-3, Donepezil and Haloperidol....S6

Table S5. Accuracy and Precision Data for Compound 2, Compound (*R*)-(–)-3 and Donepezil in Blood Plasma and Brain Tissue. S7

Table S6. The Determined Absolute Matrix Effect, Recovery and Process Efficiency Parameters for the Quantification of Compound 2, Compound (*R*)-(–)-3, Donepezil and Haloperidol from Blood Plasma and Brain Tissue. S7

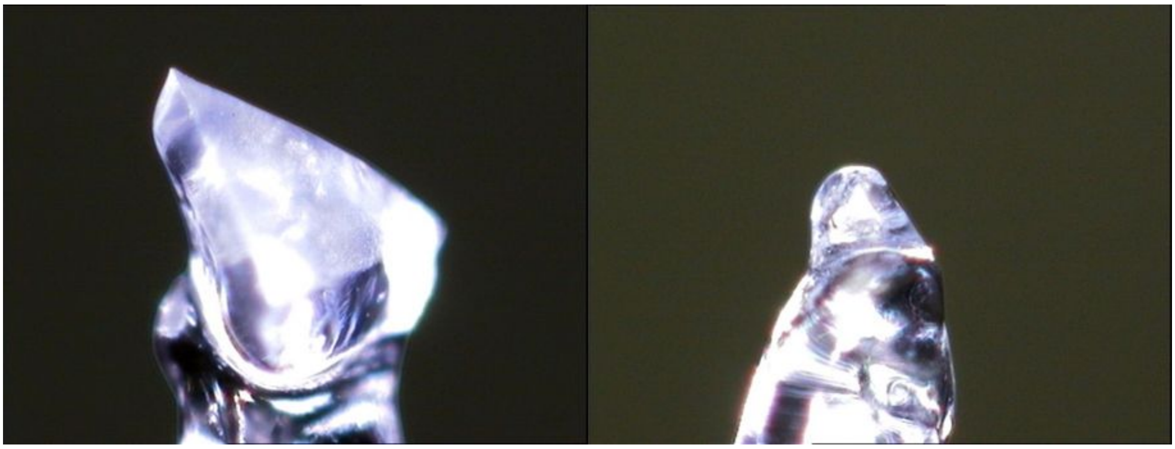

Figure S1. Photographs of the analyzed crystals of **(*S*)-(+)-3** (left) and **(*R*)-(–)-3** (right).

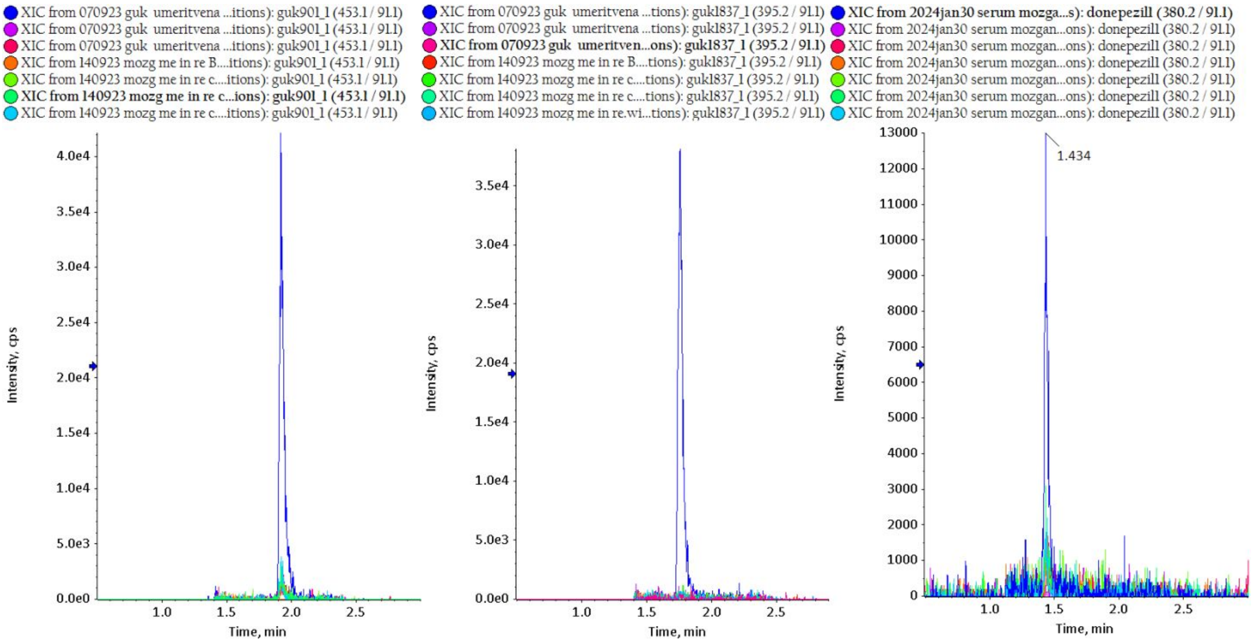

Figure S2. Representative LLOQ LC-MS/MS Chromatograms from Left to Right: Compound 2, Compound (*R*)-(–)-3 and Donepezil in Brain Extracts. Blue chromatogram trace represents the analyte in LLOQ samples while other colors represent blank samples.

Table S1. Results of the Histopathologic Examination of Mice Organs after a Toxicity Test with 100 mg/kg (*R*)-(–)-3. The table shows the number of animals with histopathological lesions and the number of all animals in each group.

Organ/ Histopathological lesion	Number of mice with the lesion	Mice					
		No. 1	No. 2	No. 3	No. 4	No. 5	No. 6
**Small intestine**							
Infiltrates of lymphocytes in the mucosa	6/6	1	2	2	2	2	1
**Caecum**							
Infiltrates of lymphocytes and single eosinophils in the mucosa	3/6	1	1	1	0	0	0
Lymphocyte aggregates in the mucosa	5/6	0	1	1	2	1	1
**Colon**							
Infiltrates of lymphocytes and single eosinophils in the mucosa	2/6	1	0	0	1	0	0
**Liver**							
Infiltration of mononuclear cells in the sinusoids	5/6	0	1	1	1	1	1
Fatty infiltration of the hepatocytes	5/6	1	2	2	1	0	2
Hepatocyte kariomegaly	2/6	0	0	1	0	0	1
Hepatocyte hypertrophy	1/6	0	0	0	0	1	0
Dilatation of bile ducts and infiltration of lymphocytes in the portal areas	1/6	0	0	0	0	1	0
**Kidney**							
Infiltration of mononuclear cells in the interstitium	3/6	1	0	1	0	0	1
**Adrenal gland**							
Accessory cortical nodule	2/6	1	0	1	0	0	0
**Heart**							
Perivascular infiltrates of mast cells	2/6	0	1	0	0	1	0
Infiltration of lymphocytes in the myocardium	1/6	0	1	0	0	0	0
**Uterus**							
Cystic endometrial hyperplasia	1/1	ND	ND	1	ND	ND	ND

0 = no lesions, 1 = mild lesions, 2 = moderate lesions, and 3 = severe lesions; ND = not done

Table S2. Crystallographic Data for (*S*)-(+)-3 and (*R*)-(–)-3.

Compound	**(*S*)-(+)-3**	**(*R*)-(–)-3**
CCDC No.	2423398	2423399
Sample code	**Moc440**	**Moc441**
Empirical formula	$C_{23}H_{26}N_2O_2S$	$C_{23}H_{26}N_2O_2S$
Formula weight	394.52	394.52
Temperature/K	150.00(10)	150.00(10)
Crystal system	orthorhombic	orthorhombic
Space group	$P2_12_12_1$	$P2_12_12_1$

a/Å	10.0358(3)	10.0390(3)
b/Å	11.4369(4)	11.4363(3)
c/Å	18.1556(6)	18.1667(6)
α/°	90	90
β/°	90	90
γ/°	90	90
Volume/$Å^3$	2083.87(12)	2085.70(11)
Z	4	4
ρ_{calc} g cm^{-3}	1.257	1.256
μ/mm^{-1}	0.176	0.176
F(000)	840.0	840.0
Crystal size/mm^3	0.6 × 0.4 × 0.4	0.4 × 0.3 × 0.2
Radiation	Mo Kα (λ = 0.71073)	Mo Kα (λ = 0.71073)
2Θ range for data collection/°	5.4 to 54.958	4.636 to 54.962
Index ranges	-13 ≤ h ≤ 12, -14 ≤ k ≤ 14, -23 ≤ l ≤ 23	-13 ≤ h ≤ 12, -14 ≤ k ≤ 14, -22 ≤ l ≤ 23
Reflections collected	35850	36295
Independent reflections	4741 [R_{int} = 0.0337, R_{sigma} = 0.0202]	4746 [R_{int} = 0.0355, R_{sigma} = 0.0220
Data/restraints/parameters	4741/0/254	4746/0/254
Goodness-of-fit on F^2	1.055	1.054
Final R indexes [I ≥ 2σ (I)]	R_1 = 0.0300, wR_2 = 0.0729	R_1 = 0.0304, wR_2 = 0.0709
Final R indexes [all data]	R_1 = 0.0347, wR_2 = 0.0765	R_1 = 0.0374, wR_2 = 0.0753
Largest diff. peak/hole / e $Å^{-3}$	0.19/-0.25	0.18/-0.23
Flack parameter	-0.04(2)	-0.007(19)

Table S3. Data Collection and Refinement Statistics of hBChE in Complex with Compound (*S*)-(+)-3 and Compound (*R*)-(–)-3. Table Calculated Using Phenix.

	**hBChE compound (*S*)-(+)-3**	**hBChE compound (*R*)-(–)-3**
Data collection		
**X-ray source**	ESRF BM07	ESRF BM07
**Wavelength**	0.9795	0.9795
**Resolution range**	48.91 - 2.58 (2.672 - 2.58)	48.57 - 2.562 (2.654 - 2.562)
**Space group**	I 4 2 2	I 4 2 2
**Unit cell**	154.67 154.67 127.34 90 90 90	153.6 153.6 127.11 90 90 90
**Total reflections**	176429 (16852)	170886 (17014)
**Unique reflections**	23919 (2276)	24153 (2387)

**Multiplicity**	7.4 (7.4)	7.1 (7.1)
**Completeness (%)**	97.21 (94.28)	97.72 (98.35)
**Mean I/sigma(I)**	10.21 (1.25)	13.31 (1.30)
**Wilson B-factor**	57.18	57.10
**R-merge**	0.1405 (1.364)	0.1132 (1.391)
**R-meas**	0.151 (1.469)	0.1221 (1.502)
**R-pim**	0.0536 (0.5264)	0.04445 (0.551)
**CC1/2**	0.997 (0.469)	0.998 (0.507)
**CC***	0.999 (0.799)	1 (0.82)
Refinement statistics		
**Reflections used in refinement**	23902 (2274)	24140 (2386)
**Reflections used for R-free**	1196 (113)	1208 (119)
**R-work**	0.1795 (0.2840)	0.1853 (0.3217)
**R-free**	0.2390 (0.3245)	0.2373 (0.3469)
**CC(work)**	0.968 (0.758)	0.968 (0.730)
**CC(free)**	0.931 (0.770)	0.922 (0.782)
**Number of non-hydrogen atoms**	4623	4635
**macromolecules**	4240	4238
**ligands**	284	294
**solvent**	99	103
**Protein residues**	527	526
**RMS(bonds)**	0.072	0.058
**RMS(angles)**	1.40	1.39
**Ramachandran favored (%)**	94.67	94.08
**Ramachandran allowed (%)**	5.33	5.53
**Ramachandran outliers (%)**	0.00	0.38
**Rotamer outliers (%)**	1.31	2.41
**Clashscore**	6.41	7.41
**Average B-factor**	64.41	67.62
**macromolecules**	62.20	64.92
**ligands**	100.30	109.02
**solvent**	56.06	60.61
**Number of TLS groups**	1	1

R-work=Σ |Fo - |Fc|| / Σ |Fo|, where Fo and Fc are observed and calculated structure factors, respectively. R-free set uses about 10% randomly chosen reflections. Statistics for the highest-resolution shell are shown in parentheses.

Table S4. Mass Spectrometry Parameters for Detection and Quantification of Compound 2, Compound (*R*)-(–)-3, Donepezil and Haloperidol.

**Compound**	**Q1 *m/z***	**Q3 *m/z* quantifier/ qualifier**	**DP (V)**	**EP (V)**	**CE (eV) quantifier/ qualifier**	**CXP (V) quantifier/ qualifier**
**2**	453.1	91.1 / 127.0	110	10	67 / 55	15 / 20
**(*R*)-(–)-3**	395.2	91.1 / 127.0	110	10	67 / 55	15 / 20
Donepezil	380.2	91.1 / 243.2	60	10	59 / 37	10 / 14
Haloperidol	376.1	123.1 / 165.0	135	10	55 / 30	15 / 15

DP, declustering potential; EP, entrance potential; CE, collision energy; CXP, cell exit potential.

Table S5. Accuracy and Precision Data for Compound 2, Compound (*R*)-(–)-3 and Donepezil in Blood Plasma and Brain Tissue.

	Quality control level	Concentration levels (ng/g) / (ng/mL)	Compound 2 accuracy % (RSD%)	Compound (*R*)-(–)-3 accuracy % (RSD%)	Donepezil accuracy % (RSD%)
Blood plasma	Low	0.6	98.9 (5.1)	92.5 (2.9)	107.7 (5.0)
	Medium	15	99.7 (5.4)	100.9 (4.1)	89.4 (6.2)
	High	150	89.0 (1.8)	85.3 (3.2)	102.1 (5.0)
Brain	Low	0.6	89.4 (5.1)	88.5 (13.7)	113.3 (2.9)
	Medium	15	92.1 (7.1)	96.0 (8.8)	99.8 (12.1)
	High	150	105.0 (2.7)	108.8 (1.5)	99.7 (7.4)

Table S6. The Determined Absolute Matrix Effect, Recovery and Process Efficiency Parameters for the Quantification of Compound 2, Compound (*R*)-(–)-3, Donepezil and Haloperidol from Blood Plasma and Brain Tissue.

Compound		Blood plasma			Brain tissue		
	QC level	ME (%)	RE (%)	PE (%)	ME (%)	RE (%)	PE (%)
	Low	-23.0	85.3	65.6	0.7	66.3	66.8
**2**	Medium	-16.5	74.8	62.5	-13.8	67.4	58.1
	High	-11.0	68.0	60.5	-14.1	65.0	55.8
	Low	-30.9	81.5	56.3	-44.1	80.4	44.9
**(*R*)-(–)-3**	Medium	-25.8	73.9	54.9	-22.5	65.0	50.4
	High	-16.1	61.7	51.8	-23.6	60.5	46.2
	Low	17.2	31.9	37.4	-13.2	44.6	38.7
**Donepezil**	Medium	-11.8	43.6	38.4	-47.3	77.0	40.5
	High	-14.6	53.6	45.7	-37.8	70.0	43.5
**Haloperidol**	one level	-13.8	78.7	67.8	-33.7	52.0	34.5

ME, matrix effect; RE, recovery; PE, process efficiency.
